# Genome-Wide and Phenotypic Evaluation of Stem Cell Progenitors Derived From *Gprc5a*-Deficient Murine Lung Adenocarcinoma With Somatic *Kras* Mutations

**DOI:** 10.3389/fonc.2019.00207

**Published:** 2019-04-02

**Authors:** Reem Daouk, Maya Hassane, Hisham F. Bahmad, Ansam Sinjab, Junya Fujimoto, Wassim Abou-Kheir, Humam Kadara

**Affiliations:** ^1^Department of Biochemistry and Molecular Genetics, Faculty of Medicine, American University of Beirut, Beirut, Lebanon; ^2^Department of Anatomy, Cell Biology and Physiological Sciences, Faculty of Medicine, American University of Beirut, Beirut, Lebanon; ^3^Department of Translational Molecular Pathology, The University of Texas MD Anderson Cancer Center, Houston, TX, United States

**Keywords:** lung adenocarcinoma, *Kras*, stem cell progenitors, *Gprc5a*, lung cancer pathogenesis

## Abstract

Lung adenocarcinomas (LUADs) with somatic mutations in the *KRAS* oncogene comprise the most common molecular subtype of lung cancer in smokers and present with overall dismal prognosis and resistance to most therapies. Our group recently demonstrated that tobacco carcinogen-exposed mice with knockout of the airway lineage G-protein coupled receptor, *Gprc5a*, develop LUADs with somatic mutations in *Kras*. Earlier work has suggested that cancer stem cells (CSCs) play crucial roles in clonal evolution of tumors and in therapy resistance. To date, our understanding of CSCs in LUADs with somatic *Kras* mutations remains lagging. Here we derived CSCs (as spheres in 3D cultures) with self-renewal properties from a murine *Kras*-mutant LUAD cell line we previously established from a tobacco carcinogen-exposed *Gprc5a*^−/−^ mouse. Using syngeneic *Gprc5a*^−/−^ models, we found that these CSCs, compared to their parental isoforms, exhibited increased tumorigenic potential *in vivo*, particularly in female animals. Using whole-transcriptome sequencing coupled with pathways analysis and confirmatory PCR, we identified gene features (*n* = 2,600) differentially expressed in the CSCs compared to parental cells and that were enriched with functional modules associated with an augmented malignant phenotype including stemness, tumor-promoting inflammation and anti-oxidant responses. Further, based on *in silico* predicted activation of GSK3β in CSCs, we found that tideglusib, an irreversible inhibitor of the kinase, exhibited marked anti-growth effects in the cultured CSCs. Our study underscores molecular cues in the pathogenesis of *Kras*-mutant LUAD and presents new models to study the evolution, and thus high-potential targets, of this aggressive malignancy.

## Introduction

Non-small cell lung cancer (NSCLC) is the leading cause of cancer-related deaths worldwide (1). Lung adenocarcinoma (LUAD), squamous cell carcinoma, and large cell carcinoma constitute the major histological subtypes of NSCLC (2, 3). LUAD represents the most common subtype of NSCLC and is prevalent in never, former and current smokers (2, 3). LUADs are further molecularly subtyped based on molecular features and driver alterations (4). The GTPase v-Ki-ras2 Kirsten rat sarcoma (*KRAS*) is the most commonly somatically mutated oncogene in LUAD (~ 25–30% of LUADs) (4, 5). Relative to other NSCLCs, *KRAS*-mutant LUADs are clinically very aggressive, display dismal prognosis and are resistant to most, if not all, therapies (4, 5). These data suggest the pressing need for new strategies for the clinical management and treatment of this fatal disease.

Cancer is a hierarchically heterogeneous cell population that is governed by a small subset of cells termed cancer stem cells (CSCs) or tumor initiating cells (6). CSCs are thought to be associated with the aggressive behavior of cancer cells through their stem cell-like properties of self-renewal, tumor initiation and propagation, and generation of the differentiated cells that constitute the bulk of the tumor (7). CSCs are also thought to be responsible for therapy resistance and tumor recurrence, therefore targeting CSCs could be an effective strategy for cancer treatment (7, 8). The stem cells or progenitors that drive the evolution of LUAD are thought to be distinct and dependent on the specific molecular subtype of the disease (9). CSCs of LUADs with somatic mutations in *KRAS*, and that would thus be ideal targets for therapy, are still not well-defined or clearly understood. Various reports have shown controversial results. By utilizing mouse models genetically engineered to express mutant *Kras*, various groups have suggested stem progenitors that may be implicated in the pathogenesis of this malignancy. Kim et al. suggested that a regional bronchioalveolar stem cell population may be involved in *Kras*-driven LUAD formation (10) and independent reports underscored progenitor populations responsible for *Kras* mutant LUAD initiation that most likely arise from alveolar type 2 cells (11, 12). Despite these insights, the biology of CSCs in LUADs with somatically acquired *Kras* mutations (e.g., by tobacco carcinogen) remains under-studied.

We have recently shown that *Gprc5a*^−/−^ mice develop LUADs with somatic mutations in *Kras* (13), tumors whose pathogenesis is still not clear. Also, while CSCs are known to play crucial roles in the clonal evolution of tumors, their role in the development of LUADs with somatic mutations in *KRAS*, such as those arising in the *Gprc5a*^−/−^ mouse, is poorly understood. To fill this void, we here derive new experimental models constituting CSCs from *Gprc5a*^−/−^ *Kras*-mutant LUAD cells and utilize those cells to identify phenotypic and genome-wide gene expression features that underlie the pathogenesis of this aggressive molecular subtype of lung cancer.

## Materials and Methods

### Cell Culture

The murine *Gprc5a*^−/−^ *Kras*-mutant LUAD (MDA-F471) (14) and human *KRAS*-mutant LUAD (H1792) (ATCC, USA) cell lines were maintained in a humidified incubator at 37°C with 5% CO_2_ and in DMEM F-12 medium (Sigma-Aldrich) supplemented with 10% Fetal Bovine Serum (FBS) (Sigma-Aldrich), 1% penicillin-streptomycin antibiotics (Lonza), and 5 μg/ml Plasmocin Prophylactic (InvivoGen). For passaging, cells were enzymatically dissociated using 0.05% Trypsin-EDTA solution (Sigma-Aldrich) and maintained for a maximum of 25 passages.

### Sphere Formation Assay

Derivation of CSCs as 3D spheres was performed as described previously (15, 16). Three biological replicates of single cell suspensions of MDA-F471 or H1792 cells were embedded in growth factor-reduced Matrigel™ (Corning) in a 1:1 ratio with serum-free medium at a concentration of 2,000 cells/well in a total volume of 50 μl and plated uniformly around the rim of wells of a 24-well plate in duplicates and allowed to solidify for 45 min at 37°C in a humidified incubator. Subsequently, 500 μl of warm medium (5% FBS) was added gently in the middle of each well and was replenished with new fresh medium every 2 to 3 days. After 6 to 7 days, spheres were counted and the sphere forming unit (SFU) was calculated as follows: SFU = 100 × (number of formed spheres/2,000). Bright field images of formed spheres were acquired using Zeiss Axiovert inverted light microscope, and the diameter of 30 spheres per condition was recorded. To serially propagate the spheres, Matrigel™ was digested by the addition of 500 μl of 0.5 mg/ml dispase (Gibco) solution dissolved in growth medium in each well and incubation for 45 min in a humidified incubator at 37°C. Spheres were then dissociated into single cells with 0.05% Trypsin-EDTA at 37°C. Cells were resuspended in serum-free medium, counted and re-plated as explained before. Propagation was repeated four times until the fifth generation of spheres (G5).

### Subcutaneous Xenotransplants

The *Gprc5a*^−/−^ mouse was generated and obtained from the University of Texas MD Anderson Cancer Center (17). The obtained mice were bred and maintained in the Animal Care Facility (ACF) at the Faculty of Medicine of the American University of Beirut (AUB-FM) and all experiments were conducted in accordance with current regulations of The Institutional Animal Care and Use Committee (IACUC). All mice were housed in individually ventilated cages (IVCs) with free access to food and water. All experiments were performed on 8–10 weeks old *Gprc5a*^−/−^ mice. To assess tumor growth of MDA-F471 cells in this syngeneic model, three different cell dilutions (500,000; 10,000; 1,000) of either parental cells or dissociated spheres were suspended in 200 μl of a 1:1 mixture of serum-free medium and growth factor-reduced Matrigel™. Each dilution was subcutaneously injected into the flanks of mice (6 males and 6 females in each group; per cell type and per dilution). Mice were monitored every other day for the formation of palpable tumors, after which tumor size was measured three times per week using a digital caliper and recorded as follows: Tumor size (mm^2^) = length × width. At the conclusion of the experiment, mice were euthanized by CO_2_ inhalation or cervical dislocation 4 weeks after tumor cell injections, or when tumor size reached ~1.5 cm in diameter.

### Total RNA Extraction

Total RNA was purified from parental cells or spheres using the RNeasy Plus kit (Qiagen) according to the manufacturer's instructions. Concentrations of RNA samples were quantified using the DS-11 FX spectrophotometer (DeNovix) according to the manufacturer's protocol. The 260/280 ratio was used to assess the purity of RNA and a ratio of ~2.0 was considered as pure RNA.

### Whole-Transcriptome Sequencing Analysis

Whole-transcriptome sequencing (RNA-Seq) of MDA-F471 parental and first generation (G1)-derived spheres (*n* = 3 biological replicates each) was performed using the NovaSeq 6000 platform (Illumina). The Ribo-zero RNA removal kit was used to remove ribosomal RNAs from total RNA samples. Paired-end libraries (101 bp reads) were prepared from 500 ng total RNA using the TruSeq stranded total RNA LT sample kit (Illumina) according to the manufacturer's instructions. Sequenced raw reads were first subjected to quality control (QC) using FastQC. Trimmomatic (18) was used to remove adapter sequences, low quality bases as well as reads with lengths shorter than 36 base pairs. Reads were then mapped to the reference mouse genome (UCSC mm 10) using the fast splice-aware aligner HISAT2 (19). Transcripts were then assembled from aligned reads using StringTie (20) borrowing from the annotation database RefSeq_2017_06_12. Normalization was performed using DESEQ2 (21) considering both transcript length and depth of coverage. Read counts were then computed for each transcript/gene.

For differential expression analysis, first a pseudocount of one was applied to all genes/transcripts and samples; this ensures analysis of non-zero counts. Identification of gene features significantly differentially expressed between the MDA-F471 G1 spheres and parental isoforms was performed using DESEQ2 (21) in the R language and environment and using a false discovery rate (FDR) threshold of 1% and a random variance model. A fold-change threshold of 2 was further applied. Differentially expressed gene features (*n* = 2,600) were then functionally analyzed and topologically organized into gene-gene interaction networks using the commercially available software Ingenuity Pathways Analysis (IPA).

### Two-Step Quantitative Real-Time Polymerase Chain Reaction (qRT-PCR)

Total RNA samples from *Gprc5a*^−/−^ CSCs and parental cells, as well as those of the human H1792 cell line (three biological replicates from each line), were reverse transcribed to cDNA using the QuantiTect Reverse Transcription Kit (Qiagen) according to the manufacturer's protocol. The PCR reaction was carried out with iTaq Universal SYBR Green Supermix (Bio-Rad) using the BioRad CFX96 RT-PCR detection system. The thermal cycling conditions were composed of an initial denaturation step at 95°C for 5 min, followed by 40 cycles of denaturation at 95°C for 15 s, annealing for 30 s and extension at 72°C for 30 s. A melt curve was included at the end of each reaction to verify the specificity of the product. Expression in the spheres relative to parental cell lines was analyzed using the 2^−ΔΔ*Ct*^ method and by normalization to the average of two reference genes: *Gapdh* and *Tbp*. Each reaction was done in biological triplicates and technical duplicates. The murine and human primer sequences as well as their annealing temperatures are included in Tables S1, S2, respectively.

### Flow Cytometry-Based Analysis of Aldehyde Dehydrogenase Activity

The ALDEFLUOR kit (StemCell Technologies) was used to identify and quantify MDA-F471 subpopulations with high pan-aldehyde dehydrogenase (ALDH) enzymatic activity. Single cells from the parental line or dissociated spheres (*n* = 3 biological triplicates each) were suspended in ALDEFLUOR assay buffer at a concentration of 100,000 cells per 500 μl. A volume of 2.5 μl of the fluorescent ALDH substrate was added to each sample. For each cell type, a negative control sample was prepared containing 5 μl of the ALDH specific inhibitor diethylaminobenzaldehyde (DEAB). Cells were incubated for 45 min at 37 °C then spun for 5 min to pellet and resuspended in cold ALDEFLUOR buffer. Samples were then taken for flow cytometry analysis on the Guava® easyCyte flow cytometer (Millipore). Propidium iodide (PI) (0.5 μg/ml) was added to each sample just before flow cytometry to stain for dead or late apoptotic cells. Each sample was gated and analyzed according to its own negative control with DEAB. Gating and analysis were performed according to the strategy demonstrated in Figure S2.

### Treatment of Spheres With Tideglusib

MDA-F471 or H1792 cells were cultured in Matrigel™ (2,000 cells/well) for 6 to 7 days and propagated to G2 as described previously (15, 16) (*n* = three independent experiments for each condition with two technical duplicates for each experiment). Three tideglusib (Sigma-Aldrich) concentrations (1, 5, and 10 μM) and the vehicle control (0.02% DMSO) were prepared in the spheres' growth medium (DMEM-F12 + 5% FBS) and 500 μl was added gently in the middle of each well in triplicates per experiment. Spheres were replenished every 2 to 3 days with fresh medium and drug. After 6 to 7 days, spheres were counted and their SFUs were calculated. Bright field images of formed spheres were acquired using the Zeiss Axiovert inverted light microscope, and diameters of 30 spheres per condition was recorded.

### Colony Formation Analysis of Adherent Cells

Adherent cells were seeded at different cell densities (400, 200, and 100 cells/well for MDA-F471 and 500 cells/well for H1792) in complete growth medium (10% FBS) in triplicates in six-well plates and incubated overnight in a humidified incubator at 37°C. On the following day, cells were treated with the different concentrations of tideglusib (1, 5 or 10 μM) or with vehicle control (0.02% DMSO) in growth medium containing 5% FBS. The cells were replenished every 2 to 3 days with fresh media and drug. After 6 to 7 days, cells were fixed and stained with 1 ml 0.5% crystal violet solution in 25% methanol for 10 min. Plates were then washed gently with water and the number of colonies was counted manually for each well and the colony forming unit (CFU) was calculated as follows: CFU = 100 × (number of formed colonies/the original seeding density). Subsequently, the stained colonies were solubilized in 600 μl 1% sodium dodecyl sulfate (SDS) in water per well on a plate shaker for 1 h, then 100 μl were transferred from each well to a 96-well plate in triplicates and the optical density was measured on the Multiskan EX spectrophotometer (Thermo Scientific) at 595 nm.

### Statistical Analyses

All statistical tests were performed using GraphPad Prism 7 software. To determine statistical significance of differences in sphere diameters a one-way ANOVA test was performed. The non-parametric Kruskal-Wallis test was applied when analyzing SFUs and CFUs. For multiple comparisons, a Bonferroni (or Dunn for non-parametric) corrected test was used. Student's *t*-test was performed when analyzing gene expression and ALDH activity. Statistical analysis of xenograft tumor sizes between parental cells and spheres was performed by computing average areas under the curve (AUCs) for each group followed by statistical assessment of AUC differences between the groups using Student's *t*-test. All *P* < 0.05 were considered significant.

## Results

### Derivation and Assessment of Self-Renewal of CSCs From *Gprc5a*^−/−^ *Kras*-Mutant LUAD Cell Lines

**Figure 1:**
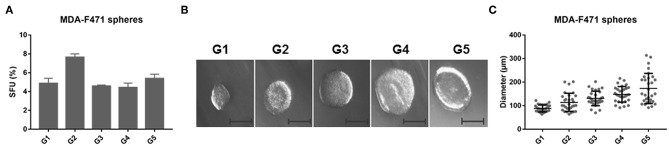
Sphere formation abilities of *Gprc5a*^−/−^ *Kras*-mutant LUAD cells. Single cell suspensions of *Gprc5a*^−/−^ MDA-F471 cells (see Materials and Methods) were embedded in Matrigel™ and plated at the rim of each well. Media containing 5% FBS was added in the middle of each well. Cells were incubated for 6–7 days until they formed spheres. Sphere forming units were calculated as percentages of the number of formed spheres relative to the number of seeded cells. To assess the self-renewal ability of sphere forming cells, spheres were propagated for up to five generations. **(A)** Sphere forming units (SFUs) of MDA-F471 cells across five generations. Error bars represent standard deviations between technical triplicates of each of three independent experiments. **(B)** Representative bright-field images of MDA-F471 G1-G5 spheres visualized by Axiovert inverted microscope at 10X magnification and analyzed by Carl Zeiss Zen 2 image software. Scale bar = 100 μm. **(C)** Scatter plots of the diameters (μm) of 30 spheres from G1 up to G5 for MDA-F471. Data were reported as mean ± SD (*P* < 0.0001 by one-way ANOVA).

Earlier work has shown that CSCs possess the capability of forming multicellular three-dimensional (3D) spheres *in vitro* when grown in non-adherent conditions, whereas more differentiated cells fail to thrive in such conditions (15). Our group recently demonstrated *Gprc5a*^−/−^ mice particularly upon tobacco carcinogen exposure developed LUADs with somatically acquired driver *Kras* mutations (13). The molecular pathogenesis of *Gprc5a*^−/−^ *Kras*-mutant LUADs is still elusive. In this study, we determined to derive and enrich for CSCs from *Gprc5a*^−/−^ *Kras*-mutant LUAD. We used a murine LUAD cell line we had previously isolated from a tobacco-carcinogen exposed *Gprc5a*^−/−^ mouse (MDA-F471 cells) (14). Single cell suspensions of MDA-F471 were cultured in Matrigel™ for 1 week until they formed the first generation of spheres (G1). Spheres were then subsequently propagated for up to five generations. We found that spheres were continuously maintained from G1 to G5 with dynamic sphere forming units (SFUs) ranging from 4.44 to 7.63% for MDA-F471 (Figure 1A). We also assessed the size of the spheres across the generations (Figure 1B). We found that the average sphere diameter gradually and statistically significantly increased from G1 to G5 (*P* < 0.0001; Figure 1C). These data suggest that CSCs derived from *Gprc5a*^−/−^ *Kras*-mutant LUAD cells possess self-renewal abilities.

### Assessment of *in vivo* Tumorigenicity of CSCs Derived From Murine *Gprc5a^−/−^ Kras*-Mutant LUAD Cells

**Figure 2:**
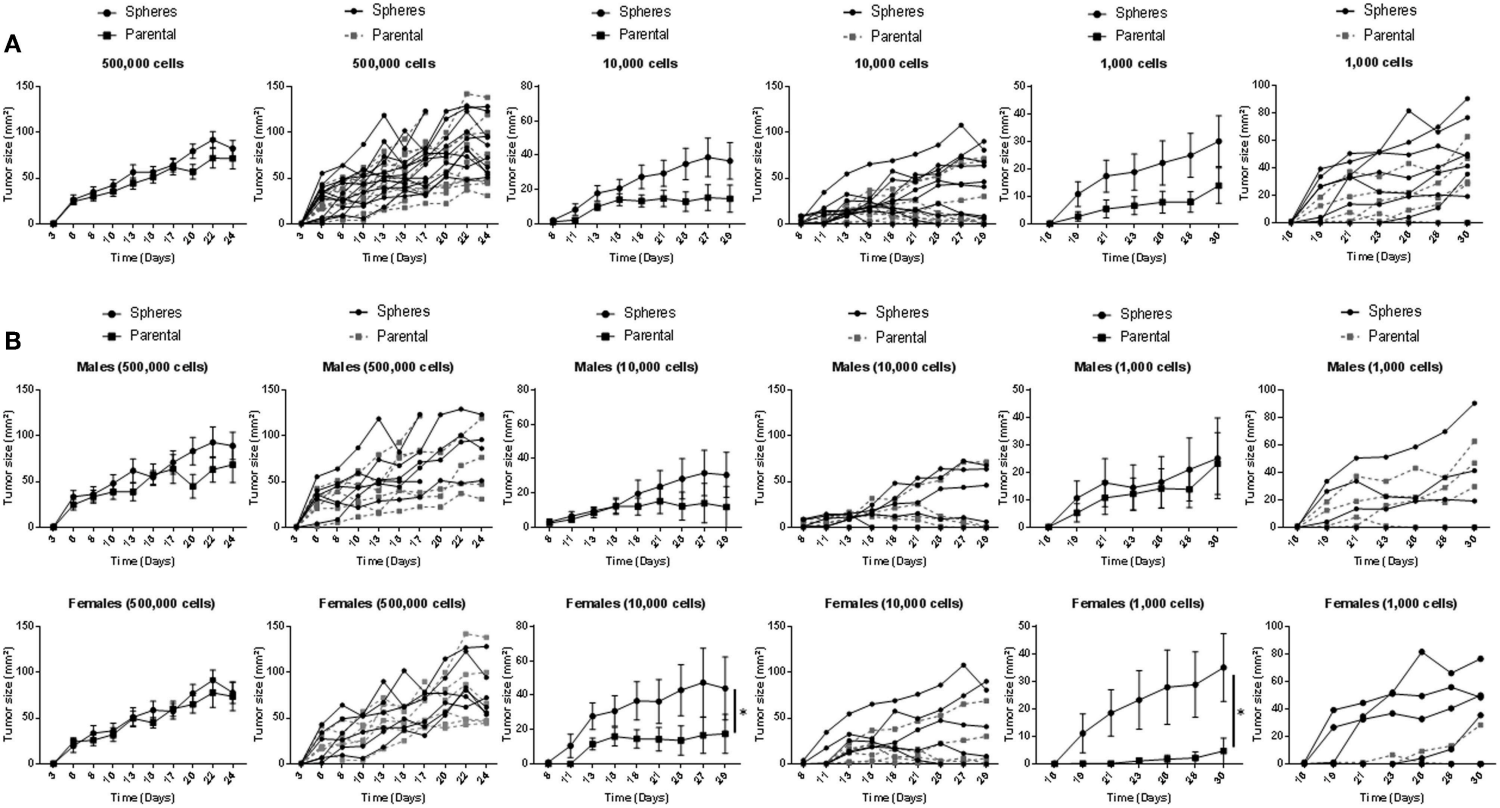
*In vivo* growth of *Gprc5a*^−/−^ *Kras*-mutant LUAD G1 spheres and parental cells. Three different cell dilutions (500,000; 10,000; 1,000) of either *Gprc5a*^−/−^ MDA-F471 parental cells or dissociated spheres were suspended in 200 μl of a 1:1 mixture of serum-free DMEM F-12 media and growth factor-reduced Matrigel™ and subcutaneously injected into the right flank of 11 to 12 mice per group and per xenotransplanted cell number. Lengths and widths of tumors were measured three times per week for 4 weeks and tumor sizes were calculated according to the formula: tumor size (mm^2^) = length x width. Tumor sizes are plotted in line graphs as means ± SEM (left) or as individual values per mouse (right). Analysis was performed on grouped mice **(A)** or following separating animal groups into female and male sub-groups **(B)**. Average values of areas under the curve (AUCs) were calculated for each group and differences between AUCs were statistically analyzed using Student's *t*-test (*P < 0.05).

Following our *in vitro* assays, we sought to investigate the *in vivo* tumorigenicity of *Gprc5a*^−/−^ MDA-F471 CSCs in a syngeneic setting. Tumor growth of MDA-F471 parental cells and dissociated G1 spheres was evaluated by subcutaneous injection of various cell numbers (500,000, 10,000, and 1,000 cells) into 11 or 12 *Gprc5a*^−/−^ mice per group (and per cell number). We found that xenotransplantation of 500,000 cells formed tumors in all mice and we did not observe notable and significant differences in tumor sizes between the xenotransplanted parental cells and G1 spheres at this cell number (Figure 2A). At lower cell numbers, differences in tumor forming capacity between spheres and parental cells became increasingly more apparent—a classical observation following *in vivo* CSC tumorigenicity experiments (22). At 10,000 cells per xenotransplant, there were trends for increased tumorigenicity (shorter time to tumor formation and larger tumor sizes) by the spheres relative to parental cells and this observation became more evident, albeit not reaching statistical significance at 1,000 cells (Figure 2A). It is worth noting that for the latter two cell dilutions, mice injected with dissociated G1 spheres retained more tumors at the end point as compared to mice injected with parental cells (Table S3). Next, we sought to analyze tumor development separately by sex. Similar to what we had observed previously for the 500,000 cell dilution, we did not find differences in tumor sizes between spheres and parental cells in male or female mice (Figure 2B). In contrast, when xenotransplanting 10,000 and 1,000 cells, we found that MDA-F471 G1 spheres developed significantly larger tumors relative to parental cells (*P* = 0.03 and *P* = 0.0223, respectively) in female mice only (Figure 2B).

We also evaluated the *in vivo* oncogenesis of fifth generation (G5) spheres derived from murine *Gprc5a*^−/−^ *Kras*-mutant LUAD cells. Similar to the G1 counterparts, xenotransplanted G5 spheres yielded significantly larger tumors than parental cells, specifically in female mice (*P* = 0.007 and *P* = 0.014 for the 10,000 and 1,000 cell dilutions, respectively) (Figure S1). Similar to what we had observed with G1 spheres, female mice injected with dissociated MDA-F471 G5 spheres retained more tumors at the end point than did female mice injected with parental cells, which was significant by Fisher's exact test for the 1,000 cell dilution (*P* = 0.0152) (Table S4). These findings suggest that CSCs we had derived from *Gprc5a*^−/−^ *Kras*-mutant LUAD cells exhibit stemness properties exemplified by their increased tumorigenic activity *in vivo* when compared to parental cells.

### Whole-Transcriptome Sequence Analysis of *Gprc5a*^−/−^ LUAD CSCs and Parental Cells

**Figure 3:**
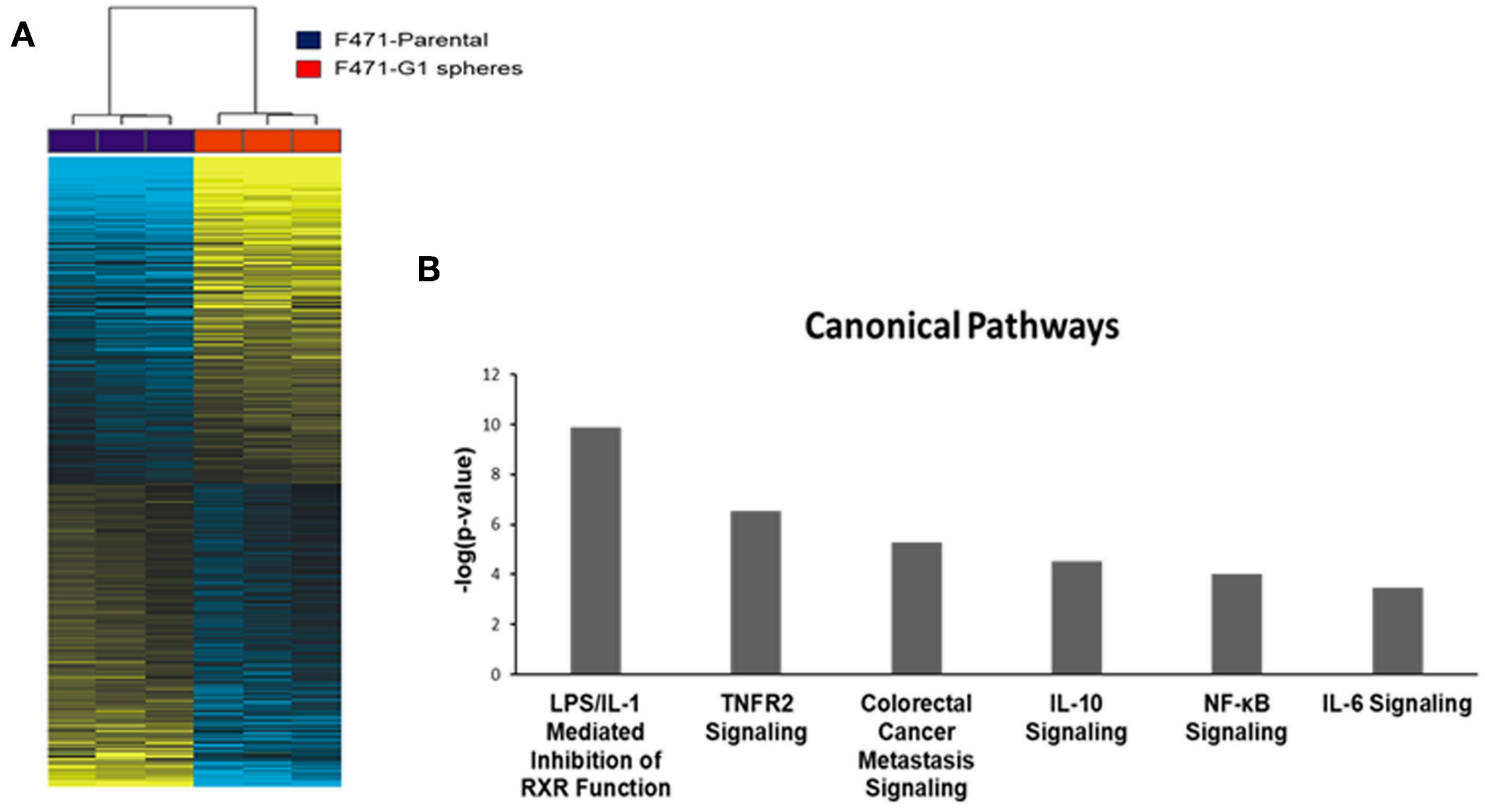
Whole-transcriptome sequence analysis of *Gprc5a*^−/−^ *Kras*-mutant LUAD G1 spheres and parental cells. RNA-Seq was performed using the NovaSeq 6000 platform from Illumina as described in the Materials and Methods section. **(A)** Differentially expressed transcripts (*n* = 2,600) between MDA-F471 G1 spheres and parental cells (three biological replicates/independent experiments in each group) were identified using statistical criteria detailed in the Materials and Methods section and analyzed by hierarchical clustering in the R statistical language and environment. Columns represent samples and rows indicate transcripts (yellow, up-regulated relative to the median; blue, down-regulated relative to the median). **(B)** Representative canonical pathways differentially modulated in MDA-F471 G1 spheres relative to their parental counterparts. Modulation of canonical pathways was identified by IPA (see Methods). Select pathways are ordered from left to right based on statistical significance (indicated by –log base 10 of the *P*-value).

We sought to study transcriptomic features that signify the CSC phenotype in *Kras*-mutant LUAD. We performed paired-end RNA-sequencing using the NovaSeq 6000 Illumina platform to delineate differentially expressed gene features between MDA-F471-derived G1 spheres and the parental cell line (three biological replicates in each group). On average, we sequenced ~52 million reads per sample. Using a false discovery rate (FDR) threshold of 1% (in a random variance model) and a two-fold change cut-off, we identified 2,600 transcripts that were significantly differentially expressed between the *Gprc5a*^−/−^ LUAD CSCs and parental cells (1,345 up-regulated and 1,255 down-regulated in the CSCs; Table S5). Hierarchical cluster analysis demonstrated that the identified differentially expressed gene features were able to effectively separately cluster the MDA-F471 G1 spheres from their parental cell counterparts (Figure 3A).

We were then prompted to compute the functional relevance of these differentially expressed transcripts using pathways and gene set analyses. Pathways analysis using Ingenuity Pathways Analysis (IPA) revealed significantly altered pathways (all *P* < 0.05) in MDA-F471 G1 spheres relative to parental cells (Table S6). These included LPS/IL-1 mediated inhibition of RXR function, TNFR2 signaling, metastasis signaling, and tumor-promoting inflammation signaling such as that mediated by IL-10, IL-6, and NF-κB (all *P* < 0.001) (Figure 3B). Using gene set enrichment analysis features by IPA, we also identified significantly activated or inhibited upstream regulators of the identified differentially expressed transcripts. These included marked activation (indicated by activation z-scores) of tumor-promoting mediators such as IL-1β, IFNγ, TNF, NF-κB, and RELA, transcription regulators of the antioxidant pathway such as FOXO3 and NFE2L2 (otherwise known as NRF2), as well as growth promoting kinases including glycogen synthase kinase 3 beta (GSK3β) (all z-scores > 2.0 and *P* < 0.0001) (Table S7).

### Increased Aldehyde Dehydrogenase Expression and Activity in *Gprc5a*^−/−^ LUAD CSCs Relative to Parental Cells

**Figure 4:**
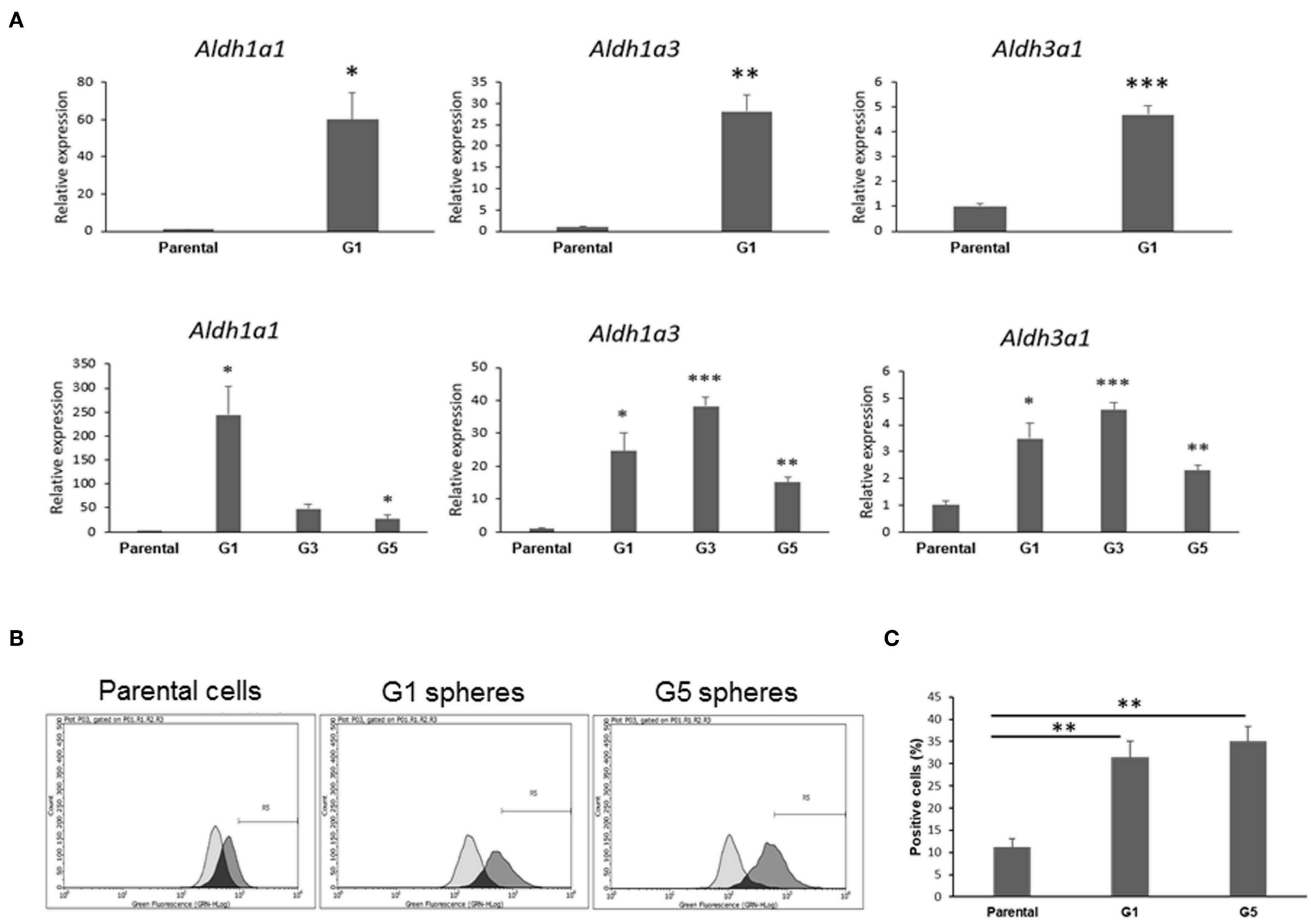
Validation of aldehyde dehydrogenase up-regulation and activation in MDA-F471 spheres relative to parental cells. **(A)** Up-regulation of *Aldh1a1, Aldh1a3* and *Aldh3a1* in MDA-F471 G1, G3, and G5 spheres was validated by qRT-PCR and analyzed using the 2^−ΔΔ*Ct*^ method by normalization to the average of two reference genes (*Gapdh* and *Tbp*). Relative expression values are presented as means + SEM (three biological replicates/independent experiments in each group and technical duplicates in each experiment) (*P < 0.05; **P < 0.01; ***P < 0.001 by Student's *t*-test). Pan-aldehyde dehydrogenase activity was measured in MDA-F471 parental cells, G1 and G5 spheres (three biological replicates/independent experiments each group) by the ALDEFLUOR kit and analyzed by flow cytometry. Cells treated with DEAB (ALDH inhibitor) were used as negative control to set the gate that would identify ALDH^+^ cells (see Materials and Methods). **(B)** Representative plots showing shifts in fluorescence intensity between control and test conditions for each cell type. **(C)** Quantification of the percentage of ALDH^+^ cells for parental cells, G1 and G5 spheres (three biological replicates each). Differences in ALDH^+^ cells between the three groups were statistically determined using Student's *t*-test (**P < 0.01).

Next, we pursued confirmation of gene features that were identified by the RNA-Seq analysis to be differentially expressed in *Gprc5a*^−/−^ LUAD CSCs relative to the parental cell counterparts. RNA-Seq analysis had revealed the up-regulation of various aldehyde dehydrogenase isozymes (Table S5), the most notable of which were *Aldh1a1, Aldh1a3*, and *Aldh3a1*, known to be important for maintenance and self-renewal of stem cells and that were reported to be widely over-expressed in CSCs from various tumor types (23). Consistent with the RNA-Seq results, quantitative real-time PCR analysis of the *Gprc5a*^−/−^ LUAD CSCs and parental counterparts (three biological replicates each) showed significant (all *P* < 0.05) up-regulation of the three aldehyde dehydrogenases in the CSCs (G1 spheres) compared to parental cells (Figure 4A, upper panels). This effect was similarly observed in independent qRT-PCR analyses of G3 and G5 spheres (Figure 4A, lower panels). In order to confirm these results at the functional level, we next performed flow cytometry analysis of pan-aldehyde dehydrogenase (ALDH) activity in dissociated G1 and G5 spheres as well as in the parental MDA-F471 cells. Using the ALDEFLUOR assay (see Materials and Methods), which permits identification and quantification of cells with high/positive pan-aldehyde dehydrogenase activity (Figure S2), we found significant elevated fractions of ALDH^+^ cells in both G1 and G5 spheres (31.4 and 35.1%, respectively) compared to parental cells (11.2%) (both *P* < 0.01; Figures 4B,C).

### Differential Gene Expression Profiles in *Gprc5a*^−/−^ LUAD CSCs Relative to Parental Cells

**Figure 5:**
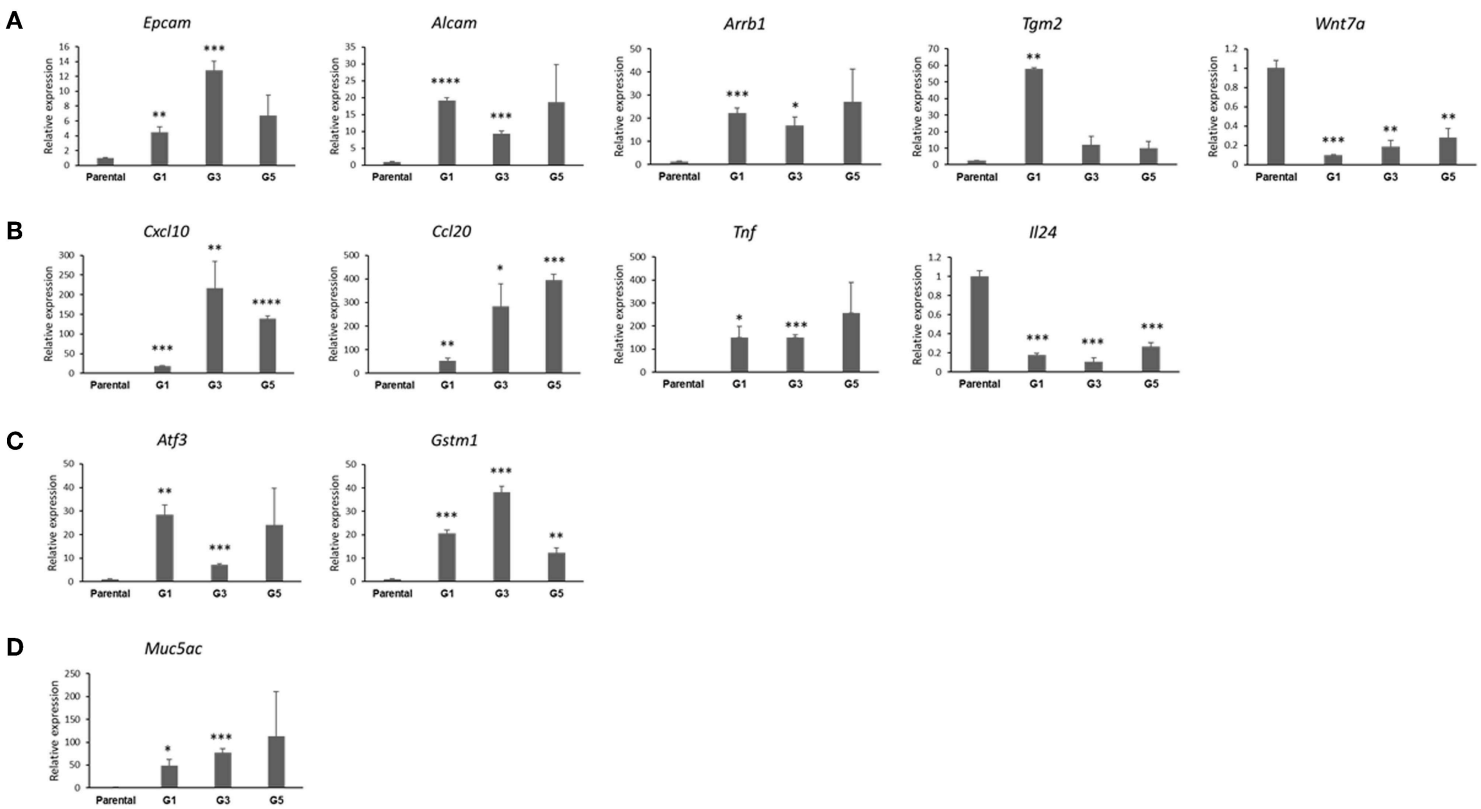
Confirmation of genes differentially expressed in MDA-F471 *Gprc5a*^−/−^ *Kras*-mutant LUAD spheres relative to parental cell counterparts. Differential expression of select genes MDA-F471 G1 spheres and parental cells (three biological replicates/independent experiments in each group and technical duplicates in each experiment) was confirmed by qRT-PCR and analyzed using 2^−ΔΔ*Ct*^ method by normalization to the average of two reference genes (*Gapdh* and *Tbp*). Genes studied and depicted in the figure include those pertinent to stemness self-renewal **(A)**, tumor-promoting inflammation and cytokines/chemokines **(B)**, anti-oxidant function **(C)** and tumor-promoting mucins **(D)**. Relative expression values are presented as means + SEM (n=3). (*P < 0.05; **P < 0.01; ***P < 0.001; ****P < 0.0001 by Student's *t*-test).

We then interrogated and confirmed genes by qRT-PCR based on their mode of differential expression (up-regulated vs. down-regulated in the *Gprc5a*^−/−^ LUAD G1 spheres), extent of differential expression as well as biological functional associations based on the functional pathways and gene set analyses. The qRT-PCR analysis demonstrated significantly increased expression of genes that are pertinent to the CSC phenotype in *Gprc5a*^−/−^ LUAD G1 spheres relative to parental cells (all Figure 5A). We found that the stem cell renewal and survival drivers/markers *Arrb1* and *Tgm2* were both markedly up-regulated in the MDA-F471 G1 spheres as well as subsequent sphere generations, particularly G3 and G5 compared to their parental counterparts (Figure 5A). We also noted, overall, significant up-regulation of the two lung epithelial stem cell markers *Epcam* and *Alcam* in G1, G3, and G5 spheres (Figure 5A). Conversely, we found that the *Wnt* family member *Wnt7a*, recently shown to have a tumor-suppressive role in lung cancer by inducing cellular senescence (24), was significantly suppressed in G1, G3, and G5 spheres relative to the parental cell counterparts (Figure 5A).

We also confirmed differential expression of other genes that are not directly related to stemness. Our qRT-PCR analysis demonstrated significant up-regulation of three tumor-promoting cytokines, *Cxcl10, Ccl20*, and *Tnf* and down-regulation of the tumor inhibitory (25) cytokine *Il24* in the MDA-F471 spheres relative to parental cells (Figure 5B). We also found that the *Gprc5a*^−/−^ LUAD spheres, overall, exhibited significantly up-regulated expression of two major members of the canonical Nrf2-antioxidant response element pathway, *Atf3* and *Gstm1* (Figure 5C). Further, we found up-regulation of a tumor-promoting mucin gene *Muc5ac* recently shown to be necessary for *Kras*-mutant lung cancer initiation (26) (Figure 5D). Of note, we validated several of these differentially expressed genes in G1 spheres we derived from the human LUAD cell line H1792 also with driver codon 12 *KRAS* mutations and which displayed similar sphere formation patterns as MDA-F471 cells as described previously (Figure S3). We found that the human H1792 G1 spheres also displayed up-regulation of *ALDH1A1* and *ALDH3A1* (Figure S4). Similarly, we also found significant up-regulation of the cytokines *CCL20* and *TNF* and the anti-oxidant gene *ATF3* in the human H1792 G1 spheres compared to their parental counterparts (Figure S4). These findings highlight molecular phenotypes (e.g., stemness, ALDH activity, tumor-promoting inflammation, and anti-oxidant function) that are prevalent in CSC-enriched spheres from *Gprc5a*^−/−^ LUAD with somatically acquired driver *Kras* mutations.

### Effect of an Irreversible GSK3β Inhibitor on *Gprc5a*^−/−^ *Kras*-Mutant Cells

**Figure 6:**
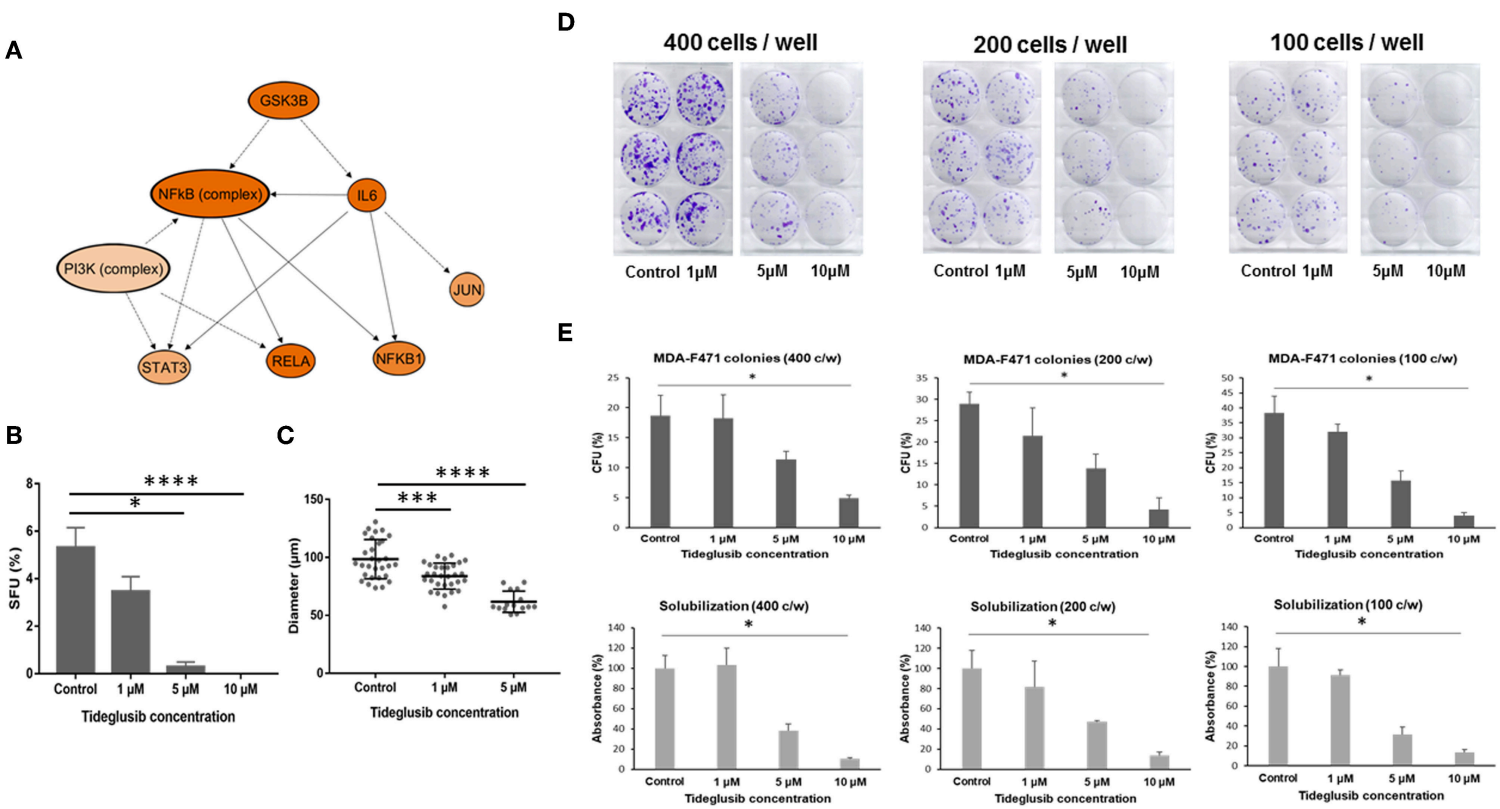
Effect of tideglusib on sphere and colony forming ability of *Gprc5a*^−/−^ *Kras*-mutant LUAD cells. **(A)** Predicted mechanistic gene-gene interaction network mediated by GSK3β as predicted by the *in silico* pathways analysis. The molecules shown in orange are predicted to be activated. Color intensity represents the level of activation. The solid and interrupted arrows represent direct and indirect interactions, respectively. MDA-F471 cells were cultured in Matrigel™ for 6 to 7 days. Formed spheres were propagated to G2 and treated with control (DMSO), 1, 5, and 10 μM of tideglusib every 2 to 3 days. **(B)** Sphere forming units (SFUs) were calculated for each condition as percentage of the number of formed spheres relative to number of seeded cells. Data were statistically analyzed by Dunn's *post-hoc* test following Kruskal-Wallis (for three independent experiments in each group with two technical duplicates in each experiment). **(C)** Diameters of 30 spheres per condition were measured using Carl Zeiss Zen 2 image software and analyzed by one-way ANOVA. Colony formation assay of adherent MDA-F471 cells was performed as described in the Materials and Methods section **(D–E)** Cells were seeded in triplicates in three seeding densities (400, 200 and 100 cells/well) in six-well plates and treated with varying concentrations of tideglusib every 2 to 3 days for 7 days. Scanned images of the cultured adherent cells are depicted in **(D)** Colonies were counted and colony forming units (CFUs) were calculated for each condition as follows: CFU = 100 x (number of colonies/the original seeding density) (**E**, upper panels). Spectrophotometric analysis of solubilized crystal violet-stained colonies was performed as described in the Methods section (**E**, lower panels). Differences in number of colonies and absorbance between the different conditions were analyzed using Dunn's *post-hoc* test following Kruskal-Wallis (*P < 0.05; ***P < 0.001; ****P < 0.0001).

In our coupled RNA-Seq and topological gene-gene interaction analyses, we found that one of the upstream regulators predicted to be activated in *Gprc5a*^−/−^ LUAD spheres was the kinase Gsk3β (Figure 6A). As depicted in the topologically organized mechanistic gene-gene interaction network, Gsk3β was computationally predicted to be upstream of major protumor inflammatory modules including by Nf-κb and Il-6. Given these findings, we thought that Gsk3β may be a viable target for these LUADs. To preliminarily test this supposition, we employed the irreversible Gsk3β inhibitor tideglusib, a drug in several clinical trials for its effects on stem cells and that has shown antiproliferative and antitumorigenic effects on brain tumors (27–29).

In order to test the effect of tideglusib on the sphere forming ability of MDA-F471 cells, we cultured the cells in Matrigel™ for 6 to 7 days to enrich for CSCs after which the formed spheres were propagated to the next generation and treated with varying concentrations of the drug. The number and size of MDA-F471 *Gprc5a*^−/−^ LUAD spheres significantly decreased in a dose-dependent manner (both *P* < 0.0001) (Figures 6B,C). Treatment with 1 μM of tideglusib decreased sphere formation, indicated by the SFUs, by ~35% (Figure 6B). Treatment with 5 μM tideglusib significantly and markedly decreased SFUs by ~95% (*P* < 0.05) and 10 μM of tideglusib completely abolished the sphere forming ability of the MDA-F471 *Gprc5a*^−/−^ LUAD spheres (*P* < 0.0001) (Figure 6B). Similarly, treatment with 1 μM of tideglusib significantly decreased the average sphere diameter by ~17% (*P* < 0.001), whereas the 5 μM concentration decreased diameters by ~35% (*P* < 0.0001) (Figure 6C). Of note, diameter sizes following treatment with 10 μM of tideglusib could not be assessed because there were no spheres observed at this dose (Figure 6C). We next sought to evaluate the anti-growth effects of tideglusib on the parental cell counterparts. In the context of comparably evaluating colony forming capacities, we seeded the parental cells at three different densities (400, 200, and 100 cells/well, each in triplicates). Parental cells were then treated with the same concentrations of tideglusib interrogated with the spheres (1, 5, and 10 μM) for 6 to 7 days. Tideglusib decreased the number of MDA-F471 parental colonies in a dose-dependent manner and at all the three seeding densities (*P* = 0.002 for 400 cells/well, *P* = 0.0028 for 200 cells/well and *P* = 0.0006 for 100 cells/well)—although it is noteworthy that this effect was less significant and substantiated than that observed with the spheres (Figures 6D,E). While 1 μM tideglusib significantly decreased forming units and diameters of the *Gprc5a*^−/−^ LUAD spheres (Figures 6B,C), this dose of tideglusib did not significantly decrease colony formation of MDA-F471 parental/adherent cells (Figures 6D,E). Also, while treatment with 5 μM tideglusib nearly abolished *Gprc5a*^−/−^ LUAD SFUs (Figure 6B), this dose reduced, albeit not statistically significantly, colony formation of the parental cell counterparts by 39–59% (across the three cell densities). Further and in sharp contrast to what was observed with the spheres, significant decreases in the colony formation of *Gprc5a*^−/−^ LUAD parental cells were only observed at 10 μM of tideglusib (73–89% across the three cell densities; *P* < 0.05; Figures 6D,E). Next we attempted to extend and validate these findings in human H1792 *KRAS*-mutant lung adenocarcinoma CSCs/spheres. Similar to *Gprc5a*^−/−^ LUAD spheres, tideglusib significantly decreased both H1792 sphere formation (Figure S5A; *P* < 0.01) and diameter (Figure S5B; *P* < 0.0001) in a dose-dependent manner. When treating the H1792 parental counterparts, there was a trend for reduced colony formation of the cells by tideglusib in a dose-dependent manner albeit modest and not reaching statistical significance (Figures S5C,D). These findings suggest that Gsk3β inhibition markedly attenuates the growth and self-renewal of CSCs derived from *Gprc5a*^−/−^ *Kras*-mutant LUAD. Our data also suggest that, pharmacological targeting of this kinase may serve as a viable strategy for the treatment of human *KRAS*-mutant lung adenocarcinoma malignancy.

## Discussion

Lung adenocarcinoma with mutations in the oncogene *KRAS* remains to be the most aggressive molecular subtype of lung malignancy with generally poor prognosis and resistance to most therapies. Development of effective early treatment strategies for this malignancy is hindered by our lagging knowledge of early molecular mechanisms that drive *KRAS-*mutant LUAD pathogenesis. Our group recently found that mice lacking *Gprc5a* and with tobacco carcinogen exposure develop LUADs with somatic mutations in *Kras* (13). The phenotypic evolution of these tumors in *Gprc5a*^−/−^ is still poorly understood. Several studies have identified cancer stem cells (CSCs) as drivers of tumor growth. In this study, we derived and cultured CSCs from *Gprc5a*^−/−^ *Kras*-mutant LUAD cells. We also performed phenotypic (*in vitro* and *in vivo*) and genome-wide gene expression characterization of these derived CSCs in comparison to their parental cell counterparts. CSCs from *Gprc5a*^−/−^ *Kras*-mutant LUAD cells exhibited self-renewal properties *in vitro* and displayed enhanced tumorigenesis relative to parental cells when xenotransplanted in *Gprc5a*^−/−^ mice, particularly in female animals. By coupled RNA-Seq and functional pathway analysis we underscored phenotypic and expression cues that embody a heightened malignant phenotype in CSCs from *Gprc5a*^−/−^ *Kras*-mutant LUAD cells including increased stemness, pro-tumor inflammation and antioxidant capacity. Our findings shed new light on the molecular pathogenesis of LUADs with somatic *Kras* mutations and, thus, on potential new therapeutic vulnerabilities in this aggressive malignancy.

Recent studies have demonstrated that sphere formation is effective in enrichment of cells with stem-like properties from both primary tumors and cell lines (15, 30, 31). In line with these studies, we utilized sphere formation assays to derive spheres from *Gprc5a*^−/−^ MDA-F471 *Kras*-mutant LUAD cells that are enriched for CSCs. These spheres comprised cells that survived at least five generations of sphere formation in Matrigel™ matrix, reflecting the self-renewal phenotype of these cell subpopulations, a major hallmark of stem/progenitor cells (32). We also showed that the derived spheres gradually increased in size across the generations perhaps owing to enrichment in the cell population that represents true stem-like cells rather than short lived progenitor cells that have the capacity to form spheres in early generations (16). It is worthwhile to mention that several methods in the past have been utilized to identify and isolate CSCs from different tumor types (30, 33, 34). The overexpression of surface markers, specific to normal tissue stem cells, were primarily used to isolate CSCs (30, 33, 34). Of note, studies have previously shown that cell surface marker profiling did not yield conclusive results in various cancers due to the observation that marker-negative cells also possessed the ability to form spheres *in vitro* and to give rise to aggressive tumors *in vivo* (33). Different reports showed that cells which do not express the stem cell marker CD133 can also exhibit stem cell-like tumorigenic properties and sphere formation abilities (35, 36), and while we found increased expression of some stem cell epithelial markers, our spheres were negative for CD133 (Table S5).

Xenotransplantation of CSCs of different types into immunocompromised mice has been widely used to characterize the capability of these cells to initiate and grow tumors in a more or less physiological environment unhindered by the rejection of the host's immune system (33). Yet, the significance of the tumor microenvironment has recently been gaining more attention (37). Tumor tissues naturally contain a large number of tumor-infiltrating immune cells that facilitate tumor formation (38). In our present study, we assessed the differential tumor growth between MDA-F471 CSCs and parental cells using a syngeneic model of the *Gprc5a*^−/−^ mouse. Generally, CSCs showed enhanced ability, particularly in female animals, to grow *in vivo* following xenotransplantation, evidenced by an increase in tumor incidence and tumor size as opposed to xenotransplants of parental cells. While, we have shown that these CSCs displayed a more tumorigenic phenotype *in vivo*, the role of the immune microenvironment, given we used a relatively unique syngeneic setting, is not known. It is reasonable to surmise that, aside from tumor initiation *in vivo*, the CSCs may exhibit differential interactions with the host immune response (e.g., evade immune surveillance and destruction) compared with the parental cells. Further studies are warranted to address this hypothesis that is couched by our findings. It is important to note that we performed xenotransplants subcutaneously which comprises an immune microenvironment that is partly distinct from that in the lung. Future studies centered on orthotopic xenotransplants are warranted to probe the interplay between CSCs in the lung and the host immune response. Nonetheless, a preliminary observation in our experiments that is in line with the above supposition is the lower number of CSC xenotransplants that “regressed” following growth (a feature of functional anti-tumor immunity *in vivo*) compared with transplanted parental cells.

We observed a host sex disparity/factor in the growth of sphere and parental cell xenotransplants. We found that even at low cell numbers, differential tumor growth in male mice was not significant between CSCs and parental cells, owing to the development of larger tumors from parental cells in male mice as compared to female littermates. These findings are in line with the report by White-Gilbertson and colleagues who showed sex-mediated disparity in growth of xenotransplanted bladder cancer cells (39). It is noteworthy that epidemiological and clinical studies have previously demonstrated that men are at a greater risk of developing lung cancer than women and relatively display poorer clinical outcomes (40). The mechanism underlying this gender disparity is still largely unknown. A recent study by Caetano and colleagues demonstrated that female animals, in the context of Il-6 and Stat3 pro-tumor inflammatory signaling, displayed differential anti-lung cancer immune responses, evidenced by levels of tumor infiltrating lymphocytes, compared with males (41). It is also possible that the origin of the MDA-F471 cell line (a female *Gprc5a*^−/−^ mouse) may underlie the sex disparity observed with regards to growth of the spheres– though we found that the parental cell counterparts exhibited similar growth rates in male and female animals. Given the syngeneic setting of our experiments, it is reasonable to surmise that host immune responses may likely be implicated in the augmented growth of xenotransplanted CSCs relative to parental cells in female but not in male animals—a supposition that can be addressed in future studies.

We performed RNA-Seq profiling coupled with functional pathways and gene-gene interaction network analysis of CSCs and parental cell counterparts from *Gprc5a*^−/−^ *Kras*-mutant LUAD cells to interrogate molecular features that may underlie the biology of these CSCs. We identified 2,600 differentially expressed gene features that were further functionally analyzed and organized into canonical pathways and gene sets that were significantly modulated in CSCs. Subsequently, we validated a number of the differentially expressed genes by qRT-PCR. First and foremost, we demonstrated up-regulation of markers in CSCs from *Gprc5a*^−/−^ *Kras*-mutant LUAD that are widely known to be overexpressed in stem cells of different cancers (34). These included the three aldehyde dehydrogenases *Aldh1a1, Aldh1a3*, and *Aldh3a1*. We found that both G1 and G5 spheres, following flow cytometry analysis, exhibited significantly augmented pan-ALDH activity compared to parental cells and were thus more enriched with ALDH^+^ cells. Class 1 of the ALDH family is predominantly expressed in mammalian tissues, and its increased activity has been discovered in different CSCs including lung cancer (42). Specifically, ALDH^+^ lung CSCs were shown to generate tumors that mimic the heterogeneity of lung cancer cells *in vivo* and were associated with the aggressive phenotype and poor prognosis of human NSCLC (42). In addition to *ALDH1A1*, it is noteworthy that *ALDH3A1* was demonstrated to be overexpressed in LUAD compared to normal alveolar cells, the most likely cells of origin/stem cells of this tumor (11). Likewise, we confirmed the up-regulation of two previously characterized epithelial stem cell markers, *Epcam* and *Alcam*, and that have been previously shown to be over-expressed in LUAD CSCs (34). Our profiling efforts also showed up-regulation of previously identified drivers of stemness in the CSCs from *Gprc5a*^−/−^ *Kras*-mutant LUAD such as *Arrb1* and *Tgm2*. *Arrb1* was shown to play major roles in invasion, migration and epithelial-to-mesenchymal transition (EMT) of CSCs, thus underlying their metastatic potential (43). Additionally, a recent study showed that *Arrb1* is involved in the self-renewal and expansion of NSCLC CSCs (44). Similarly, the up-regulation of *Tgm2* was reported to promote an aggressive phenotype in CSCs characterized by increased survival, metastasis and drug resistance (45). Also, the *Gprc5a*^−/−^ *Kras*-mutant LUAD CSCs exhibited mRNA expression of the mucin-secreting gene *Muc5ac*. A recent report demonstrated that over-expression of *Muc5ac* was associated with the development of *Kras*-mutant LUADs *in vivo* and with poorer clinical outcome in human LUAD (26). Conversely, we found that *Wnt7a*, a component of the Wnt/β-catenin pathway (46) and the loss of which was previously reported to promote an undifferentiated tumor-protective phenotype in NSCLC (24), was markedly suppressed in the *Gprc5a*^−/−^ *Kras*-mutant LUAD CSCs relative to parental cells. These data suggest that our derived spheres from *Gprc5a*^−/−^ *Kras*-mutant LUAD cells exhibit pertinent and major molecular features of CSCs.

Our RNA-Seq and pathways analyses also unraveled additional cues and mechanisms that are significantly altered in the CSCs, namely augmented pro-tumor inflammatory signaling and anti-oxidant pathways. We found a number of cytokines that were up-regulated in the spheres/CSCs compared to parental cells, including *Cxcl10, Ccl20*, and *Tnf*. Previous studies have demonstrated that inflammatory responses and stimuli from immune cells play important roles in carcinogenesis and in inducing a stem cell phenotype (47). Inflammation has been shown to be a key factor in promoting tumorigenesis primarily through the NF-κB pathway, which is activated in response to proinflammatory cytokines such as TNF the increased levels of which are strongly associated with the aggressive phenotype of various cancers (48). Additionally, CCL20 and CXCL10 play major roles in the growth, migration and metastatic capabilities of lung cancer cells and were shown to decrease immunogenicity against cancer cells through recruitment of inflammatory immune cells, leading to decreased patient survival (49, 50). Conversely, we found that the *Gprc5a*^−/−^ *Kras*-mutant LUAD CSCs exhibited lower levels of Il-24. This cytokine was previously reported to exert anti-tumorigenic effects on lung cancer cells by inhibiting various mechanisms of tumor growth and metastasis concomitant with promotion of cell death (51). It is important to note that accumulating evidence suggests that the antioxidant pathway plays an important role in maintaining normal cellular functions under oxidative insults and increased production of reactive oxygen species (52, 53). The activation of different components of this pathway attenuates the oxidative stress-mediated cellular damage that is increased in many aggressive cancers contributing to their growth, survival and resistance to therapeutic drugs (53). Here, we found that the murine CSCs exhibited significantly up-regulated levels of two major genes in the NRF2 antioxidant pathway, *Atf3* and *Gstm1* suggesting that anti-oxidant capacity may be elevated in the *Gprc5a*^−/−^ *Kras*-mutant LUAD CSCs. It is important to note that our RNA-Seq profiling centered on cultured cells *in vitro*. We do not know, at the present, whether our findings would extend to the *in vivo* setting. Future studies profiling *Gprc5a*^−/−^ *Kras* mutant CSCs vs. parental cell xenotransplants may further aid in elucidating expression programs that embody an augmented malignant phenotype by CSCs, including expression cues associated with the host immune response and tumor microenvironment. Of note, we validated several markers of pro-tumor inflammatory and antioxidant signaling, along with ALDHs (see above), in spheres we had derived and cultured from human codon 12 *KRAS*-mutant LUAD cells (H1792 cells). It is thus plausible that expression programs we underscored in the *Gprc5a*^−/−^ *Kras*-mutant LUAD CSCs may be conserved, at least in part, in human *KRAS*-mutant LUAD; particularly programs influencing stemness, pro-tumor inflammation and antioxidant function.

Glycogen synthase kinase 3 beta (GSK3β) plays key roles in various cellular and physiological processes and exerts opposing effects depending on the type of cancer (54, 55). A growing body of evidence is shedding light on the tumor-promoting roles of GSK3β in diverse cancers, and a recent study has shown that it is involved in glioblastoma cancer stem cell self-renewal (56). Moreover, GSK3β has been associated with worse clinical outcomes in bladder cancer (57). In our present study, Gsk3β was found, *in silico*, to be further activated in MDA-F471 CSCs compared to parental cells. Also, we demonstrated that targeting this molecule using the irreversible GSK3β inhibitor tideglusib markedly inhibited the growth of *Gprc5a*^−/−^ *Kras*-mutant LUAD spheres and almost completely abolished their sphere forming capacity. Interestingly, the same doses of tideglusib exhibited considerably more subtle effects on the parental cell counterparts. Our findings suggest that GSK3β may be target for therapy of human *KRAS*-mutant LUAD. It is important to note that our results, at the present, are preliminary and hypothesis-generating as we evaluated the effects of this agent *in vitro* and primarily using one murine *Kras*-mutant LUAD cell line. Future studies are warranted to assess the effects of GSK3β inhibition on *Kras*-mutant LUAD CSC xenotransplants *in vivo*. Also it is not clear if these findings will extend to *in vivo* models with xenotransplanted human cells and whether GSK3β inhibition may represent a viable strategy for treatment of human *KRAS*-mutant LUAD. Nonetheless, we evaluated the effects of GSK3β inhibition by tideglusib in human H1792 spheres and found that targeting this kinase similarly inhibited sphere growth and formation of these human *KRAS*-mutant LUAD cells. Future studies are warranted to further scrutinize the therapeutic potential of GSK3β inhibition in human *KRAS*-mutant LUAD.

Our study is not without limitations. It cannot be neglected that we primarily interrogated one murine *Kras*-mutant LUAD cell line (and the *Gprc5a*^−/−^ model), the MDA-F471 line. Of note, this murine cell line was originally derived and cultured from a LUAD that had developed in a *Gprc5a*^−/−^ mouse that was exposed to tobacco carcinogen (14). Thus, it is reasonable to surmise that this cell line is perhaps relevant to understanding different features of *Kras*-mutant LUAD because the human counterpart primarily develops from a background of smoking (3). It is not clear how our findings (e.g., gene expression programs) extend to the CSC phenotype in human *KRAS*-mutant LUAD. Future studies are warranted to comparatively explore evolutionarily conserved expression programs in CSCs of murine (e.g., *Gprc5a*^−/−^) and human LUADs with somatic mutations in the *KRAS* oncogene. Also, and as mentioned above, our findings on the effects of GSK3β inhibition on CSCs/spheres from *Kras*-mutant LUAD cells are at this stage preliminary and the therapeutic potential of inhibiting this kinase on the human counterpart are still elusive. It is noteworthy, that we attempted to address these voids by using the human H1792 LUAD cell line for validation of our findings. We confirmed the differential expression of various genes, identified by RNA-Seq analysis of the murine MDA-F471 cell line, in the H1792 cells. Further, we validated the effects of tideglusib treatment and found that this GSK3β inhibitor similarly inhibited the growth and sphere forming ability of H1792 CSCs/spheres. It is worthwhile to mention that the H1792 cell line is a human *KRAS*-mutant (codon 12) LUAD cell line that was originally derived from a smoker patient (58, 59). Additionally, the H1792 cell line was shown to express very low levels of human *GPRC5A* (17). These features render the H1792 cell line suitable for validation of our findings stemming from the murine MDA-F471 cells which is also a *Kras*-mutant LUAD line that is derived from a tobacco-carcinogen exposed animal (14). Thus, although we validated some of our findings in only one human LUAD cell line, we suppose that these efforts are a step in the right direction toward probing the molecular and therapeutic relevance of our data to human *KRAS*-mutant LUAD. Another limitation in our study is the absence of functional validation for a direct target identified as differentially expressed by RNA-Seq analysis in the *Gprc5a*^−/−^ LUAD CSCs. Also, it is not clear whether any of the identified differentially expressed genes are linked to the GSK3β pathway and thus to the effects of inhibiting this kinase by tideglusib. Future studies are warranted that can further scrutinize the role of these putative CSC genes/markers in human *KRAS*-mutant LUAD as well as scrutinize the role of the GSK3β in the molecular CSC phenotype in the human malignancy.

All in all, we derived and cultured cells from murine *Gprc5a*^−/−^ *Kras*-mutant LUAD with stem cell-like characteristics. We demonstrated that these cells exhibited increased tumorigenesis in a syngeneic setting *in vivo*. We also identified by coupled RNA-Seq and pathways analysis differential expression programs in the murine CSCs that embody an augmented malignant phenotype and, thus, comprise high-potential therapeutic targets. As a proof of concept, we demonstrated marked anti-growth effects of an inhibitor of GSK3β, tideglusib, against these CSCs. Our findings shed new light on the molecular pathogenesis of LUAD with somatically acquired *Kras* mutations and pave the way for future studies for further interrogating the role of CSCs in the development of this fatal subtype of lung malignancy and agents that can target molecular cues in these cells.

## Data Availability

The datasets for this manuscript are not publicly available because the data will be deposited into GEO upon publication. Requests to access the datasets should be directed to hnkadara1@gmail.com.

## Author Contributions

WA-K and HK conceived the study and supervised the study. RD, MH, HB, and AS performed experiments and analyzed data. RD, WA-K, and HK wrote the first draft of the manuscript. All authors approved the final version of the manuscript.

### Conflict of Interest Statement

The authors declare that the research was conducted in the absence of any commercial or financial relationships that could be construed as a potential conflict of interest.
