# Supplementary material for: Genome-Wide and Phenotypic Evaluation of Stem Cell Progenitors Derived From Gprc5a-Deficient Murine Lung Adenocarcinoma With Somatic Kras Mutations
Source: Front Oncol. 2019 Apr 2;9:207. doi: 10.3389/fonc.2019.00207 (PMC6454871; doi:10.3389/fonc.2019.00207)
Supplement: Supplementary file 1 [file Data_Sheet_1.PDF]

Genome-Wide and Phenotypic Evaluation of Stem Cell Progenitors Derived from *Gprc5a*-deficient Murine Lung Adenocarcinoma with Somatic *Kras* Mutations

Reem Daouk, Maya Hassane, Hisham Bahmad, Ansam Sinjab, Junya Fujimoto, Wassim Abou-Kheir, Humam Kadara

SUPPLEMENTARY TABLES

Table S1. Primer sequences and annealing temperatures for select murine genes.

Murine gene	Primer sequence (5' – 3')	Annealing temp. (°C)	Reference
Alcam	F- ATGGCATCTAAGGTGTCCCCT<br>R- AGACGGCAAGGCATGACAA	60	[1]
Aldh1a1	F- GACAGGCTTTCCAGATTGGCTC<br>R- AAGACTTTCCCACCATTGAGTGC	60	[2]
Aldh1a3	F- AGGCTGTATTAAGACCTTCAG<br>R- GGAAGTTCCATGGTGTAATG	55	[3]
Aldh3a1	F- GATGCCCATTGTGTGTGTTCG<br>R- CCACCGCTTGATGTCTCTGC	60	[2]
Arrb1	F- CCGAGGACAAGAAGCCACTGA<br>R- AGAGTGACTGAGCATGGAAGGT	57	[4]
Atf3	F- GAGGATTTTGCTAACCTGACACC<br>R- TTGACGGTAACTGACTCCAGC	57	[5]
Ccl20	F- GTGGGTTTCACAAGACAGATG<br>R- TTTTCACCCAGTTCTGCTTTG	57	[6]
Cxcl10	F- GCTGCCGTCATTTTCTGC<br>R- TCTCACTGGCCCGTCATC	57	[7]
Epcam	F- AGGGGCGATCCAGAACAACG<br>R- ATGGTCGTAGGGGCTTTCTC	57	[8]
Gapdh	F- GCAAAGTGGAGATTGTTGCCA<br>R- GCCTTGACTGTGCCGTTGA	60	[9]
Gstm1	F- CCTGGATGGAGAGACAGAGG<br>R- GACCTTGTCCCCTGCAAA	55	[10]
Il24	F- GCCCAGTAAGGACAATTCCA<br>R- ATTTCTGCATCCAGGTCAGG	60	[11]
Muc5ac	F- TCTACCACTCCCTGCTTCT<br>R- TGACTAACCCTCTTGACCAC	55	[12]
Tbp	F- CCTTGTACCCTTCACCAATGAC<br>R- ACAGCCAAGATTCACGGTAGA	60	[13]
Tgm2	F- TTCCGGCTGACTCTGTACTTCGAG<br>R- ACATTGTCCTGTTGGTCCAGCACT	61.3	[14]
Tnf	F- TCAGCCGATTTGCTATCTCATA<br>R- AGTACTTGGGCAGATTGACCTC	57	[15]
Wnt7a	F- CGACTGTGGCTGCGACAAG<br>R- CTTCATGTTCTCCTCCAGGATCTTC	60	[16]

Table S2. Primer sequences and annealing temperatures for select human genes.

Human gene	Primer sequence (5' – 3')	Annealing temp. (°C)	Reference
ALDH1A1	F- TGTTAGCTGATGCCGACTTG	60	[17]
	R- TTCTTAGCCCGCTCAACACT		
ALDH3A1	F- GCAGACCTGCACAAGAATGA	60	[18]
	R- TGTAGAGCTCGTCCTGCTGA		
ATF3	F- CTCTGCGCTGGAATCAGTCA	60	[19]
	R- TCGCCTCTTTTTCCTTTCATCT		
CCL20	F- GGTGAAATATATTGTGCGTCTCC	60	[20]
	R- ACTAAACCCTCCATGATGTGC		
GAPDH	F- GTCAGTGGTGGACCTGACCT	60	[21]
	R- TCGCTGTTGAAGTCAGAGGA		
IL24	F- GACTTTAGCCAGCAGACCCTT	55	[22]
	R- GGTTGCAGTTGTGACACGAT		
TBP	F- TTGGGTTTTCCAGCTAAGTTCT	60	[23]
	R- CCAGGAAATAACTCTGGCTCA		
TNF	F- ACTTTGGAGTGATCGGCC	60	[20]
	R- GCTTGAGGGTTTGCTACAAC		

Table S3. Tumor numbers evaluated at thirty days post-injection of MDA-F471 G1 spheres and parental cells.

		Number of tumors at end point		
**Cell number**	**Cell type**	**Males**	**Females**	**Total**
**500,000**	G1	6/6	6/6	12/12
	Parental	6/6	6/6	12/12
**10,000**	G1	4/6	4/5	8/11
	Parental	1/6	3/6	4/12
**1,000**	G1	3/6	4/6	7/12
	Parental	3/6	1/6	4/12

Table S4. The number of tumors evaluated at thirty days post injection of MDA-F471 G5 spheres and parental cells.

		Number of tumors at end point		
**Cell number**	**Cell type**	**Males**	**Females**	**Total**
**10,000**	G5	4/6	6/6	10/12
	Parental	5/6	3/6	8/12
**1,000**	G5	2/5	5/6	7/11
	Parental	4/6	0/6	4/12

Table S5. List of all differentially expressed genes between MDA-F471 G1 spheres and parental cells.

Transcript	Gene symbol	Fold-change (log base 2)
NM_017474	*Clca1*	11.5228
NM_010582	*Itih2*	11.2282
NM_011339	*Cxcl15*	10.5524
NM_028801	*Muc5b*	10.3071
NM_001254761	*Rnf128*	9.4605
NM_022882	*Lpin2*	9.2723
NM_007752	*Cp*	9.2719
NM_133675	*Sptssb*	9.0339
NM_017370	*Hp*	9.0156
NM_001164369	*Bcas1*	8.8258
NM_133977	*Trf*	8.8007
NM_009727	*Atp8a1*	8.7411
NM_001346518	*5330417C22Rik*	8.6449
NM_025467	*Gkn2*	8.6359
NM_009160	*Sftpd*	8.6022
NR_102366	*AW112010*	8.5891
NM_010518	*Igfbp5*	8.3921
NM_028021	*Myh14*	8.2643
NM_146050	*Oit1*	8.1563
NM_133867	*Eps8l3*	8.1105
NM_001271725	*Trim2*	8.0554
NM_009509	*Vil1*	7.9145
NM_008630	*Mt2*	7.8068
NM_009778	*C3*	7.6593
NM_080457	*Muc4*	7.5904
NM_010706	*Lgals4*	7.5192
NM_010844	*Muc5ac*	7.5031
NM_175454	*Hid1*	7.4681
NM_008362	*Il1r1*	7.4272
NM_021274	*Cxcl10*	7.4177
NM_172881	*Ugt2b35*	7.4081
NM_027902	*Tmprss6*	7.3985
NM_008198	*Cfb*	7.2593
NM_019972	*Sort1*	7.2584
NM_001351947	*Olfm4*	7.2424
NM_001159738	*Ccl20*	7.222
NM_181579	*Pof1b*	7.179
NM_030181	*Vsig1*	7.1521
NM_203320	*Cxcl3*	7.1476
NM_001001495	*Tnip3*	7.0841
NM_130887	*Papln*	7.0759
NM_025714	*Odf2l*	7.0519
NM_009140	*Cxcl2*	7.0251
NM_001166580	*8430408G22Rik*	6.9867
NM_028064	*Slc39a4*	6.9338
NM_008489	*Lbp*	6.928
NM_001289584	*Sytl2*	6.9019
NM_020043	*Igdcc4*	6.8706
NM_145980	*8430408G22Rik*	6.868
NM_172646	*Dnm3*	6.7702

NM_023478	*Upk3a*	6.7626
NM_013697	*Ttr*	6.7522
NM_011998	*Chst4*	6.7271
NM_001286049	*Entpd5*	6.7058
NM_001037937	*Deptor*	6.7018
NM_011463	*Spink4*	6.6939
NM_028807	*Exoc3l4*	6.6595
NM_013693	*Tnf*	6.6593
NM_016745	*Atp2a3*	6.6545
NM_011979	*Vnn3*	6.6015
NM_009141	*Cxcl5*	6.573
NM_008256	*Hmgcs2*	6.52
NM_029815	*Bcas1*	6.5084
NM_001164210	*Sptssb*	6.4886
NM_026935	*Sult1c2*	6.4779
NM_001277944	*Apoc2*	6.4732
NM_139198	*Plac8*	6.4608
NM_134072	*Akr1c14*	6.4056
NM_178767	*Agmo*	6.3702
NM_001164497	*Papd5*	6.3563
NM_010197	*Fgf1*	6.3448
NM_013832	*Rasal1*	6.3081
NM_016960	*Ccl20*	6.2754
NR_104580	*Slc8a1*	6.2653
NM_001313980	*Eif4e*	6.2417
NM_001276481	*Dag1*	6.2145
NM_001040682	*Clmn*	6.1838
NM_009692	*Apoa1*	6.1792
NM_011468	*Sprr2a1*	6.1773
NM_080728	*Myh7*	6.1503
NM_010390	*H2-Q1*	6.0887
NM_011575	*Tff3*	6.0845
NM_146240	*Rassf9*	6.0763
NM_001161838	*Ptprr*	6.0317
NM_080575	*Acss1*	6.0218
NM_134110	*Kcne2*	6.0104
NM_027853	*Mettl7b*	5.9848
NM_011468_dup1	*Sprr2a1*	5.9695
NM_001287800	*Fhl1*	5.9313
NM_001291119	*Neurl4*	5.9311
NM_153576	*Cxcl17*	5.9215
NM_001033122	*Cd69*	5.9186
NM_146136	*Slc16a4*	5.9158
NM_001013785	*Akr1c19*	5.912
NM_011366	*Sorbs3*	5.906
NM_153778	*Atoh8*	5.8594
NM_175638	*Wnk4*	5.8577
NM_022317	*Slc28a3*	5.828
NM_009693	*Apob*	5.8098
NM_010271	*Gpd1*	5.7701
NR_033217	*BC030870*	5.7506
NM_001190352	*Tmem132b*	5.7013
NM_001311140	*Bmf*	5.6982
NM_207663	*Synm*	5.67
NM_001033481	*Myrf*	5.6695

NM_029782	*Calr3*	5.6585
NM_007914	*Ehf*	5.6344
NM_177743	*Fam198a*	5.6291
NM_009888	*Cfh*	5.6282
NM_021411	*Rab37*	5.616
NM_020622	*Fam3b*	5.6123
NM_020574	*Kcne3*	5.6021
NM_011821	*Gpc6*	5.5962
NM_001309382	*Sprr2a3*	5.5749
NM_178594	*Vtcn1*	5.533
NM_001110517	*Ifit1bl1*	5.5273
NM_013467	*Aldh1a1*	5.5146
NM_177026	*Tmcc3*	5.5114
NM_025622	*Lgals2*	5.4954
NM_001159556	*Cd36*	5.4848
NM_008124	*Gjb1*	5.4804
NM_001159505	*Tnfsf13*	5.4672
NM_030601	*Clca3a2*	5.4608
NM_153598	*Ugt2b34*	5.4434
NM_201410	*Ugt1a6b*	5.4338
NM_010936	*Nr1i2*	5.4303
NM_178727	*D630039A03Rik*	5.4249
NM_207270	*Ptprh*	5.4074
NM_023517	*Tnfsf13*	5.3725
NM_001164566	*Spats2l*	5.3505
NM_028757	*Nebl*	5.341
NM_001033713	*Mef2a*	5.3393
NM_001161406	*Grhl1*	5.3203
NM_001316740	*Fam213a*	5.3132
NM_138951	*Ttc36*	5.3054
NM_009738	*Bche*	5.299
NM_001164499	*Papd5*	5.2982
NM_001276250	*Cp*	5.2977
NM_011921	*Aldh1a7*	5.2964
NM_145079	*Ugt1a6a*	5.2928
NR_028405	*Marcksl1-ps4*	5.2866
NM_001289493	*Gbp3*	5.2698
NM_007646	*Cd38*	5.2567
NM_001252435	*Dlg1*	5.2494
NM_145373	*Sectm1a*	5.2417
NM_001164173	*Cpsf1*	5.2155
NM_181748	*Ffar4*	5.2072
NM_026385	*Pllp*	5.1657
NM_010602	*Kcnj11*	5.1651
NM_010707	*Lgals6*	5.1604
NM_001105252	*Tmc5*	5.152
NM_009754	*Bcl2l11*	5.1494
NM_008609	*Mmp15*	5.133
NM_001159344	*Casz1*	5.1266
NM_001042613	*Sepp1*	5.1188
NM_054037	*Scgb3a1*	5.1133
NM_001347084	*Ahcyl2*	5.0944
NM_183160	*Tmem252*	5.0891
NM_007807	*Cybb*	5.0747
NM_001159986	*Sec16b*	5.0435

NM_007817	*Cyp2f2*	5.0259
NM_001282095	*Tjp3*	5.0164
NM_001033170	*Fam83e*	5.0072
NM_181595	*Ppp1r9a*	5.0053
NM_054098	*Steap4*	4.9993
NM_001313938	*Proc*	4.977
NM_027299	*Degs2*	4.9748
NM_001163217	*Fgfr3*	4.9633
NM_001331221	*Bmf*	4.9527
NM_001097617	*C1s1*	4.9356
NM_018805	*Hs3st3b1*	4.9316
NM_023395	*Wfdc1*	4.9173
NM_001033304	*5330417C22Rik*	4.8929
NM_013769	*Tjp3*	4.8865
NM_023755	*Tfcp2l1*	4.8771
NM_001162476	*Stra6*	4.8766
NM_010359	*Gstm3*	4.8749
NM_053080	*Aldh1a3*	4.8735
NM_194336	*Gbp6*	4.8548
NM_001013779	*Aim2*	4.8517
NM_013724	*Nrk*	4.8487
NM_027468	*Cpm*	4.8443
NM_008518	*Ltb*	4.8365
NM_007860	*Dio1*	4.8191
NM_011408	*Slfn2*	4.8154
NM_025778	*Bcl2l14*	4.8062
NM_008370	*Il5ra*	4.8052
NM_001170800	*Ipcef1*	4.8029
NM_028085	*Anks4b*	4.7952
NM_001081328	*Chsy3*	4.7696
NM_028191	*Cyp2c65*	4.7488
NM_172778	*Maob*	4.748
NM_007548	*Prdm1*	4.741
NM_146026	*Prr15l*	4.7389
NR_102292	*Rpl22*	4.7295
NM_001012434	*Kctd14*	4.7164
NM_011704	*Vnn1*	4.7101
NM_022312	*Tnr*	4.7062
NM_207031	*Ano7*	4.6853
NM_145403	*Tmprss4*	4.6787
NM_001081981	*Nfix*	4.6745
NM_009579	*Slc30a1*	4.667
NM_172854	*Olfml2a*	4.6383
NM_001290315	*Igdcc4*	4.6366
NM_001083312	*Gbp7*	4.6295
NM_175032	*Galntl6*	4.6136
NM_010356	*Gsta3*	4.6058
NM_009731	*Akr1b7*	4.6028
NM_008764	*Tnfrsf11b*	4.5856
NM_007609	*Casp4*	4.5796
NM_001290506	*Mid1*	4.5684
NM_001286058	*Entpd5*	4.5673
NM_001199927	*Fam198a*	4.5491
NM_001039678	*Urad*	4.5407
NM_133670	*Sult1a1*	4.5267

NM_028096	*2010300C02Rik*	4.5178
NM_010406	*Hc*	4.5075
NM_009150	*Selenbp1*	4.4999
NM_029554	*0610040J01Rik*	4.4941
NM_001136235	*Kctd14*	4.4547
NM_008281	*Hpn*	4.4388
NM_001005341	*Ypel2*	4.426
NM_029931	*Mllt3*	4.4101
NM_008491	*Lcn2*	4.3968
NM_018732	*Scn3a*	4.3876
NM_007823	*Cyp4b1*	4.378
NM_001145806	*Capn8*	4.3751
NM_001080812	*Cib3*	4.3727
NM_027918	*1300017J02Rik*	4.3592
NM_001190448	*Ddc*	4.351
NM_001271873	*Ano10*	4.3446
NM_001110252	*Hpn*	4.3429
NM_010742	*Ly6d*	4.3397
NM_009327	*Hnf1a*	4.3348
NM_019517	*Bace2*	4.3197
NM_009264	*Sprr1a*	4.282
NM_015790	*Icosl*	4.2792
NM_145447	*Mfsd7c*	4.2759
NM_133681	*Tspan1*	4.2757
NM_133775	*Il33*	4.2682
NM_019414	*Selenbp2*	4.2611
NM_010358	*Gstm1*	4.2507
NM_175485	*Prtg*	4.2489
NM_001033141	*Ecscr*	4.2457
NM_001256309	*Gm933*	4.2427
NM_001033290	*Gpr55*	4.2324
NM_001162998	*Smim6*	4.2154
NM_010060	*Dnah11*	4.2142
NM_017371	*Hpx*	4.2039
NM_182839	*Tppp*	4.1938
NM_177371	*Tnfsf15*	4.1914
NM_015775	*Tmprss2*	4.1817
NM_008475	*Krt4*	4.1712
NM_144899	*Adamtsl4*	4.1702
NM_001145644	*Phgr1*	4.1698
NM_028902	*Hsf2bp*	4.1681
NM_008176	*Cxcl1*	4.1646
NM_138313	*Bmf*	4.1619
NR_003508	*Mx2*	4.1606
NM_023617	*Aox3*	4.1551
NR_130173	*C230014O12Rik*	4.148
NM_001077508	*Tnfrsf9*	4.1381
NM_009626	*Adh7*	4.1344
NM_008449	*Kif5c*	4.123
NM_001146294	*Celf4*	4.1191
NM_007428	*Agt*	4.1119
NM_025359	*Tspan13*	4.1105
NM_080455	*Tshz2*	4.1054
NM_010357	*Gsta4*	4.0942
NM_008557	*Fxyd3*	4.0921

NM_007885	*Slc26a2*	4.0761
NM_001291891	*Adam19*	4.0643
NM_134102	*Pla1a*	4.0363
NM_175314	*Adamts9*	4.0311
NM_001276248	*Cp*	4.0244
NM_007643	*Cd36*	4.0147
NM_011345	*Sele*	4.0135
NM_201642	*Ugt1a7c*	4.0039
NM_012006	*Acot1*	4.003
NM_016675	*Cldn2*	4.0028
NM_172399	*Ndnf*	3.9937
NM_001081756	*Nckap5*	3.9916
NM_008260	*Foxa3*	3.9815
NM_172469	*Clic6*	3.9806
NM_001159557	*Cd36*	3.9788
NM_022430	*Ms4a8a*	3.9751
NM_027997	*Serpina9*	3.9743
NM_027270	*Exoc1*	3.9668
NM_001161769	*Lmo4*	3.9646
NM_001205343	*Papln*	3.9576
NM_013602	*Mt1*	3.9544
NM_009363	*Tff2*	3.945
NM_009763	*Bst1*	3.9416
NM_176846	*Exph5*	3.9372
NM_010821	*Mpeg1*	3.936
NM_001347367	*Adgrl3*	3.9264
NM_001127259	*Trp63*	3.9225
NM_001173553	*Cdc14a*	3.9195
NM_001177670	*Golph3l*	3.8924
NM_008432	*Kcnu1*	3.8844
NM_001301295	*Cidec*	3.8829
NM_018734	*Gbp3*	3.8769
NM_173047	*Cbr3*	3.8547
NM_001293694	*Cept1*	3.8533
NM_153527	*Dnajb13*	3.8496
NM_001164724	*Il33*	3.8336
NM_146008	*Tcp11l2*	3.8258
NM_001077353	*Gsta3*	3.8218
NM_026328	*Reg4*	3.8198
NM_146017	*Gabrp*	3.8194
NM_011987	*Pla2g10*	3.8057
NM_008217	*Has3*	3.8011
NM_011212	*Ptpre*	3.8007
NM_009402	*Pglyrp1*	3.7984
NM_007555	*Bmp5*	3.7929
NM_011579	*Tgtp1*	3.7696
NM_001082553	*Rab27b*	3.7511
NM_009362	*Tff1*	3.7477
NM_028775	*Cyp2s1*	3.746
NM_017464	*Nedd9*	3.7356
NM_008607	*Mmp13*	3.7216
NM_028882	*Sema3d*	3.7167
NM_029360	*Tm4sf5*	3.7114
NM_026907	*Sectm1b*	3.7011
NM_001122603	*Fcgbp*	3.6849

NM_001288617	*Gsta3*	3.6737
NM_138674	*Pkhd1l1*	3.6684
NM_001277312	*Mea1*	3.6622
NM_001029867	*Ugt2b36*	3.6574
NM_053099	*Setbp1*	3.6465
NM_007576	*C4bp*	3.6454
NM_172835	*Peli3*	3.6423
NM_054041	*Antxr1*	3.6326
NM_008181	*Gsta1*	3.6324
NM_153782	*Fam20a*	3.6321
NM_001044740	*Slc7a2*	3.6303
NM_010260	*Gbp2*	3.6257
NM_001290764	*Pnkp*	3.621
NM_011026	*P2rx4*	3.6049
NM_007656	*Cd82*	3.5942
NM_001271599	*Sort1*	3.5837
NM_175164	*Arhgap26*	3.5782
NM_001177841	*Otub2*	3.576
NM_001001322	*Adamts13*	3.5745
NM_001111143	*Cym*	3.5727
NM_177192	*Dennd5b*	3.5618
NM_001040699	*Mtmr7*	3.5593
NM_153054	*Slc18a1*	3.5503
NM_011741	*Zan*	3.546
NM_001104617	*Rdx*	3.5451
NM_145394	*Slc44a3*	3.5389
NM_001316748	*Tspan14*	3.5343
NM_001145164	*Tgtp2*	3.5212
NM_010259	*Gbp2b*	3.4893
NM_030218	*Misp*	3.4857
NM_001136089	*Anxa10*	3.4697
NM_031884	*Abcg5*	3.4605
NM_001281999	*Rasal1*	3.4523
NM_001083904	*Fetub*	3.4504
NM_144879	*Vash2*	3.4496
NM_172476	*Tmc7*	3.4472
NM_001302163	*Acsl1*	3.4364
NM_181344	*C1rl*	3.4295
NM_133903	*Spon2*	3.4261
NM_001316736	*Fam213a*	3.4256
NM_018876	*Fut2*	3.4187
NM_010401	*Hal*	3.4083
NM_053109	*Clec2d*	3.4068
NM_009691	*Aplp2*	3.4037
NM_013492	*Clu*	3.4032
NM_001163316	*Get4*	3.4029
NM_001033367	*Nlrc4*	3.3993
NM_010234	*Fos*	3.3954
NM_001329965	*Hp*	3.3882
NM_016846	*Rgl1*	3.3873
NM_011041	*Pax9*	3.364
NM_001243092	*Gm3776*	3.3634
NM_021367	*Tslp*	3.3581
NM_001122660	*Gm10639*	3.3568
NR_027618	*Vps39*	3.356

NM_007799	*Ctse*	3.3529
NM_001271540	*Myh14*	3.3476
NM_018778	*Cldn8*	3.3472
NM_028182	*Sh2d4a*	3.3435
NM_010474	*Hs3st1*	3.3412
NM_027908	*Amdhd1*	3.3295
NM_001130515	*Hexim2*	3.3143
NM_023719	*Txnip*	3.314
NM_008172	*Grin2d*	3.3003
NM_001145015	*Arhgap40*	3.3001
NM_001347153	*Krba1*	3.2977
NM_001160268	*Plekha6*	3.2888
NM_001167937	*D6Ertd527e*	3.2872
NM_011510	*Abcc8*	3.285
NM_011139	*Pou2f3*	3.2826
NM_031167	*Il1rn*	3.2805
NM_019483	*Smad9*	3.2771
NM_201639	*Synm*	3.2745
NM_027077	*1700016C15Rik*	3.2716
NM_009421	*Traf1*	3.2679
NR_110955	*Tmem67*	3.2677
NR_027710	*Ppargc1a*	3.2672
NM_207237	*Man1c1*	3.2666
NM_177632	*Fam43a*	3.2634
NM_001034863	*Tmem136*	3.257
NM_030075	*Klhdc8b*	3.2518
NM_001271461	*Cd82*	3.2481
NM_025807	*Slc16a9*	3.2478
NM_001282032	*Hax1*	3.2455
NM_025330	*Hsd17b14*	3.2419
NM_010189	*Fcgrt*	3.2408
NM_021491	*Smpd3*	3.2401
NM_028618	*Dmkn*	3.2398
NM_134005	*Enpp3*	3.2349
NM_133898	*N4bp2l1*	3.2348
NM_172658	*Slco4c1*	3.2342
NM_178929	*Kazald1*	3.2262
NM_001253680	*Slc7a7*	3.2225
NM_001110337	*Gprc5c*	3.2219
NM_001287172	*Alpl*	3.2082
NM_011905	*Tlr2*	3.1907
NM_001162919	*Pgbd1*	3.1868
NM_008239	*Foxq1*	3.1867
NM_001033228	*Itga1*	3.1762
NM_175367	*Ston2*	3.1743
NM_201645	*Ugt1a1*	3.1727
NM_001311790	*Ctsc*	3.1685
NM_001159485	*Mcf2l*	3.1673
NM_009524	*Wnt5a*	3.1648
NM_001271542	*Cnot7*	3.1605
NM_011150	*Lgals3bp*	3.1597
NM_011082	*Pigr*	3.1427
NM_053085	*Tcf23*	3.1392
NM_138665	*Sardh*	3.1289
NM_009425	*Tnfsf10*	3.1225

NM_001164565	*Acnat1*	3.1201
NM_001104616	*Rdx*	3.1179
NM_177756	*Colgalt2*	3.1139
NM_001282947	*Apaf1*	3.1115
NM_172838	*Slc16a12*	3.1108
NM_027878	*Dram1*	3.108
NR_073583	*Ppp2r2b*	3.1045
NM_146187	*Ffar2*	3.0863
NM_001038999	*Atp8a1*	3.0846
NM_007621	*Cbr2*	3.078
NM_029000	*Gvin1*	3.0773
NM_027711	*Iqgap2*	3.0752
NM_028110	*Dennd2d*	3.0748
NM_199057	*Rusc2*	3.0731
NM_172884	*2900026A02Rik*	3.072
NM_010907	*Nfkbia*	3.0662
NM_001276413	*Fn1*	3.0569
NM_001289583	*Sytl2*	3.0538
NM_170680	*Abcc10*	3.046
NM_001253392	*Smarcad1*	3.0353
NM_007553	*Bmp2*	3.0277
NM_001347225	*Ipcef1*	3.0256
NM_027526	*Rasgef1a*	3.0201
NM_010846	*Mx1*	3.0105
NM_146064	*Soat2*	3.0101
NM_019744	*Ncoa4*	3.0055
NM_001033382	*Cacna2d4*	2.9988
NM_177657	*D630003M21Rik*	2.997
NM_025404	*Arl4d*	2.9953
NM_023557	*Slc44a4*	2.9936
NM_172439	*Inpp5j*	2.9925
NM_033373	*Krt23*	2.991
NM_009155	*Sepp1*	2.9883
NM_001033210	*Pls1*	2.986
NM_001347371	*Adgrl3*	2.9838
NM_007678	*Cebpa*	2.9798
NM_198942	*Dhx57*	2.9712
NM_009373	*Tgm2*	2.9686
NM_010305	*Gnai1*	2.9582
NM_009894	*Cideb*	2.9577
NM_175930	*Rapgef5*	2.9551
NM_147219	*Abca5*	2.9544
NM_001168512	*Ffar2*	2.9506
NM_008830	*Abcb4*	2.9494
NM_012050	*Omd*	2.9478
NM_001039555	*Cyp2c68*	2.9442
NM_027220	*Prss32*	2.9377
NM_008585	*Mep1a*	2.9327
NM_010231	*Fmo1*	2.932
NM_011333	*Ccl2*	2.9277
NM_001001326	*St5*	2.9232
NM_177340	*Synpo*	2.9141
NM_170727	*Scgb3a1*	2.9084
NM_010074	*Dpp4*	2.9057
NM_010118	*Egr2*	2.8965

NM_001252450	*Cldnd1*	2.8963
NM_027286	*Ace2*	2.8901
NM_001201470	*Papss2*	2.8857
NM_001271544	*Slc4a9*	2.8848
NM_001324530	*Nck1*	2.8846
NM_001310448	*Cdk14*	2.8846
NM_139300	*Mylk*	2.8804
NM_007426	*Angpt2*	2.8743
NM_153564	*Gbp5*	2.8742
NM_001110829	*Rbfox2*	2.867
NM_011338	*Ccl9*	2.8662
NM_001283029	*Spin1*	2.8647
NM_015749	*Tcn2*	2.8636
NM_177388	*Slc41a2*	2.8467
NM_001199284	*Slc43a2*	2.8464
NM_011976	*Sema4g*	2.8425
NM_180962	*Cyhr1*	2.8364
NM_008223	*Serpind1*	2.8362
NM_019946	*Mgst1*	2.8325
NM_146034	*Ctage5*	2.8153
NR_130973	*H19*	2.8145
NM_009801	*Car2*	2.8132
NM_001164199	*Prkacb*	2.8066
NM_001004173	*Sgpp2*	2.8046
NM_008635	*Map7*	2.8033
NM_001347164	*Atp6v0e2*	2.7994
NR_038116	*Cxcl11*	2.7944
NM_138628	*Txlnb*	2.7906
NM_013658	*Sema4a*	2.7885
NM_177920	*Serpina7*	2.7875
NM_001009935	*Txnip*	2.7846
NM_001329935	*Habp2*	2.7832
NM_001025263	*Tpd52*	2.7779
NM_001146215	*Msh5*	2.7762
NM_001242427	*Slc37a1*	2.7727
NR_003520	*Mx1*	2.7672
NM_026671	*Lypd2*	2.766
NM_001271578	*Sec1*	2.7621
NM_001290457	*Relb*	2.7614
NM_007746	*Map3k8*	2.7588
NM_011281	*Rorc*	2.7561
NM_177353	*Slc9a7*	2.7524
NM_001164488	*Wars*	2.7449
NM_021564	*Fetub*	2.741
NM_130890	*Capn8*	2.7367
NM_001291865	*Sat1*	2.7362
NM_053155	*Clmn*	2.7341
NM_001142706	*Cfb*	2.734
NM_194066	*Ifi27*	2.7315
NM_001081295	*Arhgef26*	2.7307
NM_019802	*Ggcx*	2.7296
NM_177462	*Zmym6*	2.7263
NM_001144993	*2810459M11Rik*	2.726
NM_177667	*Ttc22*	2.7204
NM_008965	*Ptger4*	2.72

NM_013851	*Abca8b*	2.7184
NM_026862	*Cd177*	2.7182
NM_019467	*Aif1*	2.7152
NM_026821	*Lurap1l*	2.7118
NM_001159419	*Sidt1*	2.7114
NM_030220	*Sp2*	2.7094
NM_001081219	*Myo1a*	2.7062
NM_007651	*Cd53*	2.7059
NM_001205053	*Jdp2*	2.7057
NM_007511	*Atp7b*	2.7038
NM_175437	*Gsap*	2.7022
NM_009665	*Amd1*	2.702
NM_001163557	*Ppfibp2*	2.6972
NM_001331215	*Rap1gap*	2.6936
NM_194268	*Onecut2*	2.6911
NM_028802	*Gpcpd1*	2.6906
NM_053115	*Acox2*	2.6858
NM_027843	*Arl14*	2.6753
NM_001164787_dup1	*Sprr2a2*	2.6717
NM_001101605	*Ifit1bl1*	2.6687
NM_177736	*Lrrc61*	2.6669
NR_074089	*Wwp1*	2.6655
NM_007792	*Csrp2*	2.6652
NM_133776	*Adgrf1*	2.6628
NM_008338	*Ifngr2*	2.6599
NM_008878	*Serpinf2*	2.6557
NM_001301781	*Ptpdc1*	2.6551
NM_009324	*Tbx2*	2.6504
NM_001293728	*Tuft1*	2.6455
NM_001033364	*Cdhr2*	2.6437
NM_001099331	*R3hdml*	2.6387
NM_008963	*Ptgds*	2.6378
NM_016771	*Sult1d1*	2.6353
NM_001109975	*Synpo*	2.6352
NM_009400	*Tnfrsf18*	2.6338
NM_178663	*Bend7*	2.6315
NM_008360	*Il18*	2.6297
NM_181849	*Fgb*	2.6288
NM_009044	*Rel*	2.6276
NM_001164062	*Stat5a*	2.6229
NM_001277316	*Fam134b*	2.6215
NM_010735	*Lta*	2.6191
NM_020279	*Ccl28*	2.614
NM_010753	*Mxd4*	2.6091
NM_001112725	*Aldh3a1*	2.6067
NM_177161	*P4ha3*	2.6055
NM_007977	*F8*	2.6043
NM_028829	*Paqr8*	2.6023
NM_133667	*Pdk2*	2.6004
NM_007482	*Arg1*	2.6
NM_008102	*Gch1*	2.5992
NM_001286844	*Etv3*	2.599
NM_001312918	*Ephx1*	2.5963
NM_016857	*Exoc7*	2.593
NM_001039546	*Myo6*	2.5921

NM_031158	*Ank1*	2.5897
NM_172671	*Lgr4*	2.5862
NR_121656	*Rorc*	2.585
NM_027990	*Lypd6b*	2.5838
NM_001177503	*Plekhd1*	2.5828
NM_001329619	*Enpp5*	2.5767
NM_001346609	*Scamp1*	2.5662
NM_001310483	*Lmbrd1*	2.5662
NM_207208	*Clca4a*	2.5616
NM_001291050	*Gpcpd1*	2.56
NR_131964	*Panct2*	2.5595
NM_001085509	*Myom3*	2.5537
NM_010870	*Naip5*	2.548
NM_008690	*Nfkbie*	2.5415
NM_027096	*Gpcpd1*	2.541
NM_001297554	*Apela*	2.5393
NM_008398	*Itga7*	2.5364
NM_001302440	*Zfp422*	2.5324
NM_007409	*Adh1*	2.5304
NM_001284412	*Vps25*	2.5253
NM_133904	*Acacb*	2.5189
NM_001271455	*Tnip1*	2.5173
NM_025856	*Sh3d21*	2.5172
NM_001042672	*Gpcpd1*	2.5167
NM_001172561	*Sphk2*	2.5132
NM_001302479	*Acy3*	2.5111
NM_133217	*Bco2*	2.51
NM_028500	*Calr3*	2.5052
NM_008036	*Fosb*	2.5045
NM_009523	*Wnt4*	2.5043
NM_009744	*Bcl6*	2.5038
NM_001033430	*Kdm7a*	2.5009
NM_008182	*Gsta2*	2.5003
NM_001304832	*Maff*	2.4984
NM_001001792	*Zfp239*	2.4966
NM_009841	*Cd14*	2.4944
NM_021359	*Itgb6*	2.4927
NM_001290308	*Col12a1*	2.4913
NM_018881	*Fmo2*	2.491
NM_010795	*Mgat3*	2.4887
NM_010496	*Id2*	2.4885
NM_029803	*Ifi27l2a*	2.4871
NM_008185	*Gstt1*	2.4851
NM_008278	*Hpgd*	2.4846
NM_033606	*Dqx1*	2.484
NM_026301	*Rnf125*	2.4831
NM_001302798	*Rab27b*	2.4807
NM_001033460	*Drc1*	2.4806
NM_009928	*Col15a1*	2.479
NM_001305882	*Mro*	2.4774
NM_001013780	*Slc25a34*	2.4764
NM_011990	*Slc7a11*	2.4734
NM_172621	*Clic5*	2.4712
NM_007981	*Acsl1*	2.4701
NM_007976	*F5*	2.4687

NM_001163155	*Col4a5*	2.4681
NM_146126	*Sord*	2.468
NM_138312	*Fam172a*	2.464
NM_007980	*Fabp2*	2.4632
NM_030565	*Fam20c*	2.462
NM_001166065	*Gcnt4*	2.4613
NR_033220	*BC039771*	2.46
NM_146125	*Itpka*	2.4589
NM_009437	*Tst*	2.4587
NM_001252447	*Vrk2*	2.4587
NM_023143	*C1ra*	2.4558
NM_145562	*Parm1*	2.4546
NM_001037712	*Kcnh6*	2.454
NM_001163776	*Tmprss3*	2.4536
NM_001163216	*Fgfr3*	2.4517
NM_001127382	*Rbm47*	2.449
NM_144919	*Hdac11*	2.4467
NM_024186	*Ssbp2*	2.4465
NM_030024	*Prr15*	2.4422
NM_027865	*Tmem25*	2.4421
NM_146086	*Pde6a*	2.442
NM_001302480	*Acy3*	2.4409
NM_008010	*Fgfr3*	2.4398
NM_012016	*Ern2*	2.4394
NM_138673	*Stab2*	2.4376
NM_153408	*Neurl3*	2.4323
NM_001136079	*Ptger4*	2.4195
NM_001033348	*Ralgapa2*	2.4185
NM_001159503	*Tnfsfm13*	2.4164
NM_027293	*Dopey2*	2.4121
NM_018781	*Egr3*	2.4119
NM_001162941	*Mapre2*	2.4114
NM_025912	*Fam210b*	2.4074
NM_025572	*2610528J11Rik*	2.4065
NM_001159395	*Nfkbiz*	2.403
NM_023256	*Krt20*	2.4029
NM_018746	*Itih4*	2.4029
NM_008934	*Proc*	2.4024
NM_001146690	*Chpt1*	2.399
NM_172887	*Fry*	2.3968
NM_177687	*Crebl2*	2.3932
NM_008814	*Pdx1*	2.393
NM_001290769	*Sqstm1*	2.3914
NM_001110218	*Ppm1h*	2.3865
NM_008804	*Pde9a*	2.3858
NM_001136556	*Fer1l4*	2.3858
NM_133218	*Zfp704*	2.3826
NM_177358	*Zfp945*	2.3817
NM_008131	*Glul*	2.3812
NM_010751	*Mxd1*	2.3788
NM_011303	*Dhrs3*	2.3782
NM_013454	*Abca1*	2.3756
NM_016917	*Slc40a1*	2.3746
NM_013605	*Muc1*	2.3724
NM_007819	*Cyp3a13*	2.3711

NM_019753	*Cdh17*	2.3679
NM_001276412	*Fn1*	2.3658
NM_013464	*Ahr*	2.3655
NM_144828	*Ppp1r1b*	2.3652
NM_139307	*Vasn*	2.3495
NM_001348159	*Cyb5a*	2.3481
NM_028048	*Slc25a35*	2.3408
NM_010156	*Samd9l*	2.3331
NM_011324	*Scnn1a*	2.3309
NM_001199360	*Tmem164*	2.3308
NM_173781	*Rab6b*	2.319
NM_001025313	*Tapbp*	2.3163
NM_178111	*Trp53inp2*	2.3163
NM_001252401	*Tle2*	2.3152
NM_020333	*Slc12a5*	2.3132
NM_008183	*Gstm2*	2.3112
NM_010173	*Faah*	2.3058
NM_177698	*Psd3*	2.3032
NM_027988	*Noxo1*	2.2995
NM_021273	*Ckb*	2.2955
NM_138672	*Stab1*	2.2949
NM_010137	*Epas1*	2.2928
NM_008240	*Foxj1*	2.2897
NM_010145	*Ephx1*	2.2882
NM_181318	*Rasgef1b*	2.2871
NM_001042673	*Fancc*	2.2843
NM_019427	*Epb41l4b*	2.2813
NM_010168	*F2*	2.2811
NM_133220	*Sgk3*	2.2788
NM_010871	*Naip6*	2.2786
NM_001243192	*Serpinb6a*	2.2742
NM_001083318	*Etv3*	2.2732
NM_009194	*Slc12a2*	2.2727
NM_207708	*Syngr1*	2.269
NM_001042438	*Zhx1*	2.2672
NM_133902	*Sdsl*	2.2607
NM_010237	*Frk*	2.2534
NM_133990	*Il13ra1*	2.25
NM_144807	*Chpt1*	2.2491
NM_018782	*Calcrl*	2.2489
NM_008879	*Lcp1*	2.2445
NM_175236	*Adhfe1*	2.2401
NM_026376	*Plxnd1*	2.2375
NM_001347586	*Fosb*	2.2339
NM_027287	*Dnajb4*	2.2339
NM_144847	*Nrbp2*	2.2324
NM_001284248	*Hdac5*	2.2321
NM_001310530	*Chd9*	2.2311
NM_001161770	*Lmo4*	2.2306
NM_001033220	*AU021092*	2.2299
NM_146010	*Tspan8*	2.2287
NR_033228	*E030024N20Rik*	2.2257
NM_010831	*Sik1*	2.2231
NM_022331	*Herpud1*	2.2201
NM_001331120	*Anxa4*	2.217

NM_178729	*Fbxl5*	2.2156
NM_001199115	*Slc29a1*	2.2141
NM_009202	*Slc22a1*	2.214
NM_001199283	*Slc43a2*	2.2137
NM_009676	*Aox1*	2.2105
NM_031376	*Pik3ap1*	2.2061
NM_177368	*Tmtc2*	2.2041
NM_146117	*Lrrc26*	2.2037
NM_133213	*Xpnpep2*	2.2012
NM_001195253	*Gm3336*	2.2006
NM_145530	*Rhov*	2.197
NM_008043	*Frat1*	2.1945
NM_145449	*Ifi27l2b*	2.1849
NM_010098	*Opn3*	2.1849
NM_007679	*Cebpd*	2.1729
NM_016719	*Grb14*	2.1723
NM_023738	*Uba7*	2.1688
NM_010195	*Lgr5*	2.1665
NM_011693	*Vcam1*	2.1601
NM_001346658	*Ube2q2*	2.1599
NR_040528	*Gm9926*	2.1577
NM_177383	*Gpr21*	2.1445
NM_001287166	*Nup98*	2.1443
NM_144731	*Galnt7*	2.1433
NM_029870	*Crebrf*	2.1385
NM_030728	*Cemip*	2.1384
NM_007498	*Atf3*	2.1374
NM_021877	*Hr*	2.1369
NM_001002268	*Adgrg6*	2.1349
NM_181588	*Cmbl*	2.131
NR_033206	*Tslp*	2.1298
NM_001276409	*Fn1*	2.1273
NM_011957	*Creb3l1*	2.1263
NM_011066	*Per2*	2.1188
NM_130451	*Slc2a10*	2.1177
NM_001310445	*Slmap*	2.1174
NM_025797	*Cyb5a*	2.116
NM_144882	*Spats2l*	2.1151
NM_019820	*Cbln3*	2.113
NM_010295	*Gclc*	2.1059
NM_026316	*Aldh3b1*	2.105
NM_178257	*Il22ra1*	2.105
NM_001252374	*Nt5c3*	2.0974
NM_010127	*Pou6f1*	2.0954
NM_177084	*Slc9a4*	2.0884
NM_021344	*Tesc*	2.0883
NM_010153	*Erbb3*	2.0806
NM_010398	*H2-T23*	2.0798
NM_001304831	*Maff*	2.0787
NM_010444	*Nr4a1*	2.0734
NM_010493	*Icam1*	2.0655
NM_030127	*Htra3*	2.0651
NM_172295	*Mab21l3*	2.0645
NM_008756	*Ocln*	2.0638
NM_001165254	*Ctage5*	2.063

NM_001005868	*Erbb2ip*	2.0578
NM_001285831	*Far1*	2.0565
NM_016794	*Vamp8*	2.0554
NM_025745	*Erlec1*	2.0528
NM_009518	*Wnt10a*	2.0506
NM_029091	*Klc4*	2.0493
NM_023892	*Icam4*	2.0485
NM_001252442	*Phldb2*	2.0418
NM_177231	*Arrb1*	2.0385
NM_011435	*Sod3*	2.0355
NM_007994	*Fbp2*	2.0327
NM_133351	*Prss8*	2.0291
NM_009318	*Tapbp*	2.0286
NM_178382	*Flrt3*	2.028
NM_001038619	*Dnm3*	2.027
NM_007652	*Cd59a*	2.0247
NM_001136059	*Cyp1a1*	2.0211
NM_207217	*Fam234a*	2.0138
NM_011756	*Zfp36*	2.0112
NM_001317173	*Ago1*	2.0112
NM_001017959	*Lamp2*	2.01
NM_021897	*Trp53inp1*	2.0026
NM_134134	*Hmgxb3*	1.9983
NM_001025360	*Klc1*	1.9976
NM_001033322	*Gucy1a2*	1.9938
NM_001276411	*Fn1*	1.9934
NM_009900	*Clcn2*	1.9913
NM_001081270	*Dscaml1*	1.9891
NM_029031	*Shpk*	1.9854
NM_008587	*Mertk*	1.9847
NM_025768	*Grtp1*	1.9842
NM_009551	*Zfand5*	1.9835
NR_132727	*Nfe2l2*	1.9793
NM_001005863	*Mtus1*	1.9778
NM_011700	*Vill*	1.9755
NM_175130	*Trpm4*	1.9748
NR_110470	*Lncpint*	1.9719
NM_008548	*Man1a*	1.9695
NM_001277100	*Add3*	1.9679
NM_009655	*Alcam*	1.9673
NM_144942	*Csad*	1.9597
NM_172409	*Fmnl2*	1.9583
NM_009726	*Atp7a*	1.9575
NM_001159544	*Frk*	1.9567
NM_001301402	*Pir*	1.9567
NM_001161548	*Tmem184a*	1.9557
NM_013777	*Akr1c12*	1.955
NM_008668	*Nab2*	1.9528
NM_021534	*Pxmp4*	1.9505
NM_019737	*B4galt6*	1.9482
NM_021327	*Tnip1*	1.9475
NM_001320300	*Mmp28*	1.9465
NM_001111314	*Ngef*	1.9444
NM_009289	*Slk*	1.9423
NM_201239	*Rnase4*	1.9367

NM_001310465	*Pde4c*	1.9341
NM_175475	*Cyp26b1*	1.9325
NM_145839	*Rasgef1b*	1.9311
NM_021332	*Glp1r*	1.9299
NM_001146311	*Cln3*	1.9279
NM_001102455	*Aplp2*	1.9229
NM_133957	*Nfat5*	1.9222
NM_138648	*Olr1*	1.9207
NM_011390	*Slc12a7*	1.9176
NM_019971	*Pdgfc*	1.9061
NM_172614	*Tmem44*	1.9054
NM_031176	*Tnxb*	1.9038
NM_178589	*Tnfrsf21*	1.8977
NM_001252505	*St6gal1*	1.8928
NM_172723	*Adap1*	1.8883
NM_177390	*Myo1d*	1.8829
NM_001205157	*Yipf2*	1.8798
NM_001198894	*Adgrg1*	1.8769
NR_105023	*1010001N08Rik*	1.8708
NM_152803	*Hpse*	1.87
NM_025877	*Slc25a23*	1.8691
NM_001310720	*P2rx4*	1.8658
NM_015731	*Atp9a*	1.8637
NM_053273	*Ttyh2*	1.862
NM_033314	*Slco2a1*	1.8618
NM_013613	*Nr4a2*	1.8606
NM_080558	*Ssfa2*	1.8601
NM_019580	*Gde1*	1.8579
NM_001163131	*Elf3*	1.8553
NM_001310727	*Kmt5a*	1.8495
NM_027153	*Pir*	1.846
NM_025334	*Txndc12*	1.8372
NM_001271898	*Acox1*	1.832
NM_028326	*Zfp618*	1.8312
NM_001026214	*Entpd5*	1.8263
NM_177338	*Hmbox1*	1.8263
NM_029057	*Tbc1d30*	1.8251
NM_001081177	*Kif13b*	1.8234
NM_026680	*Golt1a*	1.822
NM_175105	*Aqp11*	1.8208
NM_007620	*Cbr1*	1.8198
NM_153560	*Fam102a*	1.818
NM_008542	*Smad6*	1.8168
NM_001313914	*Thbs1*	1.8137
NM_001042615	*Htra3*	1.8114
NM_133986	*Tcta*	1.8087
NM_001331223	*Luc7l2*	1.8063
NM_177606	*Plekhh2*	1.8062
NM_144794	*Tmem63a*	1.8024
NM_001162492	*Mpst*	1.7996
NM_172442	*Dtx4*	1.7993
NM_029436	*Klhl24*	1.7983
NM_199307	*Ece1*	1.7981
NM_172760	*Elmo3*	1.7979
NM_001163749	*Camsap3*	1.7978

NM_134188	*Acot2*	1.7965
NM_138587	*Fam3c*	1.7943
NM_013495	*Cpt1a*	1.7866
NM_134042	*Aldh6a1*	1.783
NM_029332	*Akap13*	1.7797
NM_028133	*Egln3*	1.7792
NM_011580	*Thbs1*	1.7749
NM_001037745	*Zfp791*	1.7733
NM_178283	*Asb13*	1.7671
NM_008969	*Ptgs1*	1.7662
NM_001312644	*Ttc6*	1.7605
NM_009994	*Cyp1b1*	1.752
NM_001113417	*Thrb*	1.7503
NM_001123382	*Il1r1*	1.7502
NM_001113529	*Csf1*	1.7489
NM_181547	*Nostrin*	1.7467
NM_153781	*Pygb*	1.7464
NM_175528	*E330009J07Rik*	1.7455
NR_015566	*A330023F24Rik*	1.7452
NM_010637	*Klf4*	1.744
NM_177993	*Hbp1*	1.7418
NM_134012	*Mbtd1*	1.7415
NM_174851	*Ifnlr1*	1.7404
NM_001077696	*Hdac5*	1.7385
NR_037610	*Pafah1b1*	1.7363
NM_008483	*Lamb2*	1.7357
NM_009624	*Adcy9*	1.7356
NM_031185	*Akap12*	1.7339
NM_001102437	*Acbd5*	1.7303
NM_001112698	*Ngf*	1.7295
NM_001025262	*Tpd52*	1.7253
NM_001347408	*Crb3*	1.7246
NM_009468	*Dpysl3*	1.7215
NM_001291227	*Slit2*	1.7214
NM_008055	*Fzd4*	1.7203
NM_175023	*Rbbp6*	1.7184
NM_181820	*Tmc4*	1.7157
NM_019588	*Plce1*	1.7152
NM_001113545	*Lima1*	1.7147
NM_173866	*Gpt2*	1.7135
NM_001081084	*Cubn*	1.7085
NM_009152	*Sema3a*	1.7054
NM_001331112	*Aldh3a1*	1.704
NM_011595	*Timp3*	1.7035
NM_008767	*Slc22a18*	1.7011
NM_024479	*Wbscr27*	1.6963
NM_181585	*Pik3r3*	1.6952
NM_015729	*Acox1*	1.6945
NM_031251	*Ctns*	1.6904
NM_001039533	*Pdxdc1*	1.6904
NM_001347332	*Pisd*	1.6877
NM_001005426	*Zcwpw1*	1.6841
NM_027171	*Camsap3*	1.6824
NM_016772	*Ech1*	1.6772
NM_009443	*Tgoln1*	1.6758

NM_030261	*Sesn3*	1.6685
NM_030094	*Nudt17*	1.6645
NM_001271005	*H2-T-ps*	1.6635
NM_008616	*Zfp239*	1.662
NM_001165902	*Ctnnb1*	1.6618
NM_023537	*Rab3b*	1.6604
NM_007556	*Bmp6*	1.659
NM_009543	*Rnf103*	1.658
NM_023158	*Cxcl16*	1.6545
NM_181848	*Optn*	1.6541
NM_001281819	*Ace*	1.6541
NM_021504	*Ngly1*	1.653
NM_010723	*Lmo4*	1.652
NM_001001885	*Tmem151a*	1.6462
NM_001081205	*Nipal1*	1.6458
NM_001290728	*Sytl5*	1.6449
NM_001311117	*Larp4b*	1.6422
NM_001168680	*Tspan8*	1.6402
NM_130447	*Dusp16*	1.6385
NM_001114174	*Fam189a2*	1.6301
NM_011018	*Sqstm1*	1.6278
NM_009864	*Cdh1*	1.6249
NM_011436	*Sorl1*	1.6241
NM_029563	*Rap1gap*	1.6239
NM_010748	*Lyst*	1.6205
NM_001286039	*Klc3*	1.62
NM_008512	*Lrp1*	1.6165
NM_009459	*Ube2h*	1.6124
NM_183161	*Slc17a9*	1.6123
NM_013855	*Abca3*	1.6073
NM_201362	*Ccdc68*	1.6064
NM_016867	*Gipc2*	1.6008
NM_144916	*Tmem150a*	1.6
NM_145470	*Deptor*	1.599
NM_172142	*Nfkbid*	1.5935
NM_001177369	*Nfkb2*	1.5872
NM_144549	*Trib1*	1.5862
NM_001037987	*Edil3*	1.5846
NM_021610	*Gpa33*	1.5839
NM_001162503	*Sqrdl*	1.5825
NM_172694	*Megf9*	1.58
NM_007436	*Aldh3a1*	1.5738
NM_001038602	*Marveld2*	1.5676
NM_025705	*Dcbld1*	1.5664
NM_024272	*Ssbp2*	1.5645
NM_007464	*Birc3*	1.5638
NM_145973	*Ell3*	1.5628
NR_102313	*Stap2*	1.5625
NR_002847	*Malat1*	1.5602
NM_177638	*Crb3*	1.5571
NM_153392	*Ttc39a*	1.5542
NM_144812	*Tnrc6b*	1.5531
NM_016893	*Fut8*	1.5513
NM_011116	*Pld3*	1.5447
NM_008321	*Id3*	1.5432

NM_001033225	*Pnrc1*	1.5423
NM_029011	*Pyroxd2*	1.5404
NM_001002267	*Tmem158*	1.539
NM_008706	*Nqo1*	1.5364
NM_001081383	*Kmt2c*	1.5302
NM_025685	*Col27a1*	1.5238
NM_025968	*Ptgr1*	1.5189
NM_001276410	*Fn1*	1.5155
NM_029600	*Abcc3*	1.5153
NM_029007	*Fam84a*	1.5135
NM_133857	*Usp53*	1.5105
NM_001199105	*Trp53inp1*	1.5081
NM_025326	*Tmem176a*	1.5079
NM_001081009	*Parp8*	1.5068
NM_009121	*Sat1*	1.5059
NM_009061	*Rgs2*	1.5034
NM_008532	*Epcam*	1.4987
NM_001252570	*Phyhd1*	1.4941
NR_033218	*Rreb1*	1.4914
NM_027823	*Arhgap42*	1.4912
NM_023324	*Peli1*	1.4912
NM_001146010	*Fchsd2*	1.4912
NM_011361	*Sgk1*	1.4904
NM_022721	*Fzd5*	1.4891
NM_011851	*Nt5e*	1.4863
NM_207255	*Zfp532*	1.4862
NM_023056	*Tmem176b*	1.4827
NR_037569	*1810026B05Rik*	1.4823
NM_153529	*Nrn1*	1.481
NM_001347513	*Fgfr1op2*	1.4785
NM_134091	*Sgsm3*	1.4747
NM_008330	*Ifi47*	1.4714
NM_001083628	*Greb1l*	1.47
NM_008135	*Slc6a9*	1.468
NM_025835	*Pccb*	1.4661
NM_011610	*Tnfrsf1b*	1.4635
NM_145220	*Appl2*	1.4602
NM_013471	*Anxa4*	1.4557
NM_010171	*F3*	1.4529
NM_007921	*Elf3*	1.4501
NM_023245	*Palmd*	1.4476
NM_013760	*Dnajb9*	1.4471
NR_138591	*Rap1gap*	1.4467
NM_001199137	*Macf1*	1.4463
NM_016780	*Itgb3*	1.4441
NM_001324531	*Rab3d*	1.4411
NM_026346	*Fbxo32*	1.4392
NM_001114328	*Ccpg1*	1.4376
NM_178118	*Dixdc1*	1.4374
NM_029639	*Plet1*	1.436
NM_026384	*Dgat2*	1.4352
NM_001284506	*Plxnb2*	1.4348
NM_009804	*Cat*	1.4339
NM_001130450	*Nfe2l1*	1.4302
NM_019739	*Foxo1*	1.4272

NM_001001981	*Utp14b*	1.426
NM_028710	*Arsg*	1.4229
NM_139227	*Atxn7*	1.4215
NM_027379	*Far1*	1.4195
NM_153159	*Zc3h12a*	1.4166
NM_007437	*Aldh3a2*	1.4159
NM_011530	*Tap2*	1.412
NM_016985	*Mtmr1*	1.4116
NM_007570	*Btg2*	1.4113
NM_008259	*Foxa1*	1.4107
NM_001198831	*Ddr1*	1.4085
NM_176839	*Abcc5*	1.4065
NM_016974	*Dbp*	1.403
NM_138756	*Slc25a36*	1.4018
NR_001463	*Xist*	1.4013
NM_001083906	*Nr3c2*	1.401
NM_053144	*Pcdhb19*	1.3992
NM_001033167	*Slc22a23*	1.3981
NM_178920	*Mal2*	1.3937
NM_194342	*Sun2*	1.393
NM_007592	*Car8*	1.3919
NR_015459	*4933431E20Rik*	1.3914
NM_008681	*Ndrg1*	1.3874
NM_011925	*Adgre5*	1.3864
NM_144848	*Eppk1*	1.3856
NM_028810	*Rnd3*	1.3854
NM_001316737	*Fam213a*	1.3842
NM_001038695	*Kdm3a*	1.3832
NM_001303423	*Aldoc*	1.3827
NM_019417	*Pdlim4*	1.3793
NM_001291065	*Foxa2*	1.3757
NM_153577	*Syne4*	1.3726
NM_001197147	*Slc4a4*	1.3722
NM_001198826	*App*	1.3696
NM_001130165	*Oxr1*	1.3678
NM_144800	*Mtss1*	1.3633
NM_028724	*Rin2*	1.3616
NM_001037847	*Cnot2*	1.3601
NM_001109757	*Atp7a*	1.3597
NM_001113460	*Tec*	1.3591
NM_133858	*Fam63a*	1.3586
NM_001286031	*Dhx32*	1.3585
NM_001033537	*Pgap3*	1.3574
NM_001163314	*Pgap1*	1.3568
NM_013778	*Akr1c13*	1.356
NM_009178	*St3gal4*	1.3549
NM_175350	*Catsperd*	1.3538
NM_027464	*Fam213a*	1.3511
NM_021460	*Lipa*	1.3464
NM_011448	*Sox9*	1.3389
NM_146106	*Lyplal1*	1.3388
NM_008654	*Ppp1r15a*	1.3344
NM_001177851	*Asph*	1.3339
NM_001038621	*Rabgap1l*	1.3323
NM_017391	*Slc5a3*	1.3319

NM_153198	*Hbp1*	1.3248
NM_011464	*Spint2*	1.3239
NM_199475	*Fam63a*	1.3217
NM_175557	*Zfp384*	1.3199
NM_001190854	*Pdlim5*	1.3189
NM_008012	*Akr1b8*	1.316
NM_178711	*Plscr4*	1.3148
NM_029077	*Trim14*	1.3145
NM_001130166	*Oxr1*	1.3125
NM_001199247	*Kat5*	1.312
NM_001243050	*Atp6v0a1*	1.3093
NM_001286944	*Jund*	1.3028
NM_198604	*Plekhg6*	1.2992
NM_007801	*Ctsh*	1.2988
NM_146131	*Pbxip1*	1.2976
NM_001166506	*Sec14l1*	1.2948
NM_001198824	*App*	1.2946
NM_001167981	*Galnt7*	1.2932
NM_019990	*Stard10*	1.2924
NM_023322	*Zkscan14*	1.2849
NM_001171026	*Os9*	1.2846
NM_026960	*Gsdmd*	1.2821
NM_194067	*Ifi27*	1.2814
NM_011065	*Per1*	1.2814
NM_033601	*Bcl3*	1.2806
NM_001347112	*Camsap3*	1.2793
NM_030260	*Zxdc*	1.2774
NM_010592	*Jund*	1.2764
NM_177263	*Zhx3*	1.2742
NM_001330056	*Insr*	1.2736
NM_009142	*Cx3cl1*	1.272
NM_175092	*Rhof*	1.2679
NM_018826	*Irx5*	1.2659
NM_145391	*Tapbpl*	1.2659
NR_002322	*Tug1*	1.2654
NM_009988	*Cxadr*	1.2649
NM_008057	*Fzd7*	1.2628
NM_153319	*Amot*	1.2564
NM_172282	*Tmco3*	1.2561
NM_001081286	*Fat1*	1.2526
NM_053142	*Pcdhb17*	1.2503
NM_026790	*Ifi27*	1.2477
NM_011804	*Creg1*	1.2472
NM_001083927	*Tle3*	1.2459
NR_045554	*Fut8*	1.243
NM_177732	*Slc35d1*	1.2421
NM_001166402	*Tnfaip3*	1.2412
NM_029537	*Tmem98*	1.2408
NM_011048	*Pcsk6*	1.2379
NM_027808	*Alpk1*	1.2348
NM_026931	*1810011O10Rik*	1.2338
NM_001013786	*Zscan26*	1.2322
NM_198620	*Rundc3b*	1.2306
NM_026598	*Ebpl*	1.2295
NM_001017426	*Kdm6b*	1.2294

NM_013505	*Dsc2*	1.2287
NM_178692	*C130074G19Rik*	1.2262
NM_007705	*Cirbp*	1.225
NM_146085	*Apbb3*	1.2224
NM_011027	*P2rx7*	1.2198
NM_145442	*Mbip*	1.2169
NM_173442	*Gcnt1*	1.216
NM_001102438	*Acbd5*	1.2147
NM_181404	*Kank1*	1.214
NM_013598	*Kitl*	1.2123
NM_053262	*Hsd17b11*	1.2116
NM_054056	*Pawr*	1.2091
NM_001159618	*Pigp*	1.2084
NM_145494	*Me2*	1.2053
NM_001164420	*Pqlc1*	1.2026
NM_001145884	*Itgb5*	1.2014
NR_015505	*Firre*	1.2001
NM_027144	*Arhgef12*	1.1992
NM_011074	*Cdk14*	1.1986
NM_172833	*Malt1*	1.1937
NM_022417	*Itm2c*	1.1911
NM_027855	*Atraid*	1.1911
NM_053015	*Mlph*	1.1887
NM_028787	*Slc35f5*	1.1886
NM_001113416	*Epb41l5*	1.1879
NM_028705	*Herc3*	1.1845
NR_015608	*1810058I24Rik*	1.1827
NM_001035122	*Golm1*	1.1822
NM_008390	*Irf1*	1.1801
NM_172630	*Mppe1*	1.1797
NM_001170853	*Mndal*	1.1793
NM_001289473	*Erbb2ip*	1.1792
NM_001014390	*Dyrk2*	1.1774
NM_146018	*Flcn*	1.1753
NM_030018	*Tmem50b*	1.1739
NM_145070	*Hip1r*	1.1736
NM_001128094	*Atp13a3*	1.1733
NM_027570	*Ldhd*	1.1687
NM_028864	*Zc3hav1*	1.1678
NM_008434	*Kcnq1*	1.1639
NM_001313983	*Thra*	1.1623
NM_080795	*Lnx2*	1.1603
NM_008983	*Ptprk*	1.1585
NM_001291503	*Birc2*	1.157
NR_030762	*Snord17*	1.1545
NM_172161	*Irak2*	1.1541
NM_028846	*Usp20*	1.1538
NM_007569	*Btg1*	1.1529
NM_007487	*Arl4a*	1.1519
NM_010560	*Il6st*	1.149
NM_009427	*Tob1*	1.1486
NM_026013	*Dram2*	1.1467
NM_007564	*Zfp36l1*	1.1434
NM_001033606	*Acsl3*	1.1421
NM_023842	*Dsp*	1.1416

NM_007920	*Elf1*	1.1398
NM_001177871	*Filip1l*	1.1372
NM_028995	*Nipal3*	1.1369
NM_009982	*Ctsc*	1.1321
NM_001271358	*Arrb2*	1.1314
NM_001308072	*Gpr108*	1.1311
NM_030554	*Rab27b*	1.1307
NM_016762	*Matn2*	1.13
NM_001347626	*Hmbox1*	1.1295
NM_133770	*Adck4*	1.1281
NM_007471	*App*	1.1281
NM_153122	*Oplah*	1.1279
NM_026160	*Map1lc3b*	1.1268
NM_001290678	*Cpeb4*	1.1266
NM_001082548	*Spint2*	1.1238
NM_001168491	*Pdcd4*	1.1222
NM_178060	*Thra*	1.1163
NM_011663	*Zrsr1*	1.1156
NM_133648	*Slc12a6*	1.1151
NM_001077192	*Abi1*	1.1145
NM_133750	*Fam118a*	1.1138
NM_001198825	*App*	1.1131
NM_178446	*Rbm47*	1.1129
NM_178357	*Klf11*	1.1124
NM_022018	*Fam129a*	1.111
NM_001165984	*Ubap2l*	1.1106
NM_001159354	*Magi3*	1.1071
NM_144923	*Blvrb*	1.1013
NM_023635	*Rab27a*	1.0957
NM_001033156	*Fbxo33*	1.0941
NM_181073	*Plekhh1*	1.0941
NM_011656	*Tuft1*	1.0895
NM_029847	*Arsk*	1.0887
NM_178764	*Fam168a*	1.0882
NM_207681	*Bcl2l11*	1.0857
NM_001044719	*D17Wsu92e*	1.0856
NM_008048	*Igfbp7*	1.0835
NM_028343	*Tmem135*	1.0823
NM_172672	*Ganc*	1.0778
NM_001081069	*Rgs11*	1.0761
NM_029466	*Arl5b*	1.0752
NM_009124	*Atxn1*	1.0748
NR_033629	*BC021767*	1.0747
NM_199446	*Phkb*	1.0663
NM_178704	*Dpy19l3*	1.0656
NM_007520	*Bach1*	1.062
NM_029075	*Stx11*	1.0603
NM_018811	*Abhd2*	1.0594
NM_172775	*Plxnb1*	1.0567
NM_183208	*Zmiz1*	1.0542
NM_153807	*Acsf2*	1.0526
NM_019437	*Rfk*	1.0519
NM_009615	*Adam17*	1.0502
NM_001289926	*2010111I01Rik*	1.0496
NM_001081982	*Nfix*	1.0469

NM_001136067	*Ikbkg*	1.0446
NM_007509	*Atp6v1b2*	1.0426
NM_173006	*Pon3*	1.0381
NM_008287	*Rida*	1.0344
NM_178648	*Ubxn8*	1.0335
NM_011056	*Pde4d*	1.0333
NM_145359	*Ubald1*	1.0328
NM_001004176	*Maml3*	1.032
NM_001206335	*Fam234a*	1.0303
NM_001039692	*Arhgap12*	1.0294
NM_028194	*Fryl*	1.0284
NM_001198823	*App*	1.0284
NM_008872	*Plat*	1.0278
NM_001164185	*Lsr*	1.0263
NM_020295	*Lmbr1*	1.0222
NM_010511	*Ifngr1*	1.0193
NM_010515	*Igf2r*	1.0192
NM_011809	*Ets2*	1.0184
NM_026617	*Tmbim4*	1.0143
NM_021350	*Chml*	1.0137
NM_001045489	*Mfge8*	1.0132
NR_073190	*Chka*	1.0129
NM_007533	*Bckdha*	1.0118
NM_172529	*Gnptg*	1.011
NM_146168	*Vopp1*	1.0044
NM_001085450	*Ctnnd1*	1.0039
NM_146133	*Golph3l*	1.0034
NR_003513	*Neat1*	1.0032
NM_010726	*Phyh*	1.003
NM_019831	*Zmym3*	1.0014
NM_172584	*Itpk1*	-1.0005
NM_028349	*Sass6*	-1.001
NM_001081265	*Dnaaf5*	-1.0011
NM_001113564	*Serbp1*	-1.0019
NM_013531	*Gnb4*	-1.0024
NM_133757	*Pgs1*	-1.0032
NM_177620	*Rin3*	-1.0033
NM_001159482	*Rab34*	-1.0042
NM_001039556	*Rad54b*	-1.0052
NM_009951	*Igf2bp1*	-1.0065
NM_008451	*Klc2*	-1.0069
NM_019428	*Rpp30*	-1.007
NM_009931	*Col4a1*	-1.0078
NM_029655	*Snx7*	-1.0084
NM_178208	*Hist1h4c*	-1.0111
NM_026599	*Cgnl1*	-1.0112
NM_001252219	*Rpl31*	-1.0113
NM_178605	*Nop16*	-1.0118
NM_011232	*Rad1*	-1.0119
NM_001014996	*Cenpj*	-1.0134
NM_198605	*Ska3*	-1.0137
NM_198001	*1110008P14Rik*	-1.0142
NM_025642	*Mis18a*	-1.0144
NM_026742	*Ndufaf4*	-1.0155
NM_033509	*Vangl2*	-1.0156

NM_178199	*Hist1h2bl*	-1.0158
NM_017397	*Ddx20*	-1.0165
NM_175661	*Hist1h2af*	-1.0179
NM_008445	*Kif3c*	-1.0194
NM_001313880	*Hist1h2bq*	-1.0198
NM_008576	*Abcc1*	-1.02
NM_007457	*Ap1s1*	-1.0205
NM_080850	*Pask*	-1.0206
NM_001253808	*Racgap1*	-1.021
NM_152804	*Plk2*	-1.021
NM_010288	*Gja1*	-1.0217
NM_175554	*Clspn*	-1.0217
NM_011623	*Top2a*	-1.0226
NM_153556	*Pms1*	-1.0226
NM_144835	*Heatr1*	-1.0226
NM_027740	*Poc1b*	-1.0227
NM_009773	*Bub1b*	-1.0231
NM_001035123	*Setd6*	-1.0231
NM_009223	*Snn*	-1.024
NM_178591	*Nrg1*	-1.0242
NM_021385	*Rad18*	-1.0245
NM_001271397	*Nol8*	-1.0249
NM_028121	*Adpgk*	-1.0251
NM_013549_dup1	*Hist2h2aa1*	-1.0253
NM_001004144	*Git1*	-1.0255
NM_019833	*Fam69b*	-1.0256
NM_001130153	*Arhgef1*	-1.0258
NM_178212_dup1	*Hist2h2aa2*	-1.027
NM_001177658	*Mrpl15*	-1.027
NM_009103	*Rrm1*	-1.0274
NM_013733	*Chaf1a*	-1.0277
NM_080793	*Setd7*	-1.028
NM_024193	*Nop56*	-1.0282
NM_026438	*Ppa1*	-1.0289
NM_133685	*Rab31*	-1.0297
NM_177767	*Ogfod1*	-1.0302
NM_026197	*Mettl16*	-1.0309
NM_175655	*Hist1h4f*	-1.031
NM_001252200	*Map4k4*	-1.0314
NM_010119	*Ehd1*	-1.0315
NM_025447	*Dimt1*	-1.0317
NM_001042660	*Smad7*	-1.0319
NM_172746	*Hirip3*	-1.0327
NM_145414	*Nsun5*	-1.0329
NM_027999	*Haus5*	-1.0336
NM_008708	*Nmt2*	-1.0342
NM_009465	*Axl*	-1.0345
NM_001002011	*Lmna*	-1.0351
NM_178421	*Nanos1*	-1.0353
NM_001099675	*Tomm5*	-1.036
NM_133747	*Fam98a*	-1.0362
NM_026041	*Rrp15*	-1.0364
NM_033073	*Krt7*	-1.0374
NM_022890	*Cldn12*	-1.0383
NM_133786	*Smc4*	-1.0387

NM_173757	*Mrps27*	-1.0398
NM_027698	*Eri2*	-1.0399
NM_008303	*Hspe1*	-1.0401
NM_001039482	*Klhl20*	-1.041
NM_001146153	*Homer3*	-1.0422
NM_013871	*Mapk12*	-1.0428
NM_026453	*Mak16*	-1.0429
NM_009689	*Birc5*	-1.0453
NR_132443	*Hist1h2al*	-1.0465
NM_025995	*Fbxo5*	-1.0473
NR_027984	*2810025M15Rik*	-1.0475
NM_023294	*Ndc80*	-1.0481
NM_023203	*Dctpp1*	-1.0481
NM_001037762	*Zdhhc12*	-1.0487
NM_021409	*Pard6b*	-1.0491
NM_019499	*Mad2l1*	-1.0494
NM_007808	*Cycs*	-1.0502
NM_010830	*Msh6*	-1.0509
NM_144846	*Fam49b*	-1.0511
NM_172692	*Gba2*	-1.0516
NM_001177372	*Dbn1*	-1.0519
NM_016859	*Bysl*	-1.0522
NM_001033294	*Ddx31*	-1.0525
NM_026195	*Atic*	-1.0528
NM_183263	*Rnmtl1*	-1.0534
NM_177595	*Mkx*	-1.0539
NM_027193	*Dph5*	-1.0548
NM_001136082	*Timeless*	-1.0551
NM_015751	*Abce1*	-1.056
NM_178403	*Pus7*	-1.0576
NM_172784	*Lrp11*	-1.0579
NM_001199060	*Wdr12*	-1.0588
NM_144824	*Wrap53*	-1.059
NM_026403	*Nop9*	-1.0591
NM_016904	*Cks1b*	-1.06
NM_008572	*Mcpt8*	-1.06
NM_025860	*Ddx18*	-1.0602
NM_008433	*Kcnn4*	-1.0604
NM_178194	*Hist1h2be*	-1.062
NM_175234	*Faxc*	-1.0628
NM_001164655	*9530053A07Rik*	-1.0644
NM_130881	*Pabpc4*	-1.0648
NM_134149	*AI837181*	-1.0659
NM_009878	*Cdkn2d*	-1.0665
NM_029975	*Ulbp1*	-1.0666
NM_181517	*Ipo7*	-1.0667
NM_001163510	*Kcnn4*	-1.0667
NM_025599	*Cmss1*	-1.0681
NM_028315	*Dis3*	-1.0682
NM_029005	*Mlkl*	-1.0682
NM_008985	*Ptprn*	-1.0697
NM_029617	*Casc5*	-1.0701
NM_178186	*Hist1h2ag*	-1.0707
NM_001111277	*Eif2b3*	-1.0728
NM_010721	*Lmnb1*	-1.0732

NM_021330	*Acp1*	-1.0734
NM_009447	*Tuba4a*	-1.0741
NM_178215	*Hist2h3b*	-1.0742
NM_001310526	*Specc1*	-1.0749
NM_026352	*Ppid*	-1.0749
NM_001141975	*Tpx2*	-1.0752
NM_008021	*Foxm1*	-1.0753
NM_001316679	*Ptpre*	-1.0764
NM_029368	*Tex30*	-1.0767
NM_026047	*Rnf219*	-1.0775
NM_011304	*Ruvbl2*	-1.0777
NM_134090	*Kdelr3*	-1.078
NM_008233	*Hdgfrp2*	-1.0791
NM_001111311	*Lrrfip1*	-1.0792
NM_178185_dup1	*Hist1h2ap*	-1.0798
NM_001001738	*Itprip*	-1.0798
NM_178185	*Hist1h2ap*	-1.0799
NM_133962	*Arhgef18*	-1.0801
NM_029438	*Smurf1*	-1.0801
NM_009419	*Tpst2*	-1.0804
NM_053169	*Trim16*	-1.0807
NM_001290466	*Hist1h2bp*	-1.081
NM_031863	*Cenpq*	-1.0816
NM_020603	*Wdr46*	-1.0836
NM_133972	*Armc6*	-1.084
NM_026531	*Aen*	-1.0841
NM_178198	*Hist1h2bj*	-1.0844
NM_001113213	*Odf2*	-1.0849
NM_175542	*Rttn*	-1.0852
NM_029797	*Mnd1*	-1.0858
NM_021528	*Chst12*	-1.0862
NM_026913	*Mitd1*	-1.0863
NM_001033331	*Gas2l3*	-1.0864
NM_026332	*Dnajc19*	-1.0868
NM_016692	*Incenp*	-1.0871
NM_009434	*Phlda2*	-1.0878
NM_011732	*Ybx1*	-1.0882
NM_011796	*Capn10*	-1.0882
NM_029482	*4930579G24Rik*	-1.0882
NM_001048178	*Ghr*	-1.0882
NM_134074	*Dock9*	-1.0893
NM_152825	*Usp45*	-1.0896
NM_016723	*Uchl3*	-1.09
NM_028812	*Gtf2e1*	-1.0906
NM_001201413	*Apbb2*	-1.0912
NM_172824	*Ccdc14*	-1.0919
NM_010221	*Fkbp10*	-1.093
NM_001081316	*Dsel*	-1.0937
NM_007630	*Ccnb2*	-1.0939
NM_010071	*Dok2*	-1.0948
NM_001130476	*Tpst1*	-1.0955
NM_016966	*Phgdh*	-1.0955
NM_001081188	*Exosc7*	-1.0986
NM_001331154	*Hsf1*	-1.0989
NM_134092	*Mtbp*	-1.1005

NM_029999	*Lbh*	-1.1008
NM_001110209	*Lnp*	-1.1013
NM_011738	*Ywhah*	-1.1017
NM_027975	*Fam83d*	-1.1027
NM_001310708	*Sorbs2*	-1.1032
NM_001099637	*Cep170*	-1.1046
NM_175665	*Hist1h2bk*	-1.1048
NM_146083	*Srsf7*	-1.1049
NM_178184	*Hist1h2an*	-1.1057
NM_009384	*Tiam1*	-1.1066
NM_028965	*Snx11*	-1.1076
NM_001040435	*Tacc3*	-1.1077
NM_010477	*Hspd1*	-1.1082
NM_001177353	*Myc*	-1.1082
NM_145460	*Oxnad1*	-1.1097
NM_001289599	*Txndc5*	-1.1104
NM_023871	*Set*	-1.1104
NM_001042558	*Apaf1*	-1.1115
NM_026412	*Knstrn*	-1.1121
NM_023223	*Cdc20*	-1.1121
NM_027269	*Dnaaf2*	-1.1122
NM_001145924	*Msantd3*	-1.1123
NM_001042527	*Blm*	-1.1129
NM_133678	*Sac3d1*	-1.1129
NM_017407	*Spag5*	-1.114
NM_013565	*Itga3*	-1.1149
NM_181569	*Nprl3*	-1.115
NM_011638	*Tfrc*	-1.1156
NM_001081419	*Dip2a*	-1.116
NM_175137	*Vars2*	-1.1165
NM_027148	*Exosc8*	-1.1176
NM_178213	*Hist2h2ab*	-1.1189
NM_023136	*Dtymk*	-1.1214
NM_145706	*Nup43*	-1.1215
NM_018750	*Rassf5*	-1.1225
NM_001042593	*Hbs1l*	-1.1227
NM_173762	*Cenpe*	-1.1249
NM_194344	*Sh3tc1*	-1.1266
NM_001166667	*Rtel1*	-1.1278
NM_007900	*Ect2*	-1.1301
NM_001127349	*Snx10*	-1.1309
NM_008894	*Pold2*	-1.1315
NM_001162418	*Myef2*	-1.1318
NM_025377	*Ska2*	-1.1321
NM_011239	*Ranbp1*	-1.1336
NM_001114347	*Clasp2*	-1.1339
NM_176833	*Ppm1f*	-1.1349
NM_028404	*Top1mt*	-1.1352
NM_134133	*Smim3*	-1.1359
NM_026572	*Gcsh*	-1.1363
NM_001290530	*Hist1h2be*	-1.137
NM_009786	*Cacybp*	-1.1372
NM_009214	*Sms*	-1.1372
NM_133900	*Psph*	-1.1373
NM_177157	*Gchfr*	-1.1374

NM_001025365	*Miip*	-1.1374
NM_027931	*Tars2*	-1.1379
NM_177367	*Gemin4*	-1.1383
NM_172507	*Sh3bgrl2*	-1.139
NM_027354	*Poc1a*	-1.1404
NM_028984	*Mgme1*	-1.1411
NM_001283048	*Tmpo*	-1.1424
NM_153098	*Cd109*	-1.1427
NM_026028	*Ccdc77*	-1.1428
NM_019501	*Pdss1*	-1.1439
NM_001013026	*Ttf2*	-1.144
NM_016903	*Esd*	-1.1444
NM_213614	*43348*	-1.1444
NM_001252089	*Rbmxl1*	-1.1445
NM_019761	*Nxt1*	-1.1447
NM_028028	*Zswim1*	-1.1448
NM_001081130	*Ogdhl*	-1.1454
NM_001195298	*Kifc1*	-1.1456
NM_026873	*Ptcd2*	-1.1467
NM_007945	*Eps8*	-1.148
NR_024078	*Btbd19*	-1.1483
NM_024183	*Fip1l1*	-1.1485
NM_001146084	*Fastkd5*	-1.1485
NM_010790	*Melk*	-1.1496
NM_028599	*Wdr75*	-1.1511
NM_013508	*Elk3*	-1.1517
NM_010590	*Ajuba*	-1.1519
NM_001111102	*Lmna*	-1.1526
NM_016925	*Fanca*	-1.1527
NM_028072	*Sulf2*	-1.153
NM_130450	*Elovl6*	-1.1536
NM_010852	*Myef2*	-1.1539
NM_025508	*Gmpr*	-1.1551
NM_011602	*Tln1*	-1.1555
NM_009278	*Ssb*	-1.1561
NM_001033256	*Spata5l1*	-1.1565
NM_031999	*Gpr137b*	-1.1566
NM_001177354	*Myc*	-1.1566
NM_028228	*Pinx1*	-1.1568
NM_146235	*Ercc6l*	-1.1572
NM_001289898	*Csnk1e*	-1.1587
NM_177342	*Taf5*	-1.1588
NM_001012517	*Fut10*	-1.1588
NM_007659	*Cdk1*	-1.1606
NM_008697	*Nin*	-1.1618
NM_026446	*Rgs19*	-1.1622
NM_178182	*Hist1h2ai*	-1.1634
NM_133835	*Ubac1*	-1.1645
NM_199447	*Rrp12*	-1.1647
NM_013552	*Hmmr*	-1.1648
NM_019939	*Mpp6*	-1.1648
NM_001164248	*Tpm1*	-1.1656
NM_021891	*Fignl1*	-1.1663
NM_178204	*Hist1h3d*	-1.169
NM_001080775	*Myo1c*	-1.1691

NM_001287179	*Kif14*	-1.1708
NM_026421	*Enoph1*	-1.1717
NM_011175	*Lgmn*	-1.1731
NM_012027	*Mprip*	-1.1743
NM_175662	*Hist2h2ac*	-1.1746
NM_175653	*Hist1h3c*	-1.1757
NM_001146325	*Tigit*	-1.1766
NM_139117	*Ybx3*	-1.1767
NM_008705	*Nme2*	-1.1775
NM_009601	*Chrnb1*	-1.1783
NM_001044384	*Timp1*	-1.18
NM_001331043	*Abi1*	-1.1806
NM_028546	*1700066M21Rik*	-1.1838
NM_174848	*Crybg3*	-1.1839
NM_001167883	*Ankrd50*	-1.1848
NM_001081091	*Cep152*	-1.185
NM_173400	*Haus6*	-1.1866
NM_009932	*Col4a2*	-1.1867
NM_153572	*Katnal1*	-1.1876
NM_178892	*Tiparp*	-1.1877
NM_011237	*Rad9a*	-1.1887
NM_027014	*Gins1*	-1.1892
NM_001305437	*Pter*	-1.1905
NM_025689	*Ccdc51*	-1.1908
NM_172578	*Mis18bp1*	-1.1922
NM_178188	*Hist1h2ad*	-1.1934
NM_145931	*Zc3h7a*	-1.1945
NM_008122	*Gjc1*	-1.1952
NM_001081164	*Otud4*	-1.1958
NM_148952	*E2f4*	-1.1963
NM_198861	*Lrrc75a*	-1.1975
NM_029128	*Qtrtd1*	-1.1984
NM_008926	*Prkg2*	-1.1988
NM_025380	*Eef1e1*	-1.1992
NM_172770	*Ttc12*	-1.1998
NM_007404	*Adam9*	-1.2007
NM_178203	*Hist1h3b*	-1.2013
NM_001164355	*Ska1*	-1.2015
NM_153416	*Aaas*	-1.2021
NM_001142916	*Plod2*	-1.2031
NM_010139	*Epha2*	-1.204
NM_001166665	*Rtel1*	-1.2041
NM_001113179	*Bub1*	-1.2061
NM_028493	*Rhobtb3*	-1.2063
NM_134471	*Kif2c*	-1.2065
NM_178187	*Hist1h2ae*	-1.2066
NM_001025779	*Cdc6*	-1.2066
NM_178206	*Hist1h3h*	-1.2078
NM_001001882	*Rtel1*	-1.2085
NM_001111080	*Uhrf1*	-1.2095
NM_197982	*Ddx39*	-1.2112
NM_020570	*Xrcc2*	-1.2113
NM_001286203	*Timm9*	-1.212
NM_001163645	*Osbpl3*	-1.2129
NM_001146174	*Rangap1*	-1.2146

NM_145150	*Prc1*	-1.2149
NM_176987	*Simc1*	-1.2155
NM_010786	*Mdm2*	-1.216
NM_028716	*Phf19*	-1.2168
NM_025903	*Ifrd2*	-1.2169
NM_198019	*Cep78*	-1.2172
NM_172301	*Ccnb1*	-1.2196
NM_030174	*Mctp1*	-1.2206
NM_153776	*Tmem121*	-1.222
NM_146061	*Prr5*	-1.2238
NM_013614	*Odc1*	-1.2244
NM_026640	*Fam111a*	-1.2248
NM_001013577	*Inip*	-1.2249
NM_019698	*Aldh18a1*	-1.2253
NM_001177810	*Rapgef3*	-1.2255
NM_053173	*Kifc5b*	-1.227
NM_172662	*Gtdc1*	-1.2277
NM_001162932	*Rmi2*	-1.2284
NM_029573	*Idh3a*	-1.2287
NM_022811	*Polr1e*	-1.2299
NM_177672	*Tmem201*	-1.2314
NM_011369	*Shcbp1*	-1.2317
NM_026887	*Ap1s2*	-1.2324
NM_001163764	*Tcf19*	-1.2324
NM_001014976	*Espl1*	-1.2325
NM_172616	*C330027C09Rik*	-1.2332
NM_026178	*Mmd*	-1.2335
NM_138747	*Nop2*	-1.2336
NM_009828	*Ccna2*	-1.234
NM_001144988	*Spg20*	-1.2349
NM_201389	*Plec*	-1.2351
NM_015771	*Lats2*	-1.2369
NM_001166669	*Gemin5*	-1.237
NM_001130868	*Kars*	-1.2381
NM_010236	*Fpgs*	-1.24
NM_027881	*Osbpl3*	-1.2402
NM_001033484	*Iqgap3*	-1.2403
NM_001301155	*Cast*	-1.2406
NM_027127	*Gpx8*	-1.2412
NM_181589	*Ckap2l*	-1.2417
NR_028590	*Gm14005*	-1.242
NM_133664	*Lad1*	-1.2437
NM_001166408	*Rai14*	-1.2444
NM_028002	*Dus4l*	-1.2478
NM_019796	*Syncrip*	-1.2484
NM_028031	*Zdhhc13*	-1.2489
NM_011297	*Rps24*	-1.2491
NM_001081237	*Klhl42*	-1.25
NM_023525	*Cad*	-1.2502
NM_001206367	*Gsn*	-1.2506
NM_027448	*Lca5*	-1.2506
NM_009104	*Rrm2*	-1.2515
NM_028469	*3110082I17Rik*	-1.253
NM_134010	*Nup107*	-1.2531
NM_018776	*Crlf3*	-1.254

NR_027870	*Dtnb*	-1.2554
NM_001029868	*Pdzd4*	-1.2559
NM_013787	*Skp2*	-1.2561
NM_028274	*Exosc6*	-1.2562
NM_001113383	*Gls*	-1.2576
NM_001306127	*Sh2b3*	-1.258
NM_172266	*Lpgat1*	-1.2583
NM_001193309	*Morc4*	-1.2613
NM_026483	*Mphosph10*	-1.2616
NM_009772	*Bub1*	-1.2617
NM_009195	*Slc12a4*	-1.2619
NM_001290380	*Hist1h2bc*	-1.2621
NM_001081112	*Ankrd26*	-1.2629
NM_025846	*Rras2*	-1.263
NM_028230	*Shmt2*	-1.2636
NM_012012	*Exo1*	-1.2643
NM_133731	*Prss22*	-1.2651
NM_153514	*Rhobtb2*	-1.2654
NM_025564	*Magohb*	-1.2655
NM_178193	*Hist1h4b*	-1.2663
NM_023057	*Zak*	-1.2665
NM_028013	*Endod1*	-1.2668
NM_007722	*Ackr3*	-1.2673
NM_001166544	*Hmga1*	-1.2673
NM_172967	*4930503L19Rik*	-1.2686
NM_009701	*Aqp5*	-1.2702
NM_009004	*Kif20a*	-1.2728
NM_008651	*Mybl1*	-1.2733
NM_177003	*Tigar*	-1.2742
NM_001276285	*Eno3*	-1.2746
NM_001252438	*D16Ertd472e*	-1.2748
NM_009116	*Prrx2*	-1.2757
NM_172990	*Pank4*	-1.2757
NM_001348222	*Pycr1*	-1.276
NM_145409	*Chtf18*	-1.2766
NM_008127	*Gjb4*	-1.2777
NM_010655	*Kpna2*	-1.2779
NM_001253688	*Immt*	-1.2799
NM_013872	*Pmm1*	-1.2815
NM_026560	*Cdca8*	-1.2824
NM_013559	*Hsph1*	-1.2828
NM_133897	*Lrrc8c*	-1.2833
NM_001271729	*Tk1*	-1.2834
NM_145620	*Rrp9*	-1.2862
NM_029840	*Tstd3*	-1.2866
NM_026646	*Slc25a22*	-1.2867
NM_025866	*Cdca7*	-1.288
NM_023209	*Pbk*	-1.2881
NM_172308	*Mthfd1l*	-1.2882
NM_001291069	*Golim4*	-1.2884
NM_010256	*Gart*	-1.292
NM_001030307	*Dkc1*	-1.2921
NM_001043335	*Eml1*	-1.2926
NM_001039392	*Tmsb10*	-1.2928
NM_010864	*Myo5a*	-1.2949

NM_001145821	*Ggta1*	-1.2957
NM_009272	*Srm*	-1.2957
NM_153792	*Traf7*	-1.2965
NM_175265	*Bora*	-1.2971
NM_021515	*Ak1*	-1.2971
NM_026410	*Cdca5*	-1.2972
NM_011567	*Tead4*	-1.2976
NM_145758	*0610010K14Rik*	-1.2989
NM_145964	*Ap1ar*	-1.3008
NM_001252439	*D16Ertd472e*	-1.3013
NM_016759	*Rundc3a*	-1.3018
NM_011919	*Ing1*	-1.3023
NM_011075	*Abcb1b*	-1.3026
NM_026039	*Med18*	-1.3029
NM_011496	*Aurkb*	-1.3032
NR_027509	*Suv39h2*	-1.3034
NM_031182	*Tfap4*	-1.3035
NM_133914	*Rasa4*	-1.3042
NM_026785	*Ube2c*	-1.3056
NM_145489	*AI661453*	-1.3063
NM_001081333	*Plekhg4*	-1.3068
NM_027667	*Arhgap19*	-1.3072
NM_177372	*Dna2*	-1.3095
NM_177781	*Trpa1*	-1.3098
NM_023120	*Gnb1l*	-1.3103
NM_145950	*Osgin2*	-1.3116
NM_027411	*Spdl1*	-1.3119
NM_001004140	*Ckap2*	-1.3124
NM_173376	*Rbmx2*	-1.3124
NM_025581	*Ska1*	-1.3124
NM_023240	*Eef1d*	-1.3146
NM_145495	*Rin1*	-1.3147
NM_029762	*Hyls1*	-1.3154
NM_008485	*Lamc2*	-1.3155
NM_175659	*Hist1h2ah*	-1.3156
NM_001042699	*Syne3*	-1.3179
NM_028185	*Lsm11*	-1.3185
NM_182927	*Spred3*	-1.3209
NM_001141977	*Tpx2*	-1.3225
NM_024241	*Kif24*	-1.3228
NM_008652	*Mybl2*	-1.3236
NM_011763	*Zfp9*	-1.3243
NM_145073	*Hist1h3g*	-1.3289
NM_020594	*Zc3h8*	-1.329
NM_021511	*Rrs1*	-1.3291
NM_001302803	*Mdm4*	-1.3291
NM_007525	*Bard1*	-1.3296
NM_172562	*Tada2a*	-1.3298
NM_001099631	*Sh2d5*	-1.3326
NM_001316365	*Etv4*	-1.333
NM_001167730	*Rad18*	-1.3337
NM_023173	*Dusp12*	-1.334
NM_029249	*Parpbp*	-1.3342
NM_001003898	*Tardbp*	-1.3352
NM_001284328	*Syncrip*	-1.3377

NM_001164528	*Ildr2*	-1.3385
NM_013925	*Adat1*	-1.3397
NM_178643	*Faap24*	-1.3408
NR_130895	*Clca3a1*	-1.341
NM_175656	*Hist1h4i*	-1.3447
NM_001190717	*Dbf4*	-1.3459
NM_019752	*Htra2*	-1.3464
NM_025980	*Nrarp*	-1.3466
NM_001253736	*Pdlim2*	-1.3476
NM_197990	*1700025G04Rik*	-1.3489
NM_001013377	*Arhgef39*	-1.349
NM_172564	*Tns4*	-1.3496
NM_001160292	*Celf2*	-1.3505
NM_001111312	*Lrrfip1*	-1.353
NM_019670	*Diaph3*	-1.3533
NM_019813	*Dbn1*	-1.3537
NM_023317	*Nde1*	-1.3541
NM_029858	*Ston1*	-1.3546
NM_026331	*Slc25a37*	-1.3557
NM_007725	*Cnn2*	-1.356
NM_001320554	*Rcor2*	-1.3598
NM_001159866	*Uchl5*	-1.3601
NM_010892	*Nek2*	-1.3605
NM_153060	*Spns2*	-1.3626
NM_178743	*Slc26a11*	-1.3631
NM_009805	*Cflar*	-1.3641
NM_008808	*Pdgfa*	-1.3646
NM_013750	*Phlda3*	-1.3656
NM_009929	*Col18a1*	-1.3657
NM_013742	*Cars*	-1.3669
NM_001198984	*Tcof1*	-1.3679
NM_001013405	*Tcaim*	-1.368
NM_001290378	*Ap1s2*	-1.3682
NM_001308300	*Ggta1*	-1.3683
NM_027091	*Nup35*	-1.3694
NM_023284	*Nuf2*	-1.3698
NM_008495	*Lgals1*	-1.3728
NM_153554	*Aldh18a1*	-1.3729
NM_001172093	*Depdc1a*	-1.3736
NM_009445	*Ttk*	-1.3738
NM_008880	*Plscr2*	-1.3767
NR_028591	*Gm14005*	-1.3772
NM_011653	*Tuba1a*	-1.3774
NM_027172	*Slc52a3*	-1.3778
NM_207161	*Dnph1*	-1.3799
NM_001164118	*Serpinb6a*	-1.3821
NM_027191	*Nup37*	-1.3845
NM_001004361	*Tpgs2*	-1.3854
NM_011121	*Plk1*	-1.3862
NM_001144987	*Spg20*	-1.3863
NM_013825	*Ly75*	-1.3883
NR_015555	*4933404O12Rik*	-1.3894
NM_001110195	*Echdc1*	-1.3897
NM_199240	*Sema6d*	-1.39
NM_053083	*Loxl4*	-1.3905

NM_026619	*Gsto2*	-1.3906
NM_001300827	*Poglut1*	-1.3913
NM_001282943	*Ccne2*	-1.3921
NM_019681	*Ncs1*	-1.3923
NM_007918	*Eif4ebp1*	-1.3935
NM_001289716	*Bcl2l1*	-1.3949
NM_011634	*Traip*	-1.3964
NM_001122683	*Bdh1*	-1.3979
NM_001114597	*Ehbp1l1*	-1.3989
NM_027875	*Syde1*	-1.3995
NM_201394	*Plec*	-1.3997
NM_172285	*Plcg2*	-1.401
NM_001081079	*Ogfrl1*	-1.402
NM_001085407	*Sdccag3*	-1.4043
NM_007857	*Dhh*	-1.4046
NM_001301412	*Smc2*	-1.4047
NM_177374	*Trmt61a*	-1.4068
NM_172437	*Pus7l*	-1.4075
NM_198299	*Enkd1*	-1.4076
NM_080448	*Srgap3*	-1.4079
NM_029413	*Morc4*	-1.4086
NM_001168333	*Tinagl1*	-1.4104
NM_026507	*Zwilch*	-1.4143
NM_001285981	*Banp*	-1.4152
NM_001134646	*Tomm5*	-1.4177
NR_136938	*Adamts6*	-1.4185
NM_010070	*Dok1*	-1.4193
NM_010833	*Msn*	-1.4217
NM_001141978	*Tpx2*	-1.4229
NM_053180	*Cdk20*	-1.4255
NM_009728	*Atp10a*	-1.4265
NM_028232	*Sgol1*	-1.4275
NM_021881	*Qk*	-1.4288
NM_026454	*Ube2f*	-1.43
NM_153504	*Rnf183*	-1.4323
NM_153142	*Slc35e4*	-1.4335
NM_028022	*Gatsl3*	-1.4337
NM_175660	*Hist1h2ab*	-1.4343
NM_027182	*Trip13*	-1.4346
NM_175664	*Hist1h2bb*	-1.4353
NM_201531	*Kcnf1*	-1.4353
NM_134079	*Adk*	-1.4356
NM_024184	*Asf1b*	-1.4365
NM_016696	*Gpc1*	-1.4384
NM_009387	*Tk1*	-1.4394
NM_133878	*Rcc1*	-1.4409
NM_010929	*Notch4*	-1.441
NM_153459	*Dusp7*	-1.4457
NM_177466	*Rab11fip5*	-1.4495
NM_001285503	*Nde1*	-1.4517
NM_053178	*Acsbg1*	-1.4537
NM_144553	*Dlgap5*	-1.4542
NM_026340	*Pop1*	-1.457
NM_009794	*Capn2*	-1.4577
NM_010634	*Fabp5*	-1.4581

NM_001079876	*Gas2l3*	-1.4581
NM_001122982	*Ccnc*	-1.4581
NM_001305631	*Cenpi*	-1.4583
NM_177331	*Gen1*	-1.461
NM_007634	*Ccnf*	-1.4632
NM_001206368	*Gsn*	-1.4648
NM_177420	*Psat1*	-1.4672
NM_001110147	*Tnk2*	-1.4695
NM_008404	*Itgb2*	-1.4707
NM_175382	*Fam72a*	-1.4708
NM_199007	*Sgol2a*	-1.4715
NM_011893	*Sh3bp2*	-1.4719
NM_001166064	*Syde2*	-1.4751
NM_146260	*Tmie*	-1.4776
NM_198654	*Nsl1*	-1.4787
NM_001164362	*Cep55*	-1.4813
NM_001290830	*Trp53bp1*	-1.4818
NM_001103156	*Steap2*	-1.4822
NM_001103162	*Scap*	-1.4829
NM_146127	*Fam110a*	-1.4831
NM_139303	*Kif18a*	-1.4834
NM_134161	*Fut10*	-1.4846
NM_133167	*Parvb*	-1.4847
NR_027869	*Dtnb*	-1.4849
NM_016748	*Ctps*	-1.4858
NM_177752	*Eme1*	-1.487
NM_175170	*Pogk*	-1.4879
NM_001012273	*Birc5*	-1.4904
NM_173788	*Npr2*	-1.4907
NM_153143	*Kctd11*	-1.4951
NM_144797	*Metrnl*	-1.4951
NM_144526	*Fam64a*	-1.4967
NM_133859	*Olfml3*	-1.4983
NM_007739	*Col8a1*	-1.5013
NM_144515	*Zfp52*	-1.5021
NM_001164791	*Zak*	-1.5023
NM_012055	*Asns*	-1.5033
NM_001146081	*Fancb*	-1.504
NM_178609	*E2f7*	-1.5045
NR_003568	*Gpr137b-ps*	-1.5054
NM_183046	*Kif20b*	-1.5067
NM_010299	*Gm2a*	-1.5073
NM_022724	*Suv39h2*	-1.5075
NM_028523	*Dcbld2*	-1.5076
NM_021381	*Prokr1*	-1.5078
NM_001164568	*Sipa1*	-1.509
NM_001037905	*Dab2*	-1.5094
NM_023061	*Mcam*	-1.5136
NM_001243064	*Cav1*	-1.5142
NM_207653	*Cflar*	-1.5159
NM_001166477	*Hmga1-rs1*	-1.5162
NM_016787	*Bnip2*	-1.5163
NM_001081153	*Unc13c*	-1.519
NM_170689	*Ank3*	-1.5233
NM_001081176	*Polr3g*	-1.5252

NM_019438	*Ncapg*	-1.5264
NR_040721	*Snhg5*	-1.5272
NM_001163456	*Cox18*	-1.528
NM_022995	*Pmepa1*	-1.5281
NM_026721	*Slc39a13*	-1.5325
NM_178411	*Zfp334*	-1.5338
NM_001006664	*Epb41l1*	-1.536
NR_033533	*Gm12603*	-1.537
NM_001252578	*Sulf2*	-1.5373
NM_026053	*Gemin6*	-1.541
NM_025281	*Lyar*	-1.5417
NM_027982	*Ppm1j*	-1.5433
NM_026955	*Vstm5*	-1.5453
NM_025730	*Lrrk2*	-1.5454
NM_001114679	*9930111J21Rik1*	-1.5458
NM_015734	*Col5a1*	-1.551
NM_001145799	*Ctla2a*	-1.5532
NM_027134	*Mtfmt*	-1.5534
NM_008884	*Pml*	-1.5553
NM_001080995	*Ddias*	-1.5562
NM_001199124	*Spc25*	-1.5566
NM_011566	*Tead3*	-1.56
NM_001281472	*Psme2b*	-1.5606
NM_001159516	*Qk*	-1.562
NM_028390	*Anln*	-1.5622
NM_033570	*Cnnm4*	-1.5623
NM_023850	*Chst1*	-1.5663
NM_019924	*Rps6ka4*	-1.57
NM_001278075	*Farsb*	-1.5716
NM_010741	*Ly6c1*	-1.5716
NM_001146199	*Ptpn21*	-1.5755
NM_080466	*Kcnn3*	-1.5758
NM_001003955	*Rab11fip5*	-1.5774
NM_007616	*Cav1*	-1.5801
NM_001271567	*Cdc7*	-1.5822
NM_001289813	*Dgcr6*	-1.5838
NM_007796	*Ctla2a*	-1.5905
NM_001310723	*Kmt5a*	-1.5912
NM_001206369	*Gsn*	-1.5919
NM_001290369	*Nmt2*	-1.593
NM_007992	*Fbln2*	-1.5946
NM_009964	*Cryab*	-1.5954
NM_009075	*Rpia*	-1.5993
NM_001033534	*Layn*	-1.6001
NM_001290397	*Mybl1*	-1.6012
NM_011733	*Ybx3*	-1.6015
NM_001166540	*Hmga1*	-1.6052
NM_145938	*Rpp40*	-1.6072
NM_197959	*Kif18b*	-1.6074
NM_010353	*Gsg2*	-1.6082
NM_001146059	*Als2cl*	-1.6083
NM_027834	*9130008F23Rik*	-1.61
NM_010470	*Hp1bp3*	-1.6109
NM_023844	*Jam2*	-1.6112
NM_172463	*Sned1*	-1.6148

NM_009862	*Cdc45*	-1.6179
NM_001291185	*Aurka*	-1.6223
NM_018754	*Sfn*	-1.6248
NM_010903	*Nfe2l3*	-1.6248
NM_175563	*Prr11*	-1.6284
NM_212444	*Gk*	-1.6345
NM_001252055	*Ly6c1*	-1.6348
NM_009984	*Ctsl*	-1.6349
NM_001098636	*Pwwp2b*	-1.6357
NM_023476	*Tinagl1*	-1.6379
NM_028734	*Steap2*	-1.638
NM_008750	*Nxn*	-1.6398
NM_028109	*Tpx2*	-1.6453
NM_001172205	*Arid5a*	-1.6453
NM_025998	*Nkain1*	-1.6458
NM_001271526	*Orc2*	-1.6458
NM_001122830	*Klhl26*	-1.6484
NM_173733	*Suox*	-1.6485
NM_027260	*Vrk2*	-1.6509
NM_001146710	*Ppp1r18*	-1.6529
NM_177809	*Slc25a48*	-1.6529
NM_001010836	*Ppp1r13l*	-1.653
NM_001285983	*Banp*	-1.6555
NM_178674	*Fbxl21*	-1.6592
NM_024427	*Tpm1*	-1.6612
NM_001122893	*Fyn*	-1.665
NM_010441	*Hmga2*	-1.6656
NM_175439	*Mars2*	-1.6726
NM_183424	*Qrfp*	-1.6729
NM_029519	*Rap2a*	-1.6731
NM_207635	*Rps24*	-1.6763
NM_011172	*Prodh*	-1.6768
NM_172508	*Dse*	-1.6798
NM_170688	*Ank3*	-1.683
NM_007631	*Ccnd1*	-1.6879
NM_172589	*Lhfpl2*	-1.6913
NM_001038231	*Banf1*	-1.6916
NM_028176	*Cda*	-1.6956
NM_028203	*Wdr89*	-1.6971
NM_008625	*Mrc1*	-1.6981
NR_040707	*1110046J04Rik*	-1.7004
NM_021530	*Slc4a8*	-1.702
NM_146005	*Ank3*	-1.7078
NM_144895	*Spg20*	-1.7094
NM_025829	*Eif4e3*	-1.7115
NM_010248	*Gab2*	-1.7132
NM_030715	*Polh*	-1.7193
NM_001166476	*Hmga1-rs1*	-1.7201
NM_018857	*Msln*	-1.7243
NM_001033208	*Myzap*	-1.7298
NM_010553	*Il18rap*	-1.7305
NM_145376	*Lpcat1*	-1.7338
NM_008638	*Mthfd2*	-1.7413
NM_001310595	*Psma3*	-1.7436
NM_001004153	*AU018091*	-1.7461

NM_001287217	*Fxyd5*	-1.7471
NM_173429	*Zfp775*	-1.7473
NM_001081085	*Sapcd2*	-1.7483
NM_001159401	*Upp1*	-1.7487
NM_001278115	*Ube2t*	-1.7494
NM_175074	*Hmgn3*	-1.7495
NM_001168505	*Shoc2*	-1.7497
NM_001033289	*Slc9a2*	-1.7546
NM_001276489	*Ism1*	-1.755
NM_015806	*Mapk6*	-1.7553
NM_019764	*Amotl2*	-1.7572
NM_030689	*Nptxr*	-1.7576
NM_001177902	*Zfp518b*	-1.7652
NM_001110162	*Cdca2*	-1.7681
NM_001291871	*Adam17*	-1.7681
NM_021436	*Tmeff1*	-1.7703
NM_001324558	*Tipin*	-1.7719
NM_001285470	*Steap2*	-1.7752
NM_009899	*Clca3a1*	-1.7799
NM_153544	*BC030867*	-1.783
NM_172880	*Tmprss11e*	-1.7932
NM_198114	*Dagla*	-1.7938
NM_001290475	*Tdrd7*	-1.7958
NR_015483	*3110039I08Rik*	-1.7959
NM_001081249	*Vcan*	-1.7965
NM_172411	*2310007B03Rik*	-1.7993
NM_001080926	*Lrp8*	-1.8007
NM_133888	*Smpdl3b*	-1.8013
NM_001297607	*Rad23a*	-1.8043
NM_001110159	*Nxt1*	-1.8091
NM_177586	*Eif5a2*	-1.8105
NM_011097	*Pitx1*	-1.8131
NM_001029988	*Fat2*	-1.8159
NM_027158	*Upk3bl*	-1.8167
NM_026473	*Tubb6*	-1.818
NM_011520	*Sdc3*	-1.8191
NM_001081395	*Amotl1*	-1.8209
NM_021398	*Slc43a3*	-1.8219
NM_001163288	*Susd1*	-1.827
NM_010180	*Fbln1*	-1.8278
NM_001285892	*Sae1*	-1.8289
NM_007797	*Ctla2b*	-1.8349
NM_016788	*Tnk2*	-1.8374
NR_045079	*3300005D01Rik*	-1.8399
NM_178676	*Entpd3*	-1.8424
NM_001291292	*2210011C24Rik*	-1.8432
NM_181348	*Prune2*	-1.8475
NR_037996	*Hmga2-ps1*	-1.8601
NM_019819	*Dusp14*	-1.8727
NM_001039934	*Map2*	-1.873
NM_001081437	*Fbln2*	-1.874
NM_173036	*Adgrg3*	-1.8781
NM_177225	*Samd12*	-1.8785
NM_001081185	*Flnc*	-1.8805
NM_198664	*Tbc1d2*	-1.8894

NM_011950	*Mapk13*	-1.8896
NM_001001982	*A430105I19Rik*	-1.8965
NM_001290762	*Nmral1*	-1.8982
NM_001168672	*Gtse1*	-1.9011
NM_010702	*Lect2*	-1.9015
NM_009871	*Cdk5r1*	-1.902
NM_011173	*Pros1*	-1.9042
NM_001271417	*Ly6a*	-1.9055
NM_022424	*Fndc4*	-1.907
NR_015602	*F730043M19Rik*	-1.9104
NM_001302203	*Gpr87*	-1.9114
NM_001205173	*Iffo2*	-1.9143
NM_019390	*Lmna*	-1.917
NM_001077698	*Fmnl1*	-1.9192
NM_011427	*Snai1*	-1.9232
NM_007672	*Cdr2*	-1.9235
NM_001083331	*Nup88*	-1.9241
NR_028420	*Zfp809*	-1.9275
NM_011439	*Sox13*	-1.9385
NM_029094	*Pik3cb*	-1.9397
NM_010198	*Fgf11*	-1.9405
NM_194055	*Esrp1*	-1.9506
NM_001081162	*Slc4a11*	-1.9535
NM_029658	*Fam101b*	-1.9629
NM_146228	*Als2cl*	-1.9632
NM_024169	*Fkbp11*	-1.9639
NM_021424	*Nectin1*	-1.97
NM_001111016	*Nav2*	-1.9767
NM_023655	*Trim29*	-1.9793
NM_027032	*Pacrg*	-1.9833
NM_001111140	*Lrrc10b*	-1.9834
NM_001025606	*Tmem171*	-1.9839
NM_030159	*Troap*	-1.9923
NM_053086	*Nolc1*	-1.9947
NM_007532	*Bcat1*	-2.0072
NM_175386	*Lhfp*	-2.0098
NM_001290814	*Aff3*	-2.011
NM_007948	*Ercc1*	-2.0119
NM_011063	*Pea15a*	-2.0132
NM_133719	*Metrn*	-2.0153
NM_145144	*Aif1l*	-2.0171
NM_146188	*Kctd15*	-2.0185
NM_028785	*Dock8*	-2.0236
NM_001039352	*Nolc1*	-2.0275
NM_177784	*Klhl23*	-2.0281
NM_144796	*Susd4*	-2.0349
NM_001243074	*Cep57l1*	-2.0396
NM_001290765	*Slc39a13*	-2.0404
NM_176921	*6030419C18Rik*	-2.0416
NM_016693	*Map3k6*	-2.042
NM_033652	*Lmx1a*	-2.0432
NM_001025426	*Depdc5*	-2.046
NM_029612	*Slamf9*	-2.047
NM_018777	*Cldn6*	-2.0525
NM_013460	*Adra1d*	-2.0532

NR_028589	*Gm14005*	-2.0559
NM_025779	*Ccdc109b*	-2.0564
NM_018865	*Wisp1*	-2.0589
NM_011593	*Timp1*	-2.0593
NM_177600	*Ccdc73*	-2.068
NM_001145970	*Map7d1*	-2.0689
NM_020259	*Hhip*	-2.0751
NM_133237	*Apcdd1*	-2.078
NM_016894	*Ramp1*	-2.0789
NM_001081376	*Chd5*	-2.0824
NM_001271418	*Ly6a*	-2.0841
NM_008885	*Pmp22*	-2.0854
NR_027759	*Dctd*	-2.0946
NM_008613	*Mns1*	-2.0983
NM_001033409	*Lgr6*	-2.0991
NM_018827	*Crlf1*	-2.1
NM_001306148	*Car12*	-2.1068
NM_031380	*Fstl3*	-2.1098
NM_013589	*Ltbp2*	-2.1154
NM_001347100	*Cald1*	-2.1186
NM_001081020	*Adamts6*	-2.1235
NM_001271409	*Sorbs3*	-2.1308
NM_029352	*Dusp9*	-2.1309
NM_009528	*Wnt7b*	-2.1376
NM_029035	*Spsb1*	-2.1394
NM_173019	*Pfkfb4*	-2.1423
NM_001033213	*Ttc7b*	-2.1432
NR_028577	*D030025P21Rik*	-2.1462
NM_001025363	*Klc1*	-2.1466
NM_001277113	*Rpl22*	-2.1534
NM_001166454	*Pls3*	-2.1579
NM_001163189	*Enthd1*	-2.1592
NM_010770	*Matn3*	-2.1596
NM_001271419	*Ly6a*	-2.16
NM_019686	*Cib2*	-2.1625
NM_001111073	*Fxyd5*	-2.1647
NM_009848	*Entpd1*	-2.1656
NM_011171	*Procr*	-2.1692
NM_001204277	*Hdac7*	-2.1735
NM_176832	*Spire1*	-2.1744
NM_027341	*Dzip3*	-2.1818
NM_198193	*Raet1e*	-2.183
NM_177628	*Fam167a*	-2.1837
NM_027147	*Enho*	-2.1868
NM_008427	*Kcnj4*	-2.1893
NM_001037809	*Cdh3*	-2.1945
NM_170599	*Igsf11*	-2.1996
NM_008761	*Fxyd5*	-2.2007
NM_178595	*Ptrh1*	-2.2011
NM_178679	*Zfp365*	-2.2014
NM_001198977	*Syk*	-2.203
NM_020044	*Lat2*	-2.2051
NM_133187	*Fam198b*	-2.2055
NM_172453	*Pif1*	-2.2093
NM_001293766	*Pygo2*	-2.2117

NM_001198792	*Ak1*	-2.2158
NM_008773	*P2ry2*	-2.217
NM_001112735	*9930012K11Rik*	-2.2177
NR_132280	*Pold4*	-2.2228
NM_001164201	*Cers3*	-2.2228
NM_008132	*Glrp1*	-2.226
NM_008364	*Il1rap*	-2.2333
NM_001145801	*Ctla2b*	-2.2453
NM_001159402	*Upp1*	-2.263
NR_102291	*Rpl22*	-2.2632
NM_001130457	*Tbrg4*	-2.2677
NM_020293	*Cldn9*	-2.2767
NM_001077694	*Dysf*	-2.2773
NR_034155	*Olfr1372-ps1*	-2.279
NM_001160012	*Gjb3*	-2.2873
NM_001048142	*Bdnf*	-2.2909
NM_008196	*Gzmk*	-2.297
NM_001081291	*Ccdc88b*	-2.2977
NM_009154	*Sema5a*	-2.2999
NM_007984	*Fscn1*	-2.3004
NM_001163018	*Suz12*	-2.3167
NM_175307	*Fam46b*	-2.3183
NM_001285469	*Steap2*	-2.3239
NM_145520	*Trub2*	-2.33
NM_001174170	*Serpinb2*	-2.3304
NM_031159	*Apobec1*	-2.3348
NM_001033149	*Ttc9*	-2.3398
NM_007664	*Cdh2*	-2.3406
NM_028709	*Btbd11*	-2.3465
NM_001243041	*Adk*	-2.3514
NM_029806	*Lypd5*	-2.3638
NM_175440	*Prss27*	-2.3692
NM_134103	*Il1rap*	-2.3693
NM_001293637	*Nek1*	-2.3722
NM_199448	*Fez2*	-2.377
NM_172653	*Slc39a10*	-2.3898
NM_010566	*Inpp5d*	-2.3916
NM_027024	*Cst13*	-2.3934
NM_001313701	*Pkp1*	-2.3972
NM_001347212	*Bpnt1*	-2.3972
NM_001134391	*Apobec1*	-2.4129
NM_001134791	*Osbpl9*	-2.418
NM_028195	*Cyth4*	-2.4183
NM_001083810	*Prr5l*	-2.425
NM_001317243	*Mtch2*	-2.4264
NM_177082	*Sp8*	-2.4277
NM_001025378	*Orc2*	-2.4283
NM_001289717	*Bcl2l1*	-2.433
NM_011799	*Cdc6*	-2.4366
NM_145077	*Ucn2*	-2.4431
NM_030060	*Batf3*	-2.4491
NR_027485	*Psmd2*	-2.4511
NM_008430	*Kcnk1*	-2.4591
NM_001277076	*Fer1l5*	-2.4594
NM_001164190	*Mtm1*	-2.4654

NM_027399	*Steap1*	-2.4751
NM_001081346	*Rtkn2*	-2.4757
NM_026514	*Cdc42ep3*	-2.4786
NM_001083127	*Lhx6*	-2.4795
NM_001164611	*Smpd4*	-2.4816
NM_027571	*P2ry12*	-2.4877
NM_001136056	*Cntfr*	-2.4889
NM_001282045	*Schip1*	-2.4936
NM_010599	*Kcnab3*	-2.5
NM_025565	*Spc25*	-2.5001
NM_009560	*Zfp60*	-2.5037
NM_138751	*Tmem47*	-2.5041
NM_008462	*Klra2*	-2.506
NM_008940	*Klk8*	-2.507
NM_026415	*Cysrt1*	-2.5122
NM_029932	*Spns3*	-2.5147
NM_133922	*Krba1*	-2.515
NM_028536	*Ccsap*	-2.5154
NM_022879	*Myl7*	-2.5175
NM_010738	*Ly6a*	-2.5196
NM_001122899	*Lepr*	-2.5214
NM_001034962	*Sorbs1*	-2.5233
NR_045098	*Gm2115*	-2.524
NM_009779	*C3ar1*	-2.5255
NM_001166543	*Hmga1*	-2.5337
NR_045516	*Dlg1*	-2.5351
NR_045293	*D630045M09Rik*	-2.5432
NM_001042534	*Capg*	-2.5493
NM_025961	*Gatm*	-2.55
NM_001348478	*Dok7*	-2.55
NM_007400	*Adam12*	-2.5502
NM_001177577	*Gm1045*	-2.5514
NM_001164767	*Robo3*	-2.5535
NM_001347056	*Als2cr12*	-2.5547
NM_007472	*Aqp1*	-2.5554
NM_001291104	*Fgf11*	-2.5554
NM_025413	*Lce1g*	-2.5564
NM_020260	*Arhgap31*	-2.5566
NM_001243748	*Rassf1*	-2.5585
NR_045080	*3300005D01Rik*	-2.5588
NM_009016	*Raet1a*	-2.5609
NM_001163531	*Tmem175*	-2.5631
NM_009662	*Alox5*	-2.5675
NM_145478	*Pim3*	-2.5717
NM_001290706	*Tmem54*	-2.5733
NM_001242944	*Zfp965*	-2.5734
NM_008827	*Pgf*	-2.5785
NM_009050	*Ret*	-2.5818
NM_007584	*Ddr1*	-2.5834
NM_001024837	*Adarb1*	-2.5979
NM_177354	*Vash1*	-2.6003
NM_028770	*Krt80*	-2.6026
NM_001025361	*Klc1*	-2.6051
NM_011923	*Angptl2*	-2.6088
NR_038025	*4933412E12Rik*	-2.6107

NM_001310446	*Dab2*	-2.6145
NM_001168652	*Lrch4*	-2.626
NM_010217	*Ctgf*	-2.6391
NM_001310593	*Mef2d*	-2.6431
NM_010808	*Mmp24*	-2.6461
NM_199195	*Bckdhb*	-2.6507
NR_028427	*Mirt1*	-2.6565
NM_009257	*Serpinb5*	-2.6651
NM_172506	*Boc*	-2.6665
NM_001177774	*Fbxw7*	-2.6688
NM_010720	*Lipg*	-2.673
NM_001204280	*Hdac7*	-2.6747
NM_001276301	*Ampd3*	-2.6754
NM_029523	*Depdc1a*	-2.6805
NM_019564	*Htra1*	-2.6888
NM_008979	*Ptpn22*	-2.6924
NM_001043355	*Map6*	-2.7066
NM_009378	*Thbd*	-2.7093
NM_001168497	*Plscr3*	-2.7135
NM_001195538	*Dclk1*	-2.7183
NM_001310515	*Mbnl3*	-2.7254
NM_013468	*Ankrd1*	-2.7286
NM_029774	*Ttll11*	-2.7316
NM_001163337	*Atp2a3*	-2.7372
NM_199024	*Nol4*	-2.7389
NM_001081172	*Frmpd1*	-2.7398
NM_001162989	*Phax*	-2.7408
NM_001199305	*Atxn1*	-2.7409
NM_172826	*Dact2*	-2.7421
NM_001164628	*Arhgap8*	-2.7453
NM_011374	*St8sia1*	-2.7521
NM_001271589	*Eps8*	-2.7531
NM_178793	*Ccbe1*	-2.7534
NM_001285940	*Fez2*	-2.7624
NM_013592	*Matn4*	-2.7661
NM_027756	*Mfap3l*	-2.7672
NM_009255	*Serpine2*	-2.773
NM_028877	*Palm3*	-2.7745
NM_022964	*Lat2*	-2.7822
NM_013912	*Apln*	-2.7857
NM_001285867	*C77080*	-2.7923
NM_001048167	*Map6*	-2.8104
NM_001164661	*Cyfip1*	-2.8193
NM_008380	*Inhba*	-2.8289
NM_023118	*Dab2*	-2.8334
NM_001166394	*4931428F04Rik*	-2.8343
NM_001105246	*Pcdh19*	-2.8451
NM_175752	*Chn1*	-2.8494
NM_001002897	*Atg9b*	-2.8503
NM_177897	*B4galnt4*	-2.8596
NM_134250	*Havcr2*	-2.8737
NM_139298	*Wnt9a*	-2.8775
NM_010729	*Loxl1*	-2.8776
NM_001177371	*Dbn1*	-2.8905
NM_173395	*Fam132b*	-2.8946

NM_033079	*M1ap*	-2.9198
NM_001271407	*Sorbs3*	-2.9203
NM_029620	*Pcolce2*	-2.9251
NM_001160208	*Blzf1*	-2.9252
NR_033145	*Dand5*	-2.9289
NM_001161714	*Tgm1*	-2.9298
NM_177311	*Serac1*	-2.9409
NM_001013368	*E2f8*	-2.9741
NM_019456	*Apbb1ip*	-2.9898
NM_001080552	*Gsg1*	-3.002
NM_026050	*Fam220a*	-3.0028
NM_133186	*Steap3*	-3.0417
NM_028293	*Cep55*	-3.0435
NM_022883	*Lpin3*	-3.048
NM_001099785	*D3Ertd751e*	-3.0687
NM_001313962	*Rhoa*	-3.082
NM_178928	*Afap1l1*	-3.0838
NM_019756	*Tubd1*	-3.0851
NM_009985	*Ctsw*	-3.0863
NM_007424	*Acan*	-3.088
NM_009616	*Adam19*	-3.0927
NM_028623	*Cst6*	-3.0953
NM_028386	*Asphd2*	-3.0957
NM_008288	*Hsd11b1*	-3.1075
NM_054044	*Adgra2*	-3.1175
NM_001168257	*Tmem40*	-3.1619
NM_001291449	*Foxred1*	-3.1731
NM_001080797	*G3bp2*	-3.1994
NM_021544	*Scn5a*	-3.2287
NM_001198635	*Map7*	-3.2412
NM_011836	*Lamc3*	-3.2442
NM_011446	*Sox7*	-3.2501
NM_011406	*Slc8a1*	-3.2562
NM_001255992	*Bbs12*	-3.2583
NM_001310428	*Crocc2*	-3.2652
NM_023279	*Tubb3*	-3.2852
NM_001177847	*Tirap*	-3.2876
NM_001310438	*Ly6g*	-3.3065
NM_029346	*Dynap*	-3.3146
NM_175460	*Nmnat2*	-3.321
NM_001166399	*Rab3a*	-3.3567
NM_001256083	*Myo7a*	-3.3633
NM_176860	*Ubash3b*	-3.3659
NM_001159940	*2310007B03Rik*	-3.3779
NM_001291210	*Rgs19*	-3.3819
NM_001163422	*Tatdn3*	-3.3823
NM_030723	*Pum2*	-3.4075
NM_013569	*Kcnh2*	-3.416
NM_001347036	*Lrp12*	-3.4195
NM_008216	*Has2*	-3.4504
NM_008478	*L1cam*	-3.4686
NM_001348274	*Dok7*	-3.4832
NM_001145979	*Gtpbp2*	-3.4906
NM_011452	*Serpinb9b*	-3.4934
NM_133712	*Klk10*	-3.5137

NM_025944	*Tmem246*	-3.5532
NM_021397	*Zbtb32*	-3.5571
NM_130886	*Card14*	-3.5698
NM_007474	*Aqp8*	-3.5831
NM_001142959	*Bcl2l15*	-3.6521
NM_001313956	*Krt14*	-3.6651
NR_045744	*Gm20744*	-3.6685
NM_027551	*Klhl30*	-3.67
NM_001159284	*Smtn*	-3.7097
NM_012009	*Sh2d1b1*	-3.7487
NR_045482	*1810020O05Rik*	-3.7619
NM_001291055	*Lgals8*	-3.7636
NM_030179	*Clip4*	-3.7827
NM_019645	*Pkp1*	-3.7953
NM_001142960	*Bcl2l15*	-3.8117
NM_001308167	*Sh3bp5l*	-3.8452
NM_008054	*Fyn*	-3.8575
NM_010607	*Kcnk2*	-3.8585
NM_008856	*Prkch*	-3.8677
NM_001080780	*Ret*	-3.893
NM_207706	*Elmo2*	-3.8959
NM_009527	*Wnt7a*	-3.9415
NM_199422	*S100a7a*	-3.9643
NM_001161516	*Dctd*	-4.0312
NM_001347582	*Eml2*	-4.0631
NM_001083125	*Lhx6*	-4.096
NM_001024955	*Pik3r1*	-4.1105
NM_001042556	*Rpf2*	-4.1171
NM_010875	*Ncam1*	-4.1537
NM_022982	*Rtn4r*	-4.1645
NM_001310678	*Ltbp4*	-4.2224
NM_001163553	*Ap4b1*	-4.2492
NR_027352	*Vash2*	-4.2529
NM_001013384	*Podnl1*	-4.2562
NM_007738	*Col7a1*	-4.2662
NM_001163512	*Rgs12*	-4.2961
NM_001109045	*Aqp8*	-4.3339
NR_045275	*8430429K09Rik*	-4.3409
NM_001164677	*Pdcd6ip*	-4.3917
NM_028266	*Col16a1*	-4.4328
NM_001135100	*Il34*	-4.5401
NM_011111	*Serpinb2*	-4.5486
NM_011267	*Rgs16*	-4.5699
NM_016958	*Krt14*	-4.5753
NM_001271538	*Myh14*	-4.9
NM_010837	*Map6*	-4.9282
NM_172900	*Siglecg*	-4.9758
NM_001291016	*Bcl2l11*	-5.0609
NM_026580	*Otub2*	-5.1457
NM_016673	*Cntfr*	-5.1747
NM_008381	*Inhbb*	-5.2248
NR_028267	*Fcho1*	-5.25
NM_010626	*Kif7*	-5.2723
NM_007985	*Fancc*	-5.31
NM_145602	*Ndrg4*	-5.3237

NM_053095	*Il24*	-5.3358
NM_144805	*Tmem40*	-5.3401
NM_011518	*Syk*	-5.3654
NM_001289522	*Cad*	-5.416
NM_001253768	*Ssx2ip*	-5.5239
NM_001199304	*Atxn1*	-5.5808
NM_001281818	*Specc1*	-5.6122
NM_001177556	*Gng12*	-5.8476
NM_001177559	*Gng12*	-5.8989
NM_015804	*Atp11a*	-5.9009
NM_001331048	*Has3*	-6.0247
NM_001285817	*Dtna*	-6.0796
NM_001324398	*Klk8*	-6.2793
NM_001286370	*Ghr*	-6.4543
NM_001168600	*Gnl3l*	-7.0675
NM_001110315	*Kif1a*	-7.2449
NM_001291212	*Myo18a*	-7.7778
NM_001081445	*Ncam1*	-7.935
NM_001164498	*Papd5*	-8.8496
NM_007917	*Eif4e*	-10.3814

Table S6. Significantly altered canonical pathways in MDA-F471 G1 spheres relative to parental cells.

Canonical pathways	P-value
LPS/IL-1 Mediated Inhibition of RXR Function	0.0000000001
Acute Phase Response Signaling	0.0000000069
Granulocyte Adhesion and Diapedesis	0.0000000170
Xenobiotic Metabolism Signaling	0.0000001905
TNFR2 Signaling	0.0000002884
Role of Osteoblasts, Osteoclasts and Chondrocytes in Rheumatoid Arthritis	0.0000003548
Hepatic Fibrosis / Hepatic Stellate Cell Activation	0.0000003715
Agranulocyte Adhesion and Diapedesis	0.0000009120
Molecular Mechanisms of Cancer	0.0000012303
Colorectal Cancer Metastasis Signaling	0.0000053703
Role of Macrophages, Fibroblasts and Endothelial Cells in Rheumatoid Arthritis	0.0000112202
iNOS Signaling	0.0000173780
Apoptosis Signaling	0.0000177828
Aryl Hydrocarbon Receptor Signaling	0.0000190546
Leukocyte Extravasation Signaling	0.0000229087
Axonal Guidance Signaling	0.0000263027
IL-10 Signaling	0.0000301995
Nicotine Degradation II	0.0000446684
LXR/RXR Activation	0.0000478630
Induction of Apoptosis by HIV1	0.0000660693
Nicotine Degradation III	0.0000776247
Death Receptor Signaling	0.0000933254
NF-κB Signaling	0.0000977237
FXR/RXR Activation	0.0001000000
Macropinocytosis Signaling	0.0001023293
PPAR Signaling	0.0001318257
PXR/RXR Activation	0.0001584893
Lymphotoxin β Receptor Signaling	0.0002344229
Cell Cycle: G2/M DNA Damage Checkpoint Regulation	0.0002691535
GP6 Signaling Pathway	0.0002951209
IL-6 Signaling	0.0003311311
Toll-like Receptor Signaling	0.0003890451
Production of Nitric Oxide and Reactive Oxygen Species in Macrophages	0.0003981072
IL-17A Signaling in Fibroblasts	0.0004073803
Superpathway of Melatonin Degradation	0.0004073803
Coagulation System	0.0004073803
TWEAK Signaling	0.0004073803
Extrinsic Prothrombin Activation Pathway	0.0004897788
Hepatic Cholestasis	0.0005128614
Melatonin Degradation I	0.0005128614
IL-8 Signaling	0.0005248075

PI3K/AKT Signaling	0.0005370318
Angiopoietin Signaling	0.0005495409
Small Cell Lung Cancer Signaling	0.0005888437
Estrogen-mediated S-phase Entry	0.0006165950
Glioblastoma Multiforme Signaling	0.0006309573
Sertoli Cell-Sertoli Cell Junction Signaling	0.0007244360
TNFR1 Signaling	0.0009549926
ILK Signaling	0.0010715193
Role of Tissue Factor in Cancer	0.0011220185
April Mediated Signaling	0.0011220185
Serotonin Degradation	0.0012882496
Type I Diabetes Mellitus Signaling	0.0012882496
HMGB1 Signaling	0.0013803843
NRF2-mediated Oxidative Stress Response	0.0014791084
DNA damage-induced 14-3-3σ Signaling	0.0016595869
Basal Cell Carcinoma Signaling	0.0016595869
CD40 Signaling	0.0017378008
Estrogen Biosynthesis	0.0017782794
Human Embryonic Stem Cell Pluripotency	0.0018620871
Osteoarthritis Pathway	0.0019952623
PEDF Signaling	0.0020892961
Tight Junction Signaling	0.0021379621
Bupropion Degradation	0.0021877616
Ethanol Degradation IV	0.0021877616
Cyclins and Cell Cycle Regulation	0.0023442288
Glutathione-mediated Detoxification	0.0025118864
Neuroinflammation Signaling Pathway	0.0025703958
Virus Entry via Endocytic Pathways	0.0026302680
Ethanol Degradation II	0.0026302680
Cell Cycle Regulation by BTG Family Proteins	0.0026302680
Superpathway of Serine and Glycine Biosynthesis I	0.0026915348
Role of IL-17A in Arthritis	0.0028840315
p53 Signaling	0.0029512092
Oxidative Ethanol Degradation III	0.0032359366
4-1BB Signaling in T Lymphocytes	0.0032359366
Neuroprotective Role of THOP1 in Alzheimer's Disease	0.0032359366
Wnt/β-catenin Signaling	0.0033884416
Atherosclerosis Signaling	0.0034673685
Integrin Signaling	0.0034673685
IL-17A Signaling in Airway Cells	0.0038904514
Histidine Degradation III	0.0050118723
CD27 Signaling in Lymphocytes	0.0051286138
Putrescine Degradation III	0.0056234133
Cell Cycle: G1/S Checkpoint Regulation	0.0056234133
B Cell Activating Factor Signaling	0.0058884366

PCP pathway	0.0063095734
Intrinsic Prothrombin Activation Pathway	0.0070794578
Gα12/13 Signaling	0.0074131024
Acetone Degradation I (to Methylglyoxal)	0.0075857758
Chronic Myeloid Leukemia Signaling	0.0083176377
Ovarian Cancer Signaling	0.0083176377
Citrulline Biosynthesis	0.0083176377
Tetrahydrofolate Salvage from 5,10-methenyltetrahydrofolate	0.0085113804
Serine Biosynthesis	0.0085113804
Folate Polyglutamylation	0.0085113804
Glioma Invasiveness Signaling	0.0085113804
Reelin Signaling in Neurons	0.0089125094
IL-1 Signaling	0.0089125094
Dopamine Degradation	0.0091201084
Tryptophan Degradation X (Mammalian, via Tryptamine)	0.0093325430
Spermine Biosynthesis	0.0100000000
Spermidine Biosynthesis I	0.0100000000
Type II Diabetes Mellitus Signaling	0.0100000000
NF-κB Activation by Viruses	0.0114815362
LPS-stimulated MAPK Signaling	0.0114815362
Iron homeostasis signaling pathway	0.0123026877
Mitotic Roles of Polo-Like Kinase	0.0123026877
Regulation of the Epithelial-Mesenchymal Transition Pathway	0.0125892541
Germ Cell-Sertoli Cell Junction Signaling	0.0125892541
HER-2 Signaling in Breast Cancer	0.0128824955
Inhibition of Matrix Metalloproteases	0.0128824955
Bladder Cancer Signaling	0.0128824955
PTEN Signaling	0.0138038426
Thyroid Cancer Signaling	0.0151356125
Noradrenaline and Adrenaline Degradation	0.0151356125
ATM Signaling	0.0165958691
UVA-Induced MAPK Signaling	0.0169824365
Role of PKR in Interferon Induction and Antiviral Response	0.0177827941
Fatty Acid α-oxidation	0.0177827941
Dendritic Cell Maturation	0.0177827941
Adrenomedullin signaling pathway	0.0177827941
Role of NANOG in Mammalian Embryonic Stem Cell Pluripotency	0.0177827941
Actin Cytoskeleton Signaling	0.0177827941
PPARα/RXRα Activation	0.0204173794
Ceramide Signaling	0.0208929613
RAR Activation	0.0229086765
Clathrin-mediated Endocytosis Signaling	0.0234422882
RANK Signaling in Osteoclasts	0.0234422882
Thyroid Hormone Metabolism II (via Conjugation and/or Degradation)	0.0239883292
MIF Regulation of Innate Immunity	0.0239883292

Adipogenesis pathway	0.0245470892
Relaxin Signaling	0.0245470892
Phosphatidylcholine Biosynthesis I	0.0257039578
B Cell Receptor Signaling	0.0263026799
Amyotrophic Lateral Sclerosis Signaling	0.0263026799
Complement System	0.0269153480
Amyloid Processing	0.0269153480
Thiosulfate Disproportionation III (Rhodanese)	0.0275422870
Sumoylation Pathway	0.0275422870
Erythropoietin Signaling	0.0288403150
ERK5 Signaling	0.0288403150
Tec Kinase Signaling	0.0309029543
PDGF Signaling	0.0323593657
Mouse Embryonic Stem Cell Pluripotency	0.0331131121
IL-17A Signaling in Gastric Cells	0.0331131121
Histamine Degradation	0.0346736850
Ubiquinol-10 Biosynthesis (Eukaryotic)	0.0346736850
Dermatan Sulfate Biosynthesis (Late Stages)	0.0354813389
IL-15 Signaling	0.0363078055
GDNF Family Ligand-Receptor Interactions	0.0363078055
Superoxide Radicals Degradation	0.0380189396
ErbB2-ErbB3 Signaling	0.0389045145
Role of Wnt/GSK-3β Signaling in the Pathogenesis of Influenza	0.0407380278
autophagy	0.0416869383
Protein Kinase A Signaling	0.0416869383
Inflammasome pathway	0.0426579519
VDR/RXR Activation	0.0436515832
P2Y Purigenic Receptor Signaling Pathway	0.0436515832
Caveolar-mediated Endocytosis Signaling	0.0467735141
Cell Cycle Control of Chromosomal Replication	0.0478630092
Retinoate Biosynthesis I	0.0478630092

Table S7. Representative upstream regulators that were predicted to be significantly activated in MDA-F471 G1 spheres relative to parental cells.

Upstream regulator	Molecule type	Activation z-score	p-value
**IL1β**	cytokine	5.89	1.35×10^{-24}
**NFκB (complex)**	complex	5.38	8.76×10^{-14}
**IFNγ**	cytokine	4.81	3.91×10^{-18}
**FOXO3**	transcription regulator	4.74	3.34×10^{-28}
**RELA**	transcription regulator	4.51	4.95×10^{-12}
**TNF**	cytokine	4.44	1.43×10^{-34}
**NFE2L2**	transcription regulator	3.80	3.07×10^{-8}
**GSK3β**	kinase	2.14	9.09×10^{-5}

REFERENCES

1. Kim, E., et al., *Profiling of transcripts and proteins modulated by the E7 oncogene in the lung tissue of E7-Tg mice by the omics approach.* Mol Med Rep, 2009. **2**(1): p. 129-37.
2. Levi, B.P., et al., *Aldehyde dehydrogenase 1a1 is dispensable for stem cell function in the mouse hematopoietic and nervous systems.* Blood, 2009. **113**(8): p. 1670-80.
3. Outhwaite, J.E., et al., *Expression of aldehyde dehydrogenase family 1, member A3 in glycogen trophoblast cells of the murine placenta.* Placenta, 2015. **36**(3): p. 304-11.
4. Yang, Y., et al., *beta-Arrestin1 enhances hepatocellular carcinogenesis through inflammation-mediated Akt signalling.* Nat Commun, 2015. **6**: p. 7369.
5. Kim, D.E., et al., *Convergent roles of ATF3 and CSL in chromatin control of cancer-associated fibroblast activation.* J Exp Med, 2017. **214**(8): p. 2349-2368.
6. Sato, T., et al., *c-Jun N-terminal kinase in pancreatic tumor stroma augments tumor development in mice.* Cancer Sci, 2017. **108**(11): p. 2156-2165.
7. Donega, V., et al., *Intranasal administration of human MSC for ischemic brain injury in the mouse: in vitro and in vivo neuroregenerative functions.* PLoS One, 2014. **9**(11): p. e112339.
8. Tanaka, M., et al., *Mouse hepatoblasts at distinct developmental stages are characterized by expression of EpCAM and DLK1: drastic change of EpCAM expression during liver development.* Mech Dev, 2009. **126**(8-9): p. 665-76.
9. Patin, E.C., et al., *Type I IFN Receptor Signaling Controls IL7-Dependent Accumulation and Activity of Protumoral IL17A-Producing gammadeltaT Cells in Breast Cancer.* Cancer Res, 2018. **78**(1): p. 195-204.
10. Mavis, C.K., et al., *Expression level and DNA methylation status of glutathione-S-transferase genes in normal murine prostate and TRAMP tumors.* Prostate, 2009. **69**(12): p. 1312-24.
11. Sahoo, A., et al., *Stat6 and c-Jun mediate Th2 cell-specific IL-24 gene expression.* J Immunol, 2011. **186**(7): p. 4098-109.
12. Wang, X., et al., *Lyn regulates mucus secretion and MUC5AC via the STAT6 signaling pathway during allergic airway inflammation.* Sci Rep, 2017. **7**: p. 42675.
13. Gong, H., et al., *Evaluation of candidate reference genes for RT-qPCR studies in three metabolism related tissues of mice after caloric restriction.* Sci Rep, 2016. **6**: p. 38513.
14. Basso, M., et al., *Transglutaminase inhibition protects against oxidative stress-induced neuronal death downstream of pathological ERK activation.* J Neurosci, 2012. **32**(19): p. 6561-9.
15. Sha, H., et al., *Adipocyte spliced form of X-box-binding protein 1 promotes adiponectin multimerization and systemic glucose homeostasis.* Diabetes, 2014. **63**(3): p. 867-79.
16. Tawk, M., et al., *Wnt/beta-catenin signaling is an essential and direct driver of myelin gene expression and myelinogenesis.* J Neurosci, 2011. **31**(10): p. 3729-42.
17. Yan, J., et al., *Aldehyde dehydrogenase 3A1 associates with prostate tumorigenesis.* Br J Cancer, 2014. **110**(10): p. 2593-603.
18. Marcato, P., et al., *Aldehyde dehydrogenase activity of breast cancer stem cells is primarily due to isoform ALDH1A3 and its expression is predictive of metastasis.* Stem Cells, 2011. **29**(1): p. 32-45.
19. Li, X., et al., *Activating transcription factor 3 promotes malignance of lung cancer cells in vitro.* Thorac Cancer, 2017. **8**(3): p. 181-191.
20. Luan, L., et al., *Comparative Transcriptome Profiles of Human Blood in Response to the Toll-like Receptor 4 Ligands Lipopolysaccharide and Monophosphoryl Lipid A.* Sci Rep, 2017. **7**: p. 40050.

21. Pasini, B., et al., *Clinical and molecular genetics of patients with the Carney-Stratakis syndrome and germline mutations of the genes coding for the succinate dehydrogenase subunits SDHB, SDHC, and SDHD.* Eur J Hum Genet, 2008. **16**(1): p. 79-88.
22. Fehrenbach, S., et al., *Loss of tumorigenic potential upon transdifferentiation from keratinocytic into melanocytic lineage.* Sci Rep, 2016. **6**: p. 28891.
23. Lemma, S., et al., *Identification and Validation of Housekeeping Genes for Gene Expression Analysis of Cancer Stem Cells.* PLoS One, 2016. **11**(2): p. e0149481.
