# Supplementary material for: Genome-Wide and Phenotypic Evaluation of Stem Cell Progenitors Derived From Gprc5a-Deficient Murine Lung Adenocarcinoma With Somatic Kras Mutations
Source: Front Oncol. 2019 Apr 2;9:207. doi: 10.3389/fonc.2019.00207 (PMC6454871; doi:10.3389/fonc.2019.00207)
Supplement: Supplementary file 2 [file Data_Sheet_2.PDF]

Supplementary figure 1

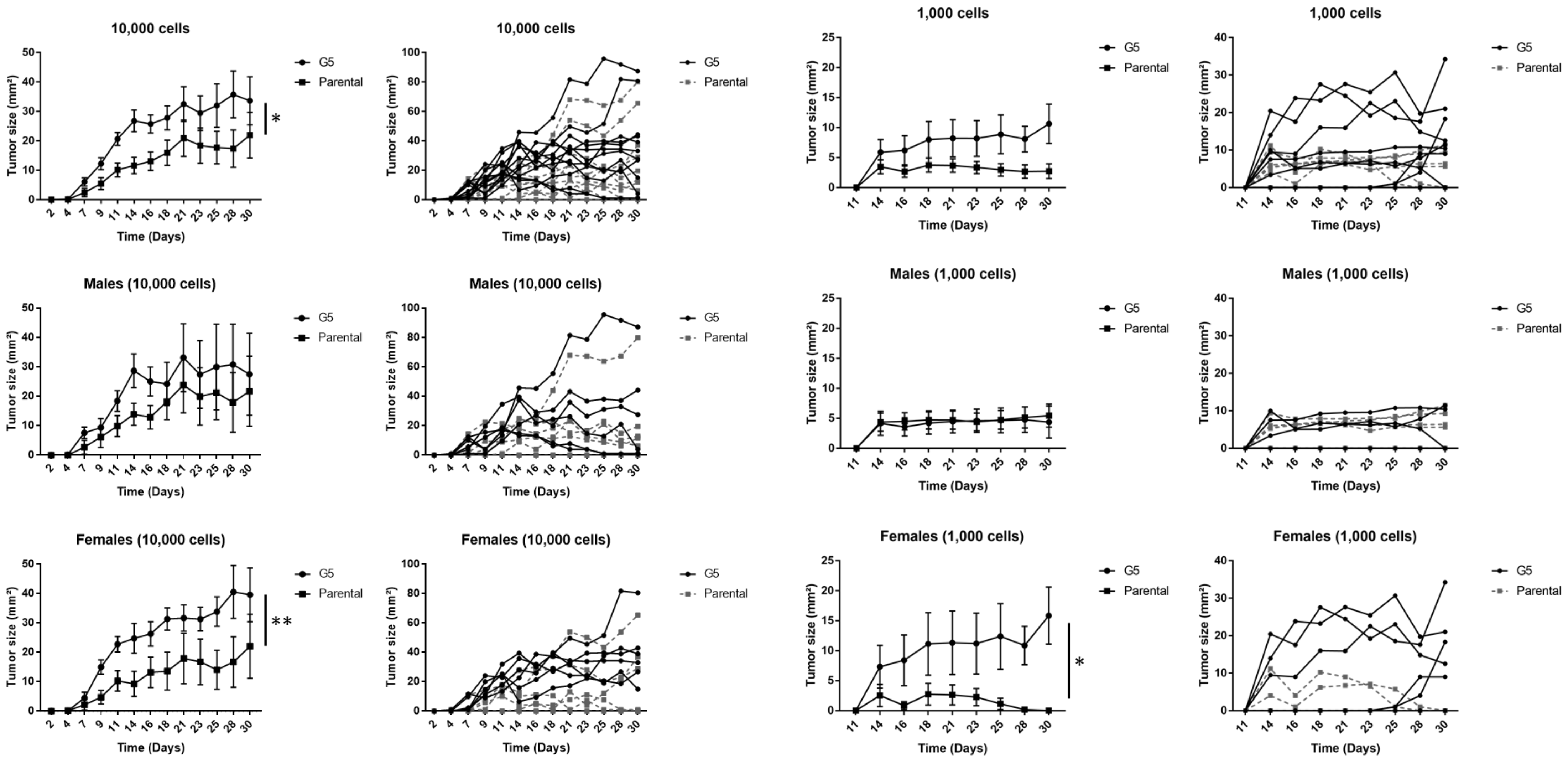

Figure S1. *In vivo* growth of xenotransplanted dissociated MDA-F471 G5 spheres and parental cells separated by gender. Two different cell dilutions (10,000 and 1,000) of either MDA-F471 parental cells or dissociated G5 spheres were suspended in 200 µl of a 1:1 mixture of serum-free DMEM F-12 media and growth factor-reduced Matrigel™ and subcutaneously injected into the right flank of 11 or 12 mice per group. Lengths and widths of tumors were measured three times per week for four weeks and tumor sizes were calculated according to the formula: tumor size (mm^2) = length x width. Average values of areas under the curve (AUCs) were calculated for each group were statistically analyzed using Student's t-test. Tumor sizes are plotted in line graphs as means ± SEM (*$P < 0.05$; **$P < 0.01$) (left) or as individual values per mouse (right).

Supplementary figure 2

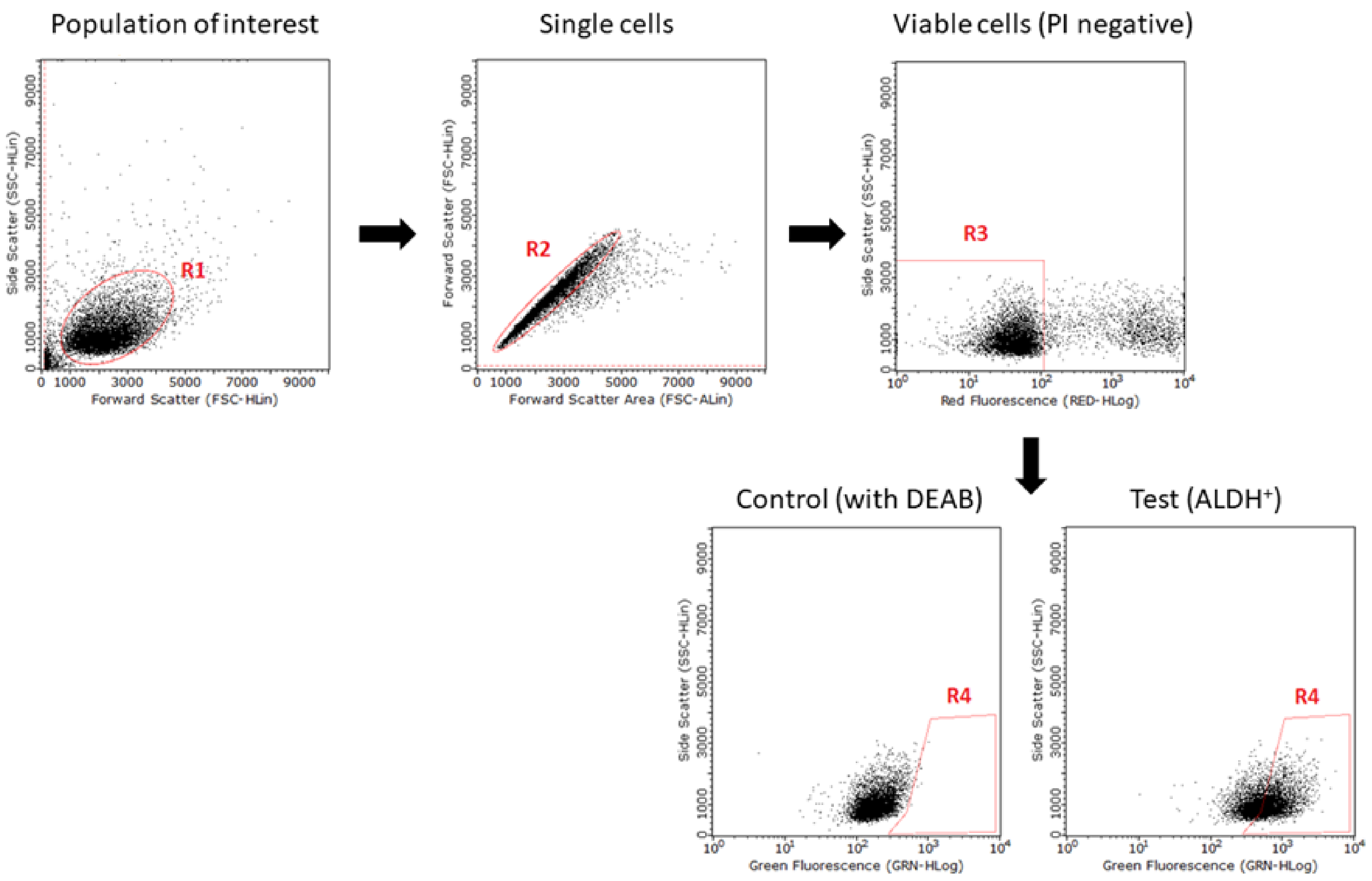

Figure S2. Gating strategy used for the flow cytometry analysis of the ALDEFLUOR assay. All samples were analyzed by sequential gating including main population (R1), single cells (R2) and viable (PI negative) cells (R3). Controls with DEAB inhibitor were used to set a gate (R4), which helps to identify the ALDH+ subpopulation in the test samples.

Supplementary figure 3

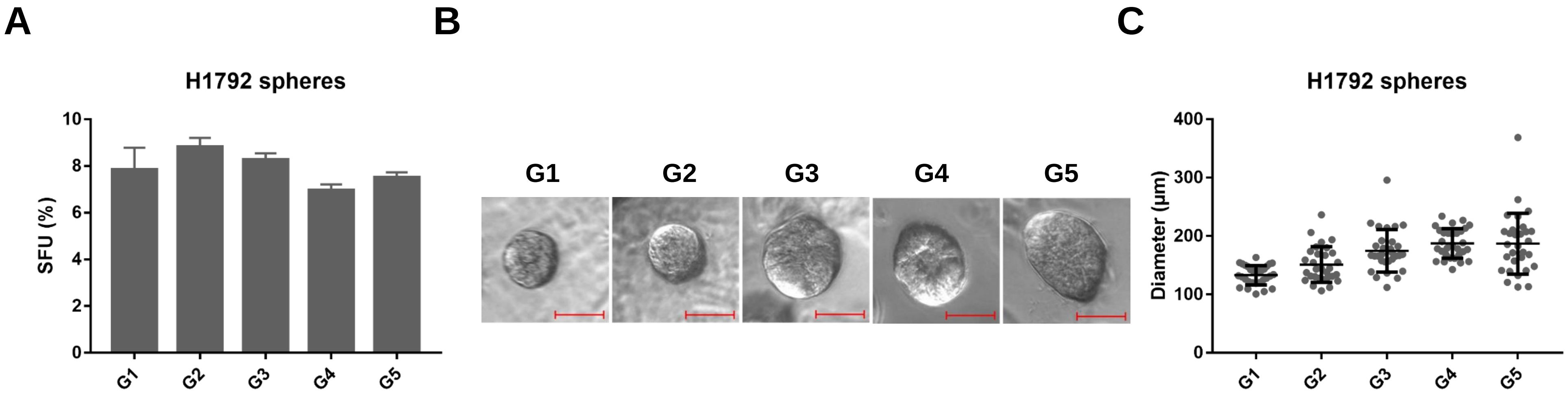

Figure S3. Sphere formation abilities of H1792 *KRAS* mutant LUAD cells. Single cell suspensions of H1792 cells (see Materials and Methods) were embedded in Matrigel™ and plated at the rim of each well. Media containing 5% FBS was added in the middle of each well. Cells were incubated for 6-7 days until they formed spheres. Sphere forming units were calculated as percentages of the number of formed spheres relative to the number of seeded cells. To assess the self-renewal ability of sphere forming cells, spheres were propagated for up to five generations. **A.** Sphere forming units (SFUs) of H1792 cells across five generations. Data was statistically analyzed by Kruskal-Wallis test. Error bars represent standard deviations between technical triplicates of each of three independent experiments. **B.** Representative bright-field images of H1792 G1-G5 spheres visualized by Axiovert inverted microscope at 10X magnification and analyzed by Carl Zeiss Zen 2 image software. Scale bar = 100 µm. **C.** Scatter plots of the diameters (µm) of 30 spheres from G1 up to G5 for H1792 cells. The data are reported as mean ± SD ($P < 0.0001$ by one-way ANOVA).

Supplementary figure 4

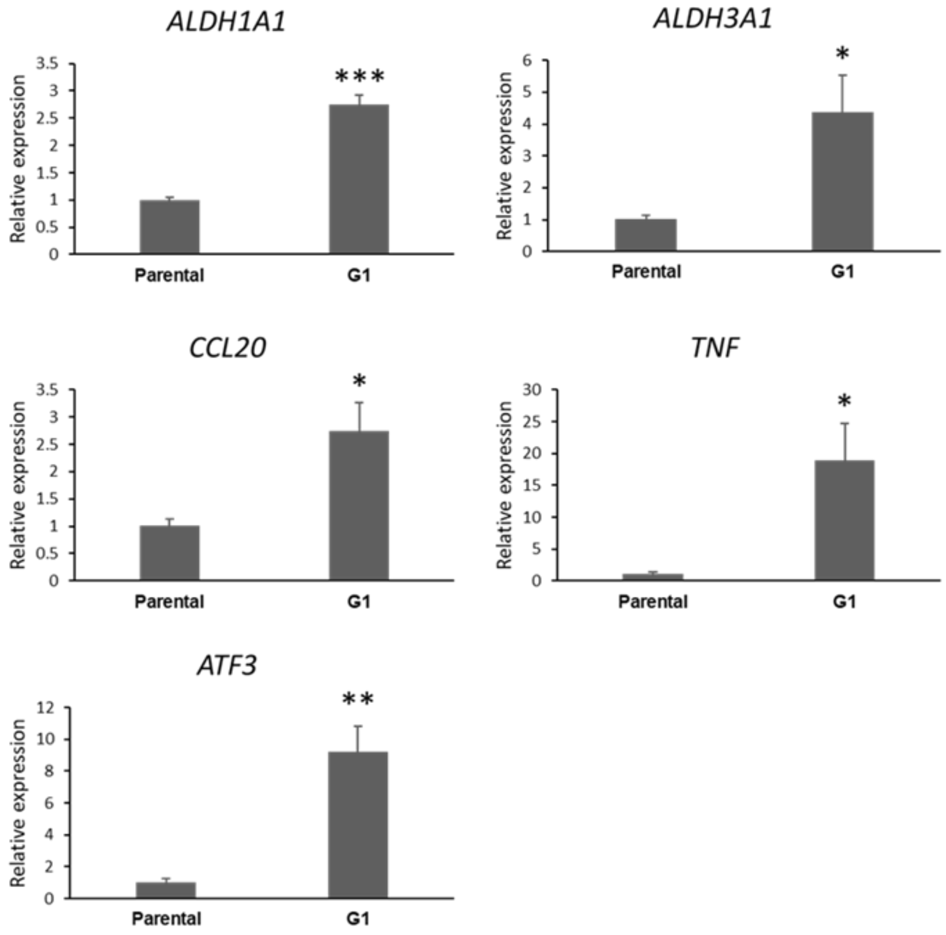

Figure S4. Validation of representative genes significantly differentially expressed between H1792 G1 spheres and parental cells by qRT-PCR. Upregulation of select genes was validated in H1792 G1, G3 and G5 spheres relative to parental cells by qRT-PCR and analyzed using the $2^{-\Delta\Delta Ct}$ calculation method by normalization to the average of two reference genes (*GAPDH* and *TBP*) and is presented as means + SEM (n=3). Statistical analysis was done using Student's t-test (**P* < 0.05; ***P* < 0.01; ****P* < 0.001).

Supplementary figure 5

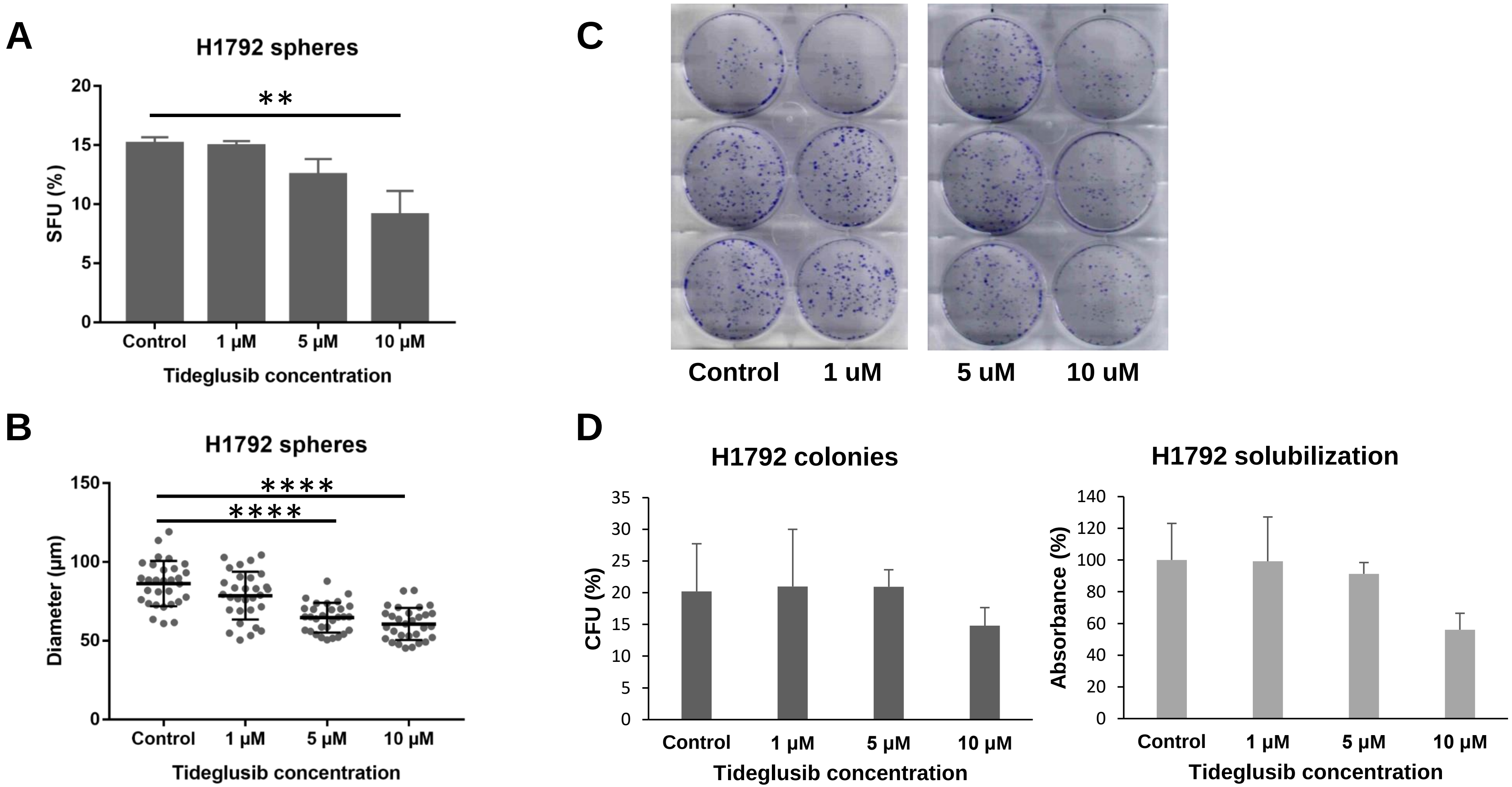

Figure S5. Effect of tideglusib on sphere and colony forming ability of human *KRAS*-mutant LUAD cells. H1792 cells were cultured in Matrigel™ for one week. Formed spheres were propagated to G2 and treated with control (DMSO), 1, 5, and 10 µM of tideglusib every two to three days. **A.** Sphere forming units (SFUs) were calculated for each condition as percentage of the number of formed spheres relative to number of seeded cells. Data were statistically analyzed by Dunn's post-hoc test following Kruskal-Wallis for biological triplicates and technical duplicates (***P* < 0.01; *****P* < 0.0001). **B.** Diameters of 30 spheres per condition were measured using Carl Zeiss Zen 2 image software and analyzed by one-way ANOVA. Colony formation assay of adherent H1792 cells was performed as described in the Materials and Methods section (**C-D**). Cells were seeded in triplicates at 500 cells/well in six-well plates and treated with varying concentrations of tideglusib every two to three days for seven days. Scanned images of the cultured adherent cells are depicted in **C.** Colonies were counted and colony forming units (CFUs) were calculated for each condition as follows: CFU = 100 x (number of colonies/500) (**D**, left). Spectrophotometric analysis of solubilized crystal violet-stained colonies was performed as described in the Methods section (**D**, right). Differences in number of colonies and absorbance between the different conditions were statistically analyzed using Dunn's post-hoc test following Kruskal-Wallis (***P* < 0.01; *****P* < 0.0001).
